# Synthesis and Photophysical Properties of Charge-Transfer-Based Pyrimidine-Derived α-Amino Acids

**DOI:** 10.1021/acs.joc.3c01437

**Published:** 2023-08-25

**Authors:** Sineenard Songsri, Alexander H. Harkiss, Andrew Sutherland

**Affiliations:** School of Chemistry, The Joseph Black Building, University of Glasgow, Glasgow G12 8QQ, United Kingdom

## Abstract

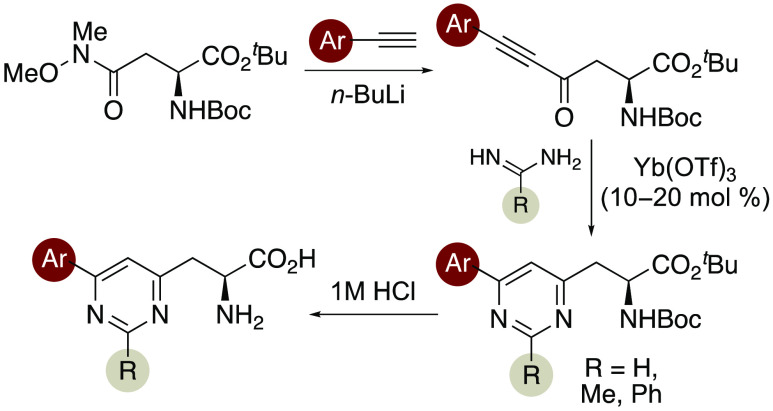

The four-step synthesis of fluorescent pyrimidine-derived α-amino acids from an l-aspartic acid derivative is described. The key synthetic steps involved preparation of ynone intermediates via the reaction of alkynyl lithium salts with a Weinreb amide, followed by an ytterbium-catalyzed heterocyclization reaction with amidines. Variation of substituents at the C2- and C4-position of the pyrimidine ring allowed tuning of the photoluminescent properties of the α-amino acids. This revealed that a combination of highly conjugated or electron-rich aryl substituents with the π-deficient pyrimidine motif resulted in fluorophores with the highest quantum yields and overall brightness. Further analysis of the most fluorogenic α-amino acid demonstrated solvatochromism and sensitivity to pH.

## Introduction

The importance of α-amino acids as the building blocks of life along with their role in fundamental biological processes continues to drive new discoveries and applications of unnatural analogues.^1^ In organic chemistry, nonproteinogenic α-amino acids are widely used in synthesis as the chiral component of ligands and auxiliaries for novel asymmetric methods, while readily available proteinogenic α-amino acids are used as chiral starting materials in total synthesis.^2^ In medicinal chemistry and chemical biology, unnatural α-amino acids are used as enzyme inhibitors and as probes to study biological mechanisms, protein–protein interactions, and peptide conformations.^3^

**Figure 1:**
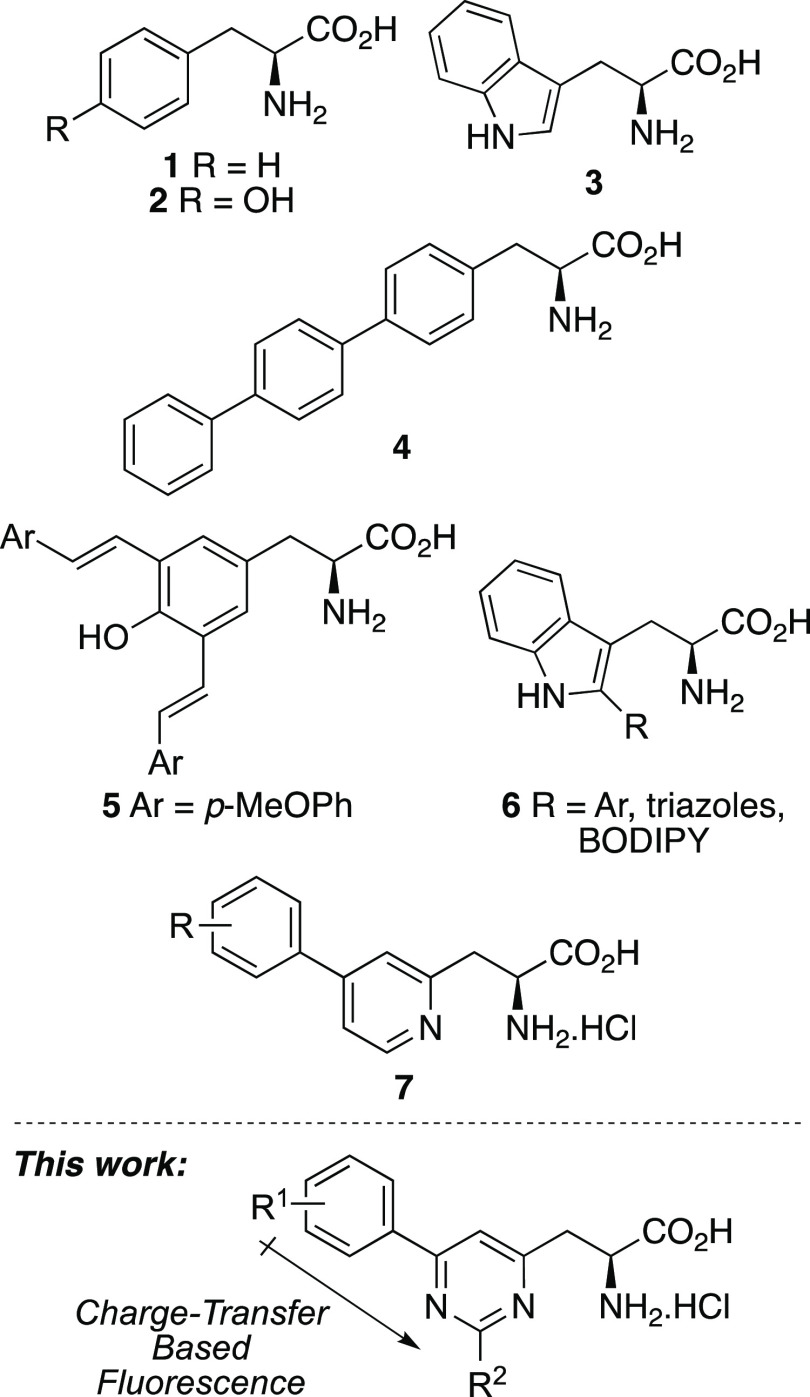
Fluorescent, proteinogenic α-amino acids and selected unnatural mimics.

In combination with the continued advances of fluorescence spectroscopy techniques, which allow the study of biological processes and the imaging of cellular processes,^4^ there has been significant recent interest in the development of unnatural α-amino acids as fluorescent probes.^5^ This is partly due to the limitations of other approaches. The attachment of large extrinsic fluorescent labels to a protein such as green fluorescent protein^6^ or commercially available chromophores can alter structure and function. The naturally occurring proteinogenic α-amino acids, phenylalanine **1**, tyrosine **2**, and tryptophan **3**, have poor photoluminescent properties and the presence of these at multiple sites and in different environments within a protein complicates analysis (Figure 1).^4a^ These limitations have led to the development of unnatural mimics of these α-amino acids, which are more similar in size and can be selectively embedded into peptides without altering structure.^5^ For example, 4-biphenyl-l-phenylalanine (**4**) was incorporated into dihydrofolate reductase to study conformational changes of inhibitor binding using Förster resonance energy-transfer (FRET) measurements.^7^ Tyrosine analogues with extended conjugation and improved photoluminescent properties such as bis-styrene **5** have been incorporated into cell-penetrating peptides and used for cell imaging.^8^ As tryptophan has the strongest fluorescent properties of the proteinogenic amino acids, many studies have focused on the modification of this α-amino acid.^9^ In particular, the reactivity of the C2-position of the indole ring has allowed the preparation of a wide range of analogues with extended conjugation (e.g., **6**).^10^ Several of these tryptophan mimics have been incorporated into peptides and used to image fungal infections and cancer cells.^10c,10e^

We have been interested in developing fluorescent α-amino acids with biaryl side chains as brighter, structural analogues of phenylalanine, tyrosine, and tryptophan.^11,12^ As well as the synthesis of pyrazole- and benzotriazole-derived α-amino acids, we recently reported the synthesis and photoluminescent properties of pyridine-derived α-amino acids **7** (Figure 1).^13^ These were prepared using a Lewis-acid-catalyzed hetero-Diels–Alder reaction of enone-derived α-amino acids with ethyl vinyl ether, followed by a Knoevenagel–Stobbe reaction to access the pyridine motif. Analysis of the photoluminescent properties of these α-amino acids revealed that a combination of electron-rich aryl substituents with the π-deficient pyridines resulted in charge-transfer-based fluorescence. Although several of the pyridine-derived α-amino acids displayed good quantum yields (0.18–0.46) and brightness, the main absorption bands were found at similar wavelengths to the fluorescent proteinogenic α-amino acids, thus restricting applications of these compounds. To overcome this limitation, we considered various structural changes that may result in fluorescent α-amino acids with red-shifted absorption and emission properties. To avoid increasing the size of the side chain resulting in α-amino acids significantly larger than proteinogenic analogues, we proposed that the incorporation of a more π-deficient heterocycle, such as a pyrimidine would enhance charge-transfer properties of the biaryl system, leading to a bathochromic shift of optical properties. Here, we report the synthesis of pyrimidine-derived α-amino acids (Figure 1) using an ytterbium-catalyzed heterocyclization reaction of ynone-derived α-amino acids with amidines as the key step. By tuning the fluorescent properties of these compounds through variation of the C2- and C4-substituents of the pyrimidine, we also demonstrate the most effective biaryl systems that generates charge-transfer-based fluorescent α-amino acids with red-shifted photoluminescent properties.

## Results and Discussion

**Scheme 1:**
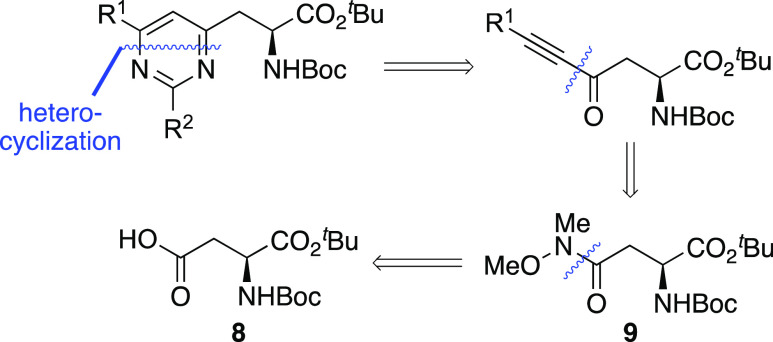
Proposed Synthesis of Pyrimidine-Derived α-Amino Acids

Our proposed synthesis of pyrimidine-derived α-amino acids involved three key disconnections (Scheme 1).^14^ Preparation of the pyrimidine ring and introduction of late-stage diversity would be achieved by heterocyclization of ynone-derived α-amino acids with amidines. The ynone-derived α-amino acids would be prepared by the chemoselective reaction of alkynyl lithium salts with Weinreb amide **9**, which would also allow the incorporation of various side chains. Weinreb amide **9** would be prepared under standard conditions from commercially available *N*-Boc l-aspartic acid *t*-butyl ester **8**.

**Scheme 2:**
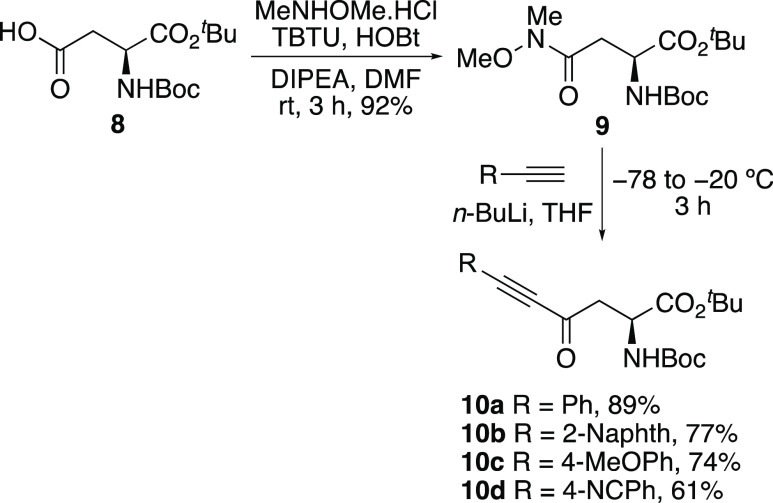
Two-Step Synthesis of Ynone-Derived α-Amino Acids **10a–d** Isolated yields are shown.

The first stage of the synthetic program focused on the scalable synthesis of ynone-derived α-amino acids. Renault and co-workers previously reported an efficient route to these compounds and thus, with some modifications, this was used for the preparation of ynones **10a**–**10d** (Scheme 2).^15^ Initially, commercially available *N*-Boc l-aspartic acid *t*-butyl ester **8** was treated with *N*,*O*-dimethyl hydroxylamine hydrochloride in the presence of TBTU and HOBt, which gave Weinreb amide **9** in 92% yield. The ynone-derived α-amino acids were then prepared by the reaction of Weinreb amide **9** with alkynyl lithium salts, generated by the deprotonation of aryl-substituted alkynes with *n*-butyl lithium. This gave ynones **10a**–**10d** cleanly, in 61–89% yield. Although the majority of ynones prepared possessed conjugated or electron-rich aryl side chains, which would lead to charge transfer with the π-deficient pyrimidine, an electron-deficient analogue bearing a 4-cyanophenyl group (**10d**) was also synthesized to examine the interaction of this with the pyrimidine ring.

In 1946, Bowden and Jones reported the synthesis of a pyrimidine by the reaction of an ynone with guanidine under basic conditions.^16^ More recently, similar procedures have been used for the synthesis of pyrimidines from ynones,^17^ including a study by Baldwin and co-workers that reported the synthesis of pyrimidine-derived α-amino acids from l-aspartic acid and l-glutamic acid derivatives.^18^ The majority of these procedures involved reaction of the ynone with an amidine by heating under reflux in the presence of sodium carbonate. Our initial studies investigated the reaction of phenyl-substituted ynone **10a** with benzamidine hydrochloride under similar conditions (Table 1, entry 1). Although this gave pyrimidine **11a** cleanly, the compound was isolated in only 23% yield. Attempts were then made to modify this approach. In addition, we wanted to avoid the combination of basic conditions and high temperatures (80 °C) and so a reaction at 50 °C and with a longer reaction time (24 h) was investigated (entry 2). This gave pyrimidine **11a** in an improved 42% yield. To avoid the use of water as a cosolvent, DMF was then investigated under the same conditions, but this gave no reaction (entry 3). In their work, Baldwin and co-workers highlighted that reaction of ynones with formamidine under these conditions gave low yields of the corresponding pyrimidine. This was a concern as we believed that pyrimidine-derived α-amino acids with no C2-substituent were likely to produce the most effective fluorophores. For this reason and the modest yields already observed for base-mediated heterocyclizations, we considered a different approach for the preparation of the pyrimidines. Bagley and co-workers showed that ynones could be activated with ytterbium salts for reaction with enamines and the subsequent synthesis of pyridine derivatives.^19^ Based on this, the use of ytterbium triflate as a Lewis acid catalyst for pyrimidine synthesis was investigated. An initial attempt involved the reaction of phenyl-substituted ynone **10a** with benzamidine hydrochloride in the presence of ytterbium triflate (10 mol %) (entry 4). Using THF as the solvent and potassium carbonate to neutralize the benzamidine salt, this generated pyrimidine **11a** cleanly and with an isolated yield of 69%.

**Table 1:**
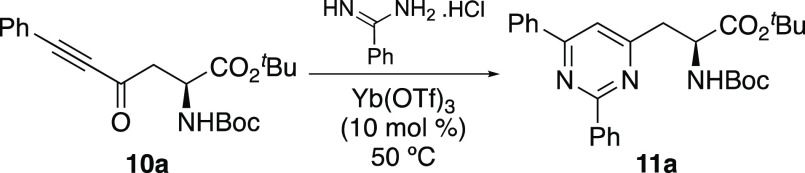
Optimization Studies for Pyrimidine Formation from Ynone **10a**^a^

entry	catalyst (mol %)	base	solvent	time (h)	yield (%)
1^b^		Na_2_CO_3_	MeCN/H_2_O	4	23
2		Na_2_CO_3_	MeCN/H_2_O	24	42
3		Na_2_CO_3_	DMF	18	0
4	10	K_2_CO_3_	THF	48	69

a Isolated yields are shown.

b Reaction done at 80 °C.

**Scheme 3:**
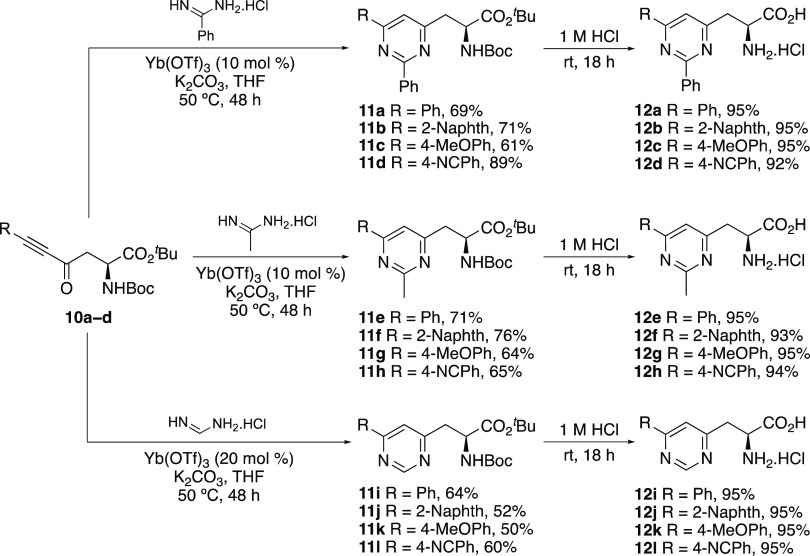
Synthesis of Pyrimidine-Derived α-Amino Acids **12a–l** Isolated yields are shown.

The scope of the ytterbium triflate catalyzed heterocyclization reaction of ynone-derived α-amino acids **10a**–**10d** with benzamidine, acetamidine, and formamidine was then explored (Scheme 3). The reaction of ynones **10a**–**10d** with benzamidine and acetamidine under the Lewis-acid-catalyzed conditions was found to be highly effective and gave the pyrimidine products in 61–89% yields.^20^ For the more challenging heterocyclization reaction with formamidine, the optimized conditions gave low yields. For example, reaction of *p*-methoxyphenyl-substituted ynone **10c** with formamidine hydrochloride and using ytterbium triflate (10 mol %) gave the corresponding pyrimidine **11k** in 19% yield. However, it was found that the use of higher catalyst loading (20 mol %) resulted in an improved 50% yield. This modification was also effective with the other ynones, allowing the synthesis of pyrimidines **11i**–**11l** in 50–64% yields. Having successfully synthesized a small library of pyrimidine-derived α-amino acids, the protecting groups were removed in the presence of 1 M hydrochloric acid under mild conditions, which gave parent amino acids **12a**–**12l** in excellent yields.

**Figure 2:**
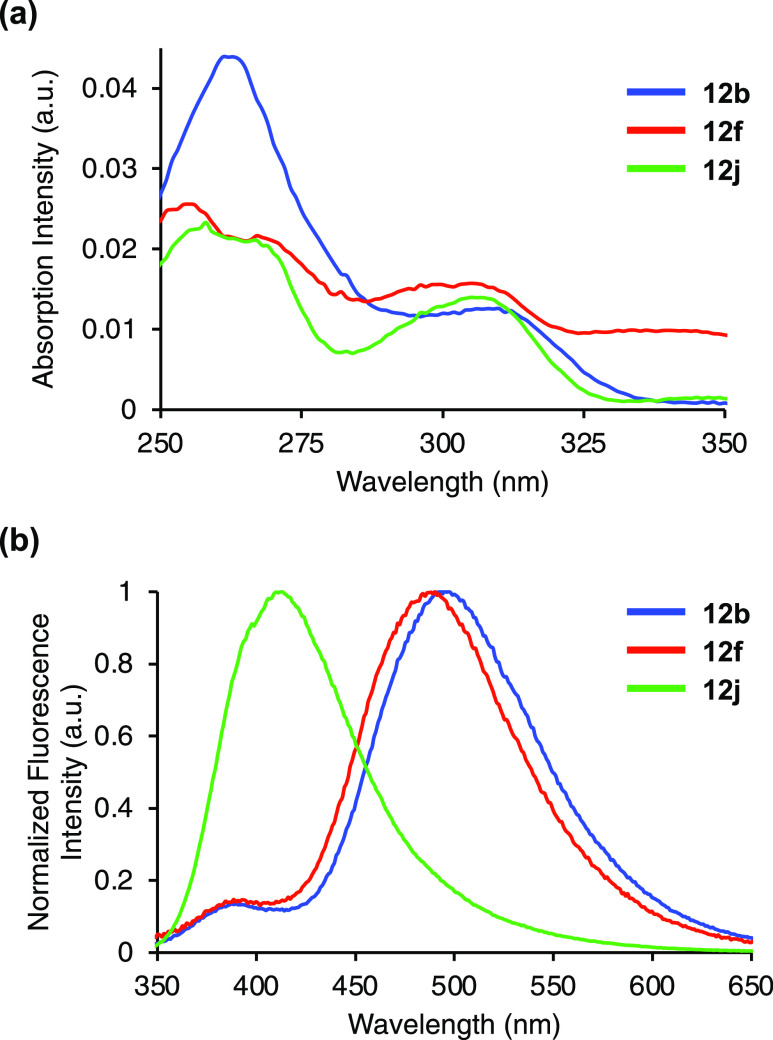
(a) Absorption spectra of **12b**, **12f**, and **12j**, recorded at 2 μM in methanol. (b) Emission spectra of **12b**, **12f**, and **12j**, recorded at 2 μM in methanol.

**Figure 3:**
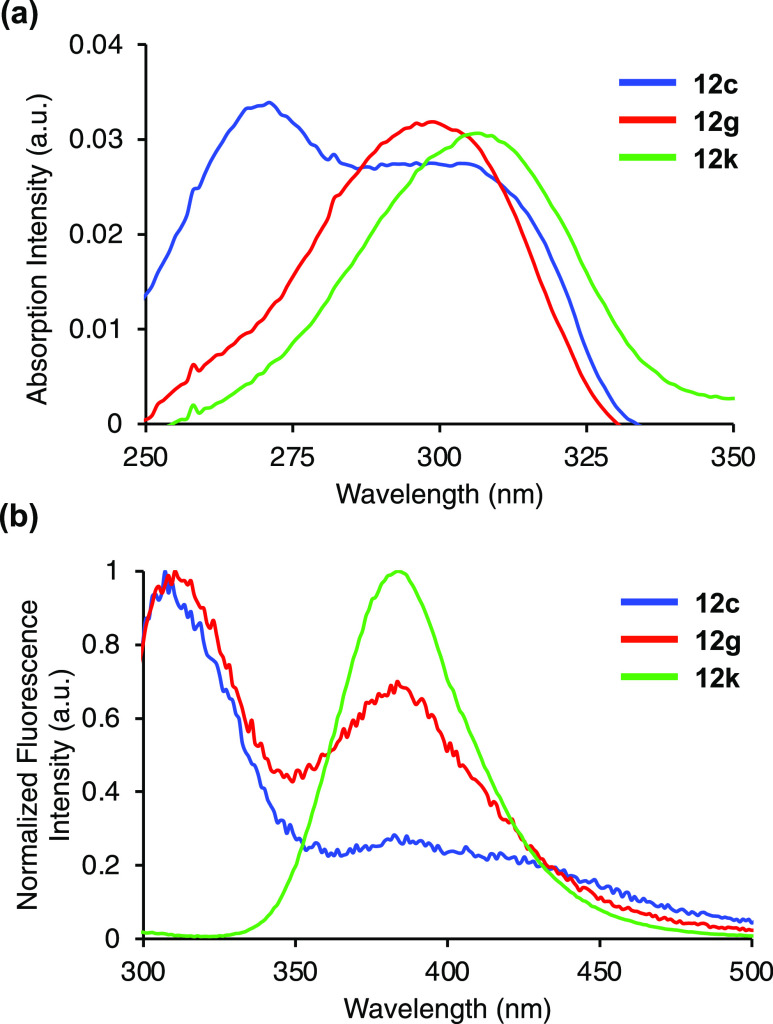
(a) Absorption spectra of **12c**, **12g**, and **12k**, recorded at 2 μM in methanol. (b) Emission spectra of **12c**, **12g**, and **12k**, recorded at 2 μM in methanol.

On synthesis of the pyrimidine-derived α-amino acids, the photoluminescent properties were measured for each compound. The ultraviolet–visible (UV–vis) absorption and photoluminescence spectra of the α-amino acids were recorded in methanol at a concentration of 2 μM. As expected, pyrimidines with weakly donating (Ph) or electron-withdrawing (4-NCPh) C4-substituents displayed weak fluorescence and low brightness (see the Supporting Information). The most interesting properties were found for pyrimidines with highly conjugated (naphthyl) and strongly electron-donating (4-MeOPh) C4-substituents (Table 2). These α-amino acids exhibited red-shifted absorption bands in comparison to proteinogenic α-amino acids **1**–**3** and the previously reported pyridine analogues,^13^ with fluorescence in the visible region. For the C4-naphthyl compounds (**12b**, **12f**, and **12j**), all three compounds showed absorption bands between 305 and 311 nm, possessed megaStokes shifts, and good quantum yields, resulting in the brightest series of α-amino acids (Table 2 and Figure 2). The main difference in this series was observed in the fluorescence spectra, in which α-amino acids **12b** and **12f** showed emission maxima between 490 and 500 nm, while a hypsochromic shift in emission to 421 nm was observed for C2-unsubstituted analogue **12j** (Figure 2b). For **12j**, we believe that the lack of a C2-substituent allows emission from a more planar locally excited state, while α-amino acids **12b** and **12f**, which have more distorted conformations due to C2-substituents, emit from twisted intramolecular charge-transfer excited states. For the *p*-methoxyphenyl series, the trend of emission maxima was found to be reversed. The C2-substituted compounds **12c** and **12g** displayed weak emission maxima at 314 nm, while **12k** with no C2-substituent showed a bathochromic shift in emission to 384 nm (Figure 3b). In this series, the C2-substituent obviously has a strong influence on the interaction between the C4-aryl group and the pyrimidine ring. While the C2-phenyl and methyl groups disrupt this interaction, no substituent at C2 allows strong interplay between the electron-rich *p*-methoxyphenyl ring and the π-deficient pyrimidine heterocycle, resulting in strong intramolecular charge-transfer emission. As well as strong fluorescence, α-amino acid **12k** displayed red-shifted absorption compared to the corresponding pyridine (283 nm),^13^ a large Stokes shift, as well as a good quantum yield (0.27) and brightness. Overall, these results provide insight into the relationship between the structure and photoluminescence properties of these α-amino acids and, in particular, the use of substituents to control biaryl conformation, leading to emission from either locally excited or twisted/planar intramolecular charge-transfer excited states.

**Table 2:** Photophysical Data of Pyrimidine-Derived α-Amino Acids

amino acid	λ_Abs_ (nm)^a^	ε (cm^–1^ M^–1^)	λ_Em_ (nm)^a^	Stokes shift (cm^–1^)	Φ_F_^b^	brightness (cm^–1^ M^–1^)
**12b**	311	24,000	497	12,033	0.12	2880
**12c**	305	12,700	314	940	0.003	38
**12f**	305	12,800	490	12,379	0.11	1408
**12g**	299	16,400	314, 381	1598, 7198	0.016	262
**12j**	310	10,400	421	8505	0.30	3120
**12k**	306	13,600	384	6638	0.27	3672

a Spectra were recorded at 2 μM in methanol.

b Quantum yields (Φ_F_) were determined in methanol using anthracene and l-tryptophan as standards.

**Figure 4:**
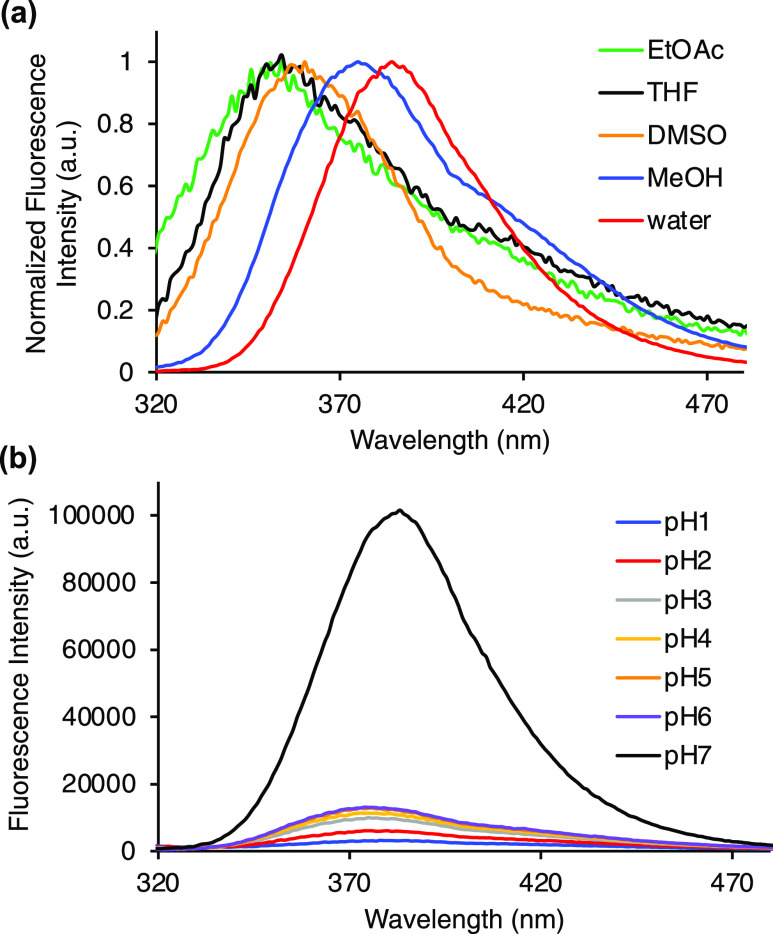
(a) Normalized fluorescence spectra of **12k** in various solvents. (b) Emission spectra of **12k** at various pH in methanol. All spectra were recorded using a concentration of 5 μM.

Although the naphthyl series of α-amino acids gave strong, red-shifted emission and good quantum yields, the *p*-methoxyphenyl pyrimidine-derived α-amino acid **12k** was found to be the brightest. For this reason, the properties of **12k** were further explored via solvatochromic and pH studies. Analysis of α-amino acid **12k** in a range of solvents produced similar absorption spectra, indicating that in the ground state, the absorbance is independent of solvent polarity (see the Supporting Information). In contrast, a bathochromic shift in emission maxima was observed with increasing solvent polarity (Figure 4a). For example, the emission maximum was found at 352 nm in ethyl acetate, while this shifted to 384 nm in water. The solvatochromism displayed by α-amino acid **12k** confirms the intramolecular charge-transfer character of the excited state, which is stabilized in more polar solvents. To determine the effect of pyrimidine ring protonation on the photophysical properties of α-amino acid **12k**, pH studies were conducted. A change of pH from 7 to 1, resulted in stronger absorbance around 300 nm and the formation of a second minor band at longer wavelength (∼360 nm) (see the Supporting Information). A significant change was also observed for the emission properties of α-amino acid **12k** (Figure 4b). While the position of the emission band is unchanged, the fluorescence is “turned off” following acidification. Protonation of substituted pyrimidines typically leads to a change in conformation, resulting in either a hypsochromic or bathochromic shift of emission bands.^21^ For α-amino acid **12k**, formation of a positively charged pyrimidine ring results in effective fluorescence quenching. These results suggest that α-amino acid **12k** may have potential as a fluorescent probe for biological applications, which involve a change of polarity or pH conditions.

## Conclusions

In conclusion, a small library of α-amino acids with pyrimidine side chains were prepared using a novel ytterbium-catalyzed heterocyclization reaction of ynone-derived α-amino acids with amidines as the key step. The pyrimidine heterocycle was selected as a strong π-deficient motif, which with conjugated or electron-rich aryl substituents would result in strong fluorescence. Analysis of the optical properties of these compounds proved this to be the case. Pyrimidines with no C2-substituent and with highly conjugating (**12j**) or electron-donating C4-substituents (**12k**) displayed red-shifted absorption bands (compared to the corresponding pyridine analogue),^13^ strong fluorescence emission in the visible region, large Stokes shifts, and good quantum yields (0.27–0.30). The intermolecular charge-transfer properties of the brightest α-amino acid **12k** were confirmed with a solvatochromic study, which showed a bathochromic shift of emission in more polar solvents. α-Amino acid **12k** was also found to be highly sensitive to pH, with protonation of the pyrimidine ring resulting in fluorescence quenching. This study has generated further insight into the relationship between structure, conformation, and photoluminescent properties of biaryl-derived α-amino acids such as **12k** that have the potential to act as fluorescent probes. Current work is investigating this potential for biological applications.

## Experimental Section

All reagents and starting materials were obtained from commercial sources and used as received. Reactions were performed open to air unless otherwise mentioned. All reactions performed at elevated temperatures were heated using an oil bath. Brine refers to a saturated aqueous solution of sodium chloride. Flash column chromatography was performed using silica gel 60 (40–63 μm). Aluminum-backed plates precoated with silica gel 60F_254_ were used for thin-layer chromatography and were visualized with a UV lamp or by staining with potassium permanganate, vanillin, or ninhydrin. ^1^H NMR spectra were recorded on a Bruker DPX 400 MHz spectrometer and data are reported as follows: chemical shift in ppm relative to the solvent as an internal standard (CHCl_3_, δ 7.26 ppm; CH_3_OH, δ 3.31 ppm), multiplicity (s = singlet, d = doublet, t = triplet, q = quartet, m = multiplet or overlap of nonequivalent resonances, integration). The abbreviation br s refers to broad singlet. ^13^C NMR spectra were recorded on an NMR spectrometer at 101 MHz and data are reported as follows: chemical shift in ppm relative to tetramethylsilane or the solvent as an internal standard (CDCl_3_, δ 77.2 ppm; CD_3_OD, δ 49.0 ppm). Infrared spectra were recorded using a Shimadzu IR Prestige-21 spectrometer; wavenumbers are indicated in cm^–1^. Mass spectra were recorded using electrospray techniques. HRMS spectra were recorded using Bruker micrOTOF-Q or Agilent 6546 LC/Q-TOF mass spectrometers. Melting points are uncorrected. Optical rotations were determined as solutions irradiating with the sodium D line (λ = 589 nm) using an Autopol V polarimeter. [α]_D_ values are given in units 10^–1^ deg cm^–1^ g^–1^. UV–vis spectra were recorded on a PerkinElmer Lambda 25 instrument. Fluorescence spectra were recorded on a Shimadzu RF-5301PC spectrofluorophotometer. Absorbance spectra were recorded with an integration time of 0.05 s and a band pass of 5 nm. Fluorescence spectra were recorded with excitation and emission band pass of 10 nm, an integration time of 0.1 or 2 s, and with detector accumulations set to 1. Quantum yield data were measured using anthracene and L-tryptophan as standard references.

### *tert*-Butyl (2*S*)-(*tert*-Butoxycarbonylamino)-4-[methoxy(methyl)amino]-4-oxobutanoate (**9**)^15^

A mixture of 1-*tert*-butyl (2*S*)-(*tert*-butoxycarbonylamino)-4-butan-1,4-dioic acid (**8**) (0.50 g, 1.76 mmol), *N*,*O*-dimethylhydroxyamine hydrochloride (0.24 g, 2.44 mmol), and *O*-(1*H*-benzotriazol-1-yl)-*N*,*N*,*N*′,*N*′-tetramethyluronium tetrafluoroborate (0.73 g, 1.93 mmol) was stirred in *N,N*′-dimethylformamide (3 mL) at 0 °C. Diisopropylethylamine (0.75 mL, 4.40 mmol) was then added dropwise. The mixture was stirred at room temperature for 3 h. An aqueous solution of 1 M sodium hydrogen sulfate (25 mL) was then added, and the mixture was extracted with ethyl acetate (3 × 20 mL). The combined organic layers were then washed with brine (3 × 20 mL), dried (MgSO_4_), and concentrated under vacuum. Purification by flash column chromatography eluting with 20% ethyl acetate in dichloromethane gave *tert*-butyl (2*S*)-(*tert*-butoxycarbonylamino)-4-[methoxy(methyl)amino]-4-oxobutanoate (**9**) as a colorless oil (0.59 g, 92%). [α]_D_^20^ −14.5 (*c* 0.6, MeOH) [lit.^15^ [α]_D_^25^ −12.3 (*c* 1.0, MeOH)]; ^1^H NMR (CDCl_3_, 400 MHz): δ 5.67 (d, *J* = 8.3 Hz, 1H), 4.47–4.43 (m, 1H), 3.18–3.12 (m, 4H), 3.69 (s, 3H), 2.87 (dd, *J* = 16.8, 2.7 Hz, 1H), 1.46 (s, 9H), 1.44 (s, 9H); ^13^C{^1^H} NMR (CDCl_3_, 101 MHz): δ 171.8, 170.5, 155.7, 81.7, 79.5, 61.2, 50.4, 34.7, 32.0, 28.3, 27.9; MS (ESI) *m*/*z* 355 (M + Na^+^, 100).

### *tert*-Butyl (2*S*)-(*tert*-Butoxycarbonylamino)-4-oxo-6-phenylhex-5-ynoate (**10a**)^15^

To a solution of phenylacetylene (0.20 mL, 1.9 mmol) in tetrahydrofuran (10 mL) at −78 °C was slowly added a solution of *n*-butyl lithium (2.5 M in hexane, 0.43 mL, 1.1 mmol). The mixture was stirred for 0.75 h and then added dropwise to a solution of *tert*-butyl (2*S*)-(*tert*-butoxycarbonylamino)-4-[methoxy(methyl)amino]-4-oxobutanoate (**9**) (0.12 g, 0.36 mmol) in tetrahydrofuran (25 mL) at −78 °C. The reaction mixture was stirred at −78 °C for 1 h and then at −20 °C for 2 h. An aqueous 1 M solution of dipotassium hydrogen phosphate (10 mL) was added, and the mixture was extracted with diethyl ether (3 × 30 mL). The combined organic layers were washed with brine (4 × 30 mL) and dried (MgSO_4_). After filtration, the solution was concentrated under vacuum. Purification by column chromatography eluting with 20% diethyl ether in petroleum gave *tert*-butyl (2*S*)-(*tert*-butoxycarbonylamino)-4-oxo-6-phenylhex-5-ynoate (**10a**) as a yellow oil (0.13 g, 89%). [α]_D_^20^ −7.5 (*c* 0.5, MeOH) [lit.^15^ [α]_D_^25^ −3.8 (*c* 1.0, MeOH)]; ^1^H NMR (CDCl_3_, 400 MHz): δ 7.62–7.55 (m, 2H), 7.50–7.43 (m, 1H), 7.42–7.36 (m, 2H), 5.44 (d, *J* = 8.1 Hz, 1H), 4.53–4.49 (m, 1H), 3.33 (dd, *J* = 17.9, 4.6 Hz, 1H), 3.18 (dd, *J* = 17.9, 4.6 Hz, 1H), 1.46 (s, 9H), 1.45 (s, 9H); ^13^C{^1^H} NMR (CDCl_3_, 101 MHz): δ 184.7, 169.8, 155.5, 133.2, 131.0, 128.7, 119.6, 92.0, 87.4, 82.5, 79.9, 50.2, 47.6, 28.3, 27.9; MS (ESI) *m*/*z* 396 (M + Na^+^, 100).

### *tert*-Butyl (2*S*)-(*tert*-Butoxycarbonylamino)-4-oxo-6-(2′-naphthyl)hex-5-ynoate (**10b**)

The reaction was carried out according to the above procedure for the synthesis of **10a** using 2-ethylnylnaphthalene (0.97 g, 6.4 mmol), *n*-butyl lithium (2.5 M in hexane, 1.5 mL, 3.8 mmol), and *tert*-butyl (2*S*)-(*tert*-butoxycarbonylamino)-4-[methoxy (methyl)amino]-4-oxobutanoate (**9**) (0.42 g, 1.3 mmol). Purification by flash column chromatography eluting with 20% diethyl ether in petroleum gave *tert*-butyl (2*S*)-(*tert*-butoxycarbonylamino)-4-oxo-6-(2′-naphthyl)hex-5-ynoate (**10b**) as a colorless oil (0.41 g, 77%). IR (neat) 3342, 2977, 2197, 1708, 1667, 1496, 1366, 1215, 1080, 746 cm^–1^; [α]_D_^24^ +9.0 (*c* 0.1, CHCl_3_); ^1^H NMR (CDCl_3_, 400 MHz): δ 8.15 (s, 1H), 7.85–7.80 (m, 3H), 7.58–7.50 (m, 3H), 5.49 (d, *J* = 8.6 Hz, 1H), 4.56 (dt, *J* = 8.6, 4.6 Hz, 1H), 3.37 (dd, *J* = 17.9, 4.6 Hz, 1H), 3.23 (dd, *J* = 17.9, 4.6 Hz, 1H), 1.48 (s, 9H), 1.46 (s, 9H); ^13^C{^1^H} NMR (CDCl_3_, 101 MHz): δ 184.7, 169.9, 155.5, 134.6, 134.0, 132.6, 128.5, 128.4, 128.2, 128.1, 127.9, 127.1, 116.8, 92.5, 87.7, 82.5, 79.9, 50.2, 47.6, 28.4, 27.9; MS (ESI) *m*/*z* 446 (M + Na^+^, 100); HRMS (ESI) *m*/*z*: [M + Na]^+^ Calcd for C_25_H_29_NO_5_Na 446.1938; found 446.1948.

### *tert*-Butyl (2*S*)-(*tert*-Butoxycarbonylamino)-4-oxo-6-(4′-methoxyphenyl)hex-5-ynoate (**10c**)

The reaction was carried out according to the above procedure for the synthesis of **10a** using 4-methoxyphenylacetylene (0.70 mL, 5.40 mmol), *n*-butyl lithium (2.5 M in hexane, 1.30 mL, 3.24 mmol), and *tert*-butyl (2*S*)-(*tert*-butoxycarbonylamino)-4-[methoxy(methyl)amino]-4-oxobutanoate (**9**) (0.35 g, 1.1 mmol). Purification by flash column chromatography eluting with 30% diethyl ether in petroleum gave *tert*-butyl (2*S*)-(*tert*-butoxycarbonylamino)-4-oxo-6-(4′-methoxyphenyl)hex-5-ynoate (**10c**) as a white solid (0.32 g, 74%). Mp 118–120 °C; IR (neat) 3376, 2978, 2361, 2193, 1710, 1665, 1509, 1367, 1253, 1153, 1086, 739 cm^–1^; [α]_D_^21^ +16.4 (*c* 0.1, CHCl_3_); ^1^H NMR (CDCl_3_, 400 MHz): δ 7.57–7.51 (m, 2H), 6.92–6.87 (m, 2H), 5.43 (d, *J* = 8.5 Hz, 1H), 4.50 (dt, *J* = 8.5, 4.5 Hz, 1H), 3.85 (s, 3H), 3.31 (dd, *J* = 17.8, 4.5 Hz, 1H), 3.16 (dd, *J* = 17.8, 4.5 Hz, 1H), 1.46 (s, 9H), 1.44 (s, 9H); ^13^C{^1^H} NMR (CDCl_3_, 101 MHz): δ 184.6, 169.9, 161.9, 155.5, 135.3, 114.4, 111.4, 93.3, 87.5, 82.4, 79.8, 55.4, 50.2, 47.4, 28.3, 27.9; MS (ESI) *m*/*z* 426 (M + Na^+^, 100); HRMS (ESI) *m*/*z*: [M + Na]^+^ Calcd for C_22_H_29_NO_6_Na 426.1887; found 426.1881.

### *tert*-Butyl (2*S*)-(*tert*-Butoxycarbonylamino)-4-oxo-6-(4′-cyanophenyl)hex-5-ynoate (**10d**)

The reaction was carried out according to the above procedure for the synthesis of **10a** using 4-cyanophenylacetylene (0.21 g, 1.7 mmol), *n*-butyl lithium (2.5 M in hexane, 0.40 mL, 1.0 mmol), and *tert*-butyl (2*S*)-(*tert*-butoxycarbonylamino)-4-[methoxy(methyl)amino]-4-oxobutanoate (**9**) (0.12 g, 0.33 mmol). Purification by flash column chromatography eluting with 30% diethyl ether in petroleum gave *tert*-butyl (2*S*)-(*tert*-butoxycarbonylamino)-4-oxo-6-(4′-cyanophenyl)hex-5-ynoate (**10d**) as a white solid (0.10 g, 61%). Mp 149–151 °C; IR (neat) 3391, 2978, 2366, 2207, 1710, 1678, 1500, 1367, 1252, 1150, 1086, 844, 736 cm^–1^; [α]_D_^25^ +9.2 (*c* 0.1, CHCl_3_); ^1^H NMR (CDCl_3_, 400 MHz): δ 7.71–7.66 (m, 4H), 5.39 (d, *J* = 8.2 Hz, 1H), 4.53 (dt, *J* = 8.2, 4.8 Hz, 1H), 3.31 (dd, *J* = 17.8, 4.8 Hz, 1H), 3.20 (dd, *J* = 17.8, 4.8 Hz, 1H), 1.46 (s, 9H), 1.45 (s, 9H); ^13^C{^1^H} NMR (CDCl_3_, 101 MHz): δ 184.2, 169.6, 155.4, 133.4, 132.3, 124.4, 117.8, 114.3, 89.6, 88.4, 82.8, 80.1, 50.2, 47.6, 28.3, 27.9; MS (ESI) *m/z* 421 (M + Na^+^, 100); HRMS (ESI) *m/z*: [M + Na]^+^ Calcd for C_22_H_26_N_2_O_5_Na 421.1734; found 421.1727.

### *tert-*Butyl (2*S*)-2-(*tert*-Butoxycarbonylamino)-3-(2′-phenyl-4′-phenylpyrimidin-6′-yl)propanoate (**11a**)

*tert*-Butyl (2*S*)-(*tert*-butoxycarbonylamino)-4-oxo-6-phenylhex-5-ynoate (**10a**) (0.046 g, 0.11 mmol) was dissolved in tetrahydrofuran (2 mL), followed by sequential addition of benzamidine hydrochloride (0.026 g, 0.17 mmol), potassium carbonate (0.018 g, 0.13 mmol), and ytterbium triflate (0.0070 g, 0.011 mmol). The mixture was heated to 50 °C for 48 h and then concentrated *in vacuo*. The residue was redissolved in dichloromethane (10 mL) and washed with a saturated solution of sodium hydrogen carbonate (5 mL) and then brine (5 mL). The organic layer was dried (MgSO_4_) and concentrated *in vacuo*. Purification by flash column chromatography eluting with 30% ethyl acetate in petroleum ether gave *tert-*butyl (2*S*)-2-(*tert*-butoxycarbonylamino)-3-(2′-phenyl-4′-phenylpyrimidin-6′-yl)propanoate (**11a**) (0.039 g, 69%) as a yellow solid. Mp 95–100 °C; IR (neat) 3429, 2977, 1706, 1583, 1501, 1364, 1250, 1149, 1031, 837, 729 cm^–1^; [α]_D_^18^ +26.0 (*c* 0.1, CHCl_3_); ^1^H NMR (CDCl_3_, 400 MHz): δ 8.61–8.57 (m, 2H), 8.23–8.19 (m, 2H), 7.53–7.49 (m, 6H), 7.47 (s, 1H), 5.94 (d, *J* = 8.6 Hz, 1H), 4.73 (dt, *J* = 8.6, 5.1 Hz, 1H), 3.49 (dd, *J* = 15.7, 5.1 Hz, 1H), 3.36 (dd, *J* = 15.7, 5.1 Hz, 1H), 1.44 (s, 9H), 1.34 (s, 9H); ^13^C{^1^H} NMR (CDCl_3_, 101 MHz): δ 170.7, 166.9, 164.03, 163.97, 155.6, 137.7, 137.0, 130.9, 130.7, 128.9, 128.52, 128.46, 127.2, 114.2, 81.9, 79.7, 52.5, 39.3, 28.4, 28.0; MS (ESI) *m*/*z* 498 (M + Na^+^, 100); HRMS (ESI) *m*/*z*: [M + Na]^+^ Calcd for C_28_H_33_N_3_O_4_Na 498.2363; found 498.2355.

### *tert-*Butyl (2*S*)-2-(*tert*-Butoxycarbonylamino)-3-[4′-(2″-naphthyl)-2′-phenylpyrimidin-6′-yl]propanoate (**11b**)

*tert-*Butyl (2*S*)-2-(*tert*-butoxycarbonylamino)-3-[4′-(2″-naphthyl)-2′-phenylpyrimidin-6′-yl]propanoate (**11b**) was synthesized as described for **11a** using *tert*-butyl (2*S*)-(*tert*-butoxycarbonylamino)-4-oxo-6-(2′-naphthyl)hex-5-ynoate (**10b**) (0.076 g, 0.18 mmol), benzamidine hydrochloride (0.042 g, 0.27 mmol), potassium carbonate (0.030 g, 0.22 mmol), and ytterbium triflate (0.010 g, 0.018 mmol). Purification by flash column chromatography eluting with 30% ethyl acetate in petroleum ether gave *tert-*butyl (2*S*)-2-(*tert*-butoxycarbonylamino)-3-[4′-(2″-naphthyl)-2′-phenylpyrimidin-6′-yl]propanoate (**11b**) (0.067 g, 71%) as a white solid. Mp 130–133 °C; IR (neat) 3366, 2978, 1713, 1571, 1537, 1495, 1367, 1153, 1058, 760, 698 cm^–1^; [α]_D_^24^ +10.0 (*c* 0.1, CHCl_3_); ^1^H NMR (CDCl_3_, 400 MHz): δ 8.72 (br s, 1H), 8.66–8.62 (m, 2H), 8.33 (dd, *J* = 8.6, 1.7 Hz, 1H), 8.03–7.90 (m, 3H), 7.63 (s, 1H), 7.58–7.50 (m, 5H), 5.95 (d, *J* = 8.6 Hz, 1H), 4.75 (dt, *J* = 8.6, 5.2 Hz, 1H), 3.53 (dd, *J* = 15.7, 5.2 Hz, 1H), 3.40 (dd, *J* = 15.7, 5.2 Hz, 1H), 1.44 (s, 9H), 1.35 (s, 9H); ^13^C{^1^H} NMR (CDCl_3_, 101 MHz): δ 170.7, 163.9, 137.7, 134.7, 134.3, 133.3, 130.8, 129.0, 128.7, 128.6, 128.5, 127.8, 127.5, 127.4, 126.6, 124.1, 116.8, 114.4, 82.0, 79.7, 52.5, 39.4, 28.4, 28.0; MS (ESI) *m*/*z* 548 (M + Na^+^, 100); HRMS (ESI) *m*/*z*: [M + Na]^+^ Calcd for C_32_H_35_N_3_O_4_Na 548.2520; found 548.2520.

### *tert-*Butyl (2*S*)-2-(*tert*-Butoxycarbonylamino)-3-[4′-(4″-methoxyphenyl)-2′-phenylpyrimidin-6′-yl]propanoate (**11c**)

*tert-*Butyl (2*S*)-2-(*tert*-butoxycarbonylamino)-3-[4′-(4″-methoxyphenyl)-2′-phenylpyrimidin-6′-yl]propanoate (**11c**) was synthesized as described for **11a** using *tert*-butyl (2*S*)-(*tert*-butoxycarbonylamino)-4-oxo-6-(4′-methoxyphenyl)hex-5-ynoate (**10c**) (0.44 g, 1.2 mmol), benzamidine hydrochloride (0.29 g, 1.8 mmol), potassium carbonate (0.20 g, 1.5 mmol), and ytterbium triflate (0.0070 g, 0.012 mmol). Purification by flash column chromatography eluting with 30% ethyl acetate in petroleum ether gave *tert-*butyl (2*S*)-2-(*tert*-butoxycarbonylamino)-3-[4′-(4″-methoxyphenyl)-2′-phenylpyrimidin-6′-yl]propanoate (**11c**) (0.31 g, 61%) as a white solid. Mp 205–210 °C; IR (neat) 3376, 2982, 1706, 1573, 1533, 1368, 1217, 1172, 763 cm^–1^; [α]_D_^20^ +24.5 (*c* 0.1, CHCl_3_); ^1^H NMR (CDCl_3_, 400 MHz): δ 8.59–8.57 (m, 2H), 8.20–8.18 (m, 2H), 7.54–7.48 (m, 3H), 7.40 (s, 1H), 7.04–7.02 (m, 2H), 5.98 (d, *J* = 8.1 Hz, 1H), 4.72–4.68 (m, 1H), 3.89 (s, 3H), 3.46 (dd, *J* = 15.6, 4.7 Hz, 1H), 3.33 (dd, *J* = 15.6, 4.5 Hz, 1H), 1.44 (s, 9H), 1.34 (s, 9H); ^13^C{^1^H} NMR (CDCl_3_, 101 MHz): δ 170.7, 166.5, 163.9, 163.5, 162.0, 155.6, 137.8, 130.6, 129.4, 128.8, 128.5, 128.4, 114.3, 113.3, 81.9, 79.6, 55.4, 52.5, 39.2, 28.4, 28.0; MS (ESI) *m*/*z* 506 (M + H^+^, 100); HRMS (ESI) *m*/*z*: [M + H]^+^ Calcd for C_29_H_35_N_3_O_5_H 506.2649; found 506.2647.

### *tert-*Butyl (2*S*)-2-(*tert*-Butoxycarbonylamino)-3-[4′-(4″-cyanophenyl)-2′-phenylpyrimidin-6′-yl]propanoate (**11d**)

*tert-*Butyl (2*S*)-2-(*tert*-butoxycarbonylamino)-3-[4′-(4″-cyanophenyl)-2′-phenylpyrimidin-6′-yl]propanoate (**11d**) was synthesized as described for **11a** using *tert*-butyl (2*S*)-(*tert*-butoxycarbonylamino)-4-oxo-6-(4′-cyanophenyl)hex-5-ynoate (**10d**) (0.10 g, 0.25 mmol), benzamidine hydrochloride (0.059 g, 0.37 mmol), potassium carbonate (0.041 g, 0.30 mmol), and ytterbium triflate (0.015 g, 0.025 mmol). Purification by flash column chromatography eluting with 30% ethyl acetate in petroleum ether gave *tert-*butyl (2*S*)-2-(*tert*-butoxycarbonylamino)-3-[4′-(4″-cyanophenyl)-2′-phenylpyrimidin-6′-yl]propanoate (**11d**) (0.11 g, 89%) as a yellow oil. IR (neat) 3425, 2978, 2229, 1705, 1569, 1533, 1367, 1150, 732, 695 cm^–1^; [α]_D_^24^ +30.2 (*c* 0.1, CHCl_3_); ^1^H NMR (CDCl_3_, 400 MHz): δ 8.60–8.55 (m, 2H), 8.32 (d, *J* = 8.4 Hz, 2H), 7.83 (d, *J* = 8.4 Hz, 2H), 7.55–7.51 (m, 4H), 5.80 (d, *J* = 8.4 Hz, 1H), 4.74 (dt, *J* = 8.4, 5.2 Hz, 1H), 3.51 (dd, *J* = 15.9, 5.2 Hz, 1H), 3.41 (dd, *J* = 15.9, 5.2 Hz, 1H), 1.43 (s, 9H), 1.36 (s, 9H); ^13^C{^1^H} NMR (CDCl_3_, 101 MHz): δ 170.6, 167.8, 164.4, 161.8, 155.5, 141.2, 137.2, 132.7, 131.1, 128.6, 128.5, 127.8, 118.4, 114.6, 114.3, 82.1, 79.9, 52.3, 39.5, 28.3, 28.0; MS (ESI) *m*/*z* 523 (M + Na^+^, 100); HRMS (ESI) *m*/*z*: [M + Na]^+^ Calcd for C_29_H_32_N_4_O_4_Na 523.2316; found 523.2316.

### *tert-*Butyl (2*S*)-2-(*tert*-Butoxycarbonylamino)-[3-(2′-methyl-4′-phenylpyrimidin-6′-yl)](propanoate) (**11e**)

*tert*-Butyl (2*S*)-(*tert*-butoxycarbonylamino)-4-oxo-6-phenylhex-5-ynoate (**10a**) (0.34 g, 0.91 mmol) was dissolved in tetrahydrofuran (30 mL), followed by sequential addition of acetamidine hydrochloride (0.13 g, 1.4 mmol), potassium carbonate (0.30 g, 2.2 mmol), and ytterbium triflate (0.056 g, 0.091 mmol). The mixture was heated to 50 °C for 48 h and was concentrated *in vacuo*. The residue was redissolved in dichloromethane (30 mL), washed with a saturated solution of sodium hydrogen carbonate (20 mL), brine (20 mL), dried (MgSO_4_), and concentrated *in vacuo*. Purification by flash column chromatography eluting with 30% ethyl acetate in petroleum ether gave *tert-*butyl (2*S*)-2-(*tert*-butoxycarbonylamino)-[3-(2′-methyl-4′-phenylpyrimidin-6′-yl)(propanoate)] (**11e**) (0.27 g, 71%) as a white solid. Mp 98–103 °C; IR (neat) 3665, 2979, 1705, 1580, 1541, 1366, 1149, 1055, 752, 693 cm^–1^; [α]_D_^25^ +16.4 (*c* 0.1, CHCl_3_); ^1^H NMR (CDCl_3_, 400 MHz): δ 8.07–8.03 (m, 2H), 7.51–7.47 (m, 3H), 7.41 (s, 1H), 5.65 (d, *J* = 8.1 Hz, 1H), 4.67–4.62 (m, 1H), 3.33 (dd, *J* = 15.1, 5.7 Hz, 1H), 3.27 (dd, *J* = 15.1, 5.2 Hz, 1H), 2.76 (s, 3H), 1.42 (s, 9H), 1.40 (s, 9H); ^13^C{^1^H} NMR (CDCl_3_, 101 MHz): δ 170.4, 167.7, 166.4, 164.3, 155.4, 136.9, 130.8, 128.9, 127.2, 113.8, 82.0, 79.7, 52.8, 39.4, 28.3, 27.9, 26.0; MS (ESI) *m*/*z* 436 (M + Na^+^, 100); HRMS (ESI) *m/z*: [M + Na]^+^ Calcd for C_23_H_31_N_3_O_4_Na 436.2207; found 436.2208.

### *tert-*Butyl (2*S*)-2-(*tert*-Butoxycarbonylamino)-[3-(2′-methyl-4′-phenylpyrimidin-6′-yl)](propanoate) (**11e**)—Gram-Scale Reaction^20^

The reaction was performed as described above using *tert*-butyl (2*S*)-(*tert*-butoxycarbonylamino)-4-oxo-6-phenylhex-5-ynoate (**10a**) (1.0 g, 2.7 mmol), acetamidine hydrochloride (0.39 g, 4.2 mmol), potassium carbonate (0.90 g, 6.6 mmol), and ytterbium triflate (0.17 g, 0.27 mmol). Purification by flash column chromatography eluting with 30% ethyl acetate in petroleum ether gave *tert-*butyl (2*S*)-2-(*tert*-butoxycarbonylamino)-[3-(2′-methyl-4′-phenylpyrimidin-6′-yl)(propanoate)] (**11e**) (0.80 g, 69%) as a white solid. Spectroscopic data are as described above.

### *tert-*Butyl (2*S*)-2-(*tert*-Butoxycarbonylamino)-3-[2′-methyl-4′-(2″-naphthyl)pyrimidin-6′-yl]propanoate (**11f**)

*tert-*Butyl (2*S*)-2-(*tert*-butoxycarbonylamino)-3-[2′-methyl-4′-(2″-naphthyl)pyrimidin-6′-yl]propanoate (**11f**) was synthesized as described for **11e** using *tert*-butyl (2*S*)-(*tert*-butoxycarbonylamino)-4-oxo-6-(2′-naphthyl)hex-5-ynoate (**10b**) (0.17 g, 0.39 mmol), acetamidine hydrochloride (0.092 g, 0.59 mmol), potassium carbonate (0.13 g, 0.94 mmol), and ytterbium triflate (0.024 g, 0.039 mmol). Purification by flash column chromatography eluting with 30% ethyl acetate in petroleum ether gave *tert-*butyl (2*S*)-2-(*tert*-butoxycarbonylamino)-3-[2′-methyl-4′-(2″-naphthyl)pyrimidin-6′-yl]propanoate (**11f**) (0.14 g, 76%) as a colorless oil. IR (neat) 3361, 2980, 1716, 1587, 1541, 1364, 1254, 1160, 1024, 758 cm^–1^; [α]_D_^20^ +20.0 (*c* 0.1, CHCl_3_); ^1^H NMR (CDCl_3_, 400 MHz): δ 8.61 (br s, 1H), 8.16 (d, *J* = 8.5 Hz, 1H), 7.98–7.87 (m, 3H), 7.61–7.52 (m, 3H), 5.69 (d, *J* = 8.0 Hz, 1H), 4.71–4.65 (m, 1H), 3.43–3.31 (m, 2H), 2.81 (s, 3H), 1.42 (br s, 18H); ^13^C{^1^H} NMR (CDCl_3_, 101 MHz): δ 170.4, 167.5, 166.2, 164.3, 155.5, 134.6, 134.0, 133.2, 129.1, 128.8, 127.8, 127.5, 126.6, 124.0, 114.0, 82.1, 79.8, 52.8, 39.3, 28.3, 27.9, 25.9; MS (ESI) *m*/*z* 464 (M + H^+^, 100); HRMS (ESI) *m/z*: [M + H]^+^ Calcd for C_27_H_33_N_3_O_4_H 464.2544; found 464.2555.

### *tert-*Butyl (2*S*)-2-(*tert*-Butoxycarbonylamino)-3-[4′-(4″-methoxyphenyl)-2′-methylpyrimidin-6′-yl]propanoate (**11g**)

*tert-*Butyl (2*S*)-2-(*tert*-butoxycarbonylamino)-3-[4′-(4″-methoxyphenyl)-2′-methylpyrimidin-6′-yl]propanoate (**11g**) was synthesized as described for **11e** using *tert*-butyl (2*S*)-(*tert*-butoxycarbonylamino)-4-oxo-6-(4′-methoxyphenyl)hex-5-ynoate (**10c**) (0.093 g, 0.23 mmol), acetamidine hydrochloride (0.033 g, 0.34 mmol), potassium carbonate (0.076 g, 0.55 mmol), and ytterbium triflate (0.014 g, 0.023 mmol). Purification by flash column chromatography eluting with 30% ethyl acetate in petroleum ether gave *tert-*butyl (2*S*)-2-(*tert*-butoxycarbonylamino)-3-[4′-(4″-methoxyphenyl)-2′-methylpyrimidin-6′-yl]propanoate (**11g**) (0.065 g, 64%) as a white solid. Mp 110–114 °C; IR (neat) 3372, 2976, 1708, 1584, 1516, 1370, 1254, 1159, 840, 731 cm^–1^; [α]_D_^19^ +15.0 (*c* 0.1, CHCl_3_); ^1^H NMR (CDCl_3_, 400 MHz): δ 8.03 (d, *J* = 8.8 Hz, 2H), 7.32 (s, 1H), 7.01–6.97 (m, 2H), 5.65 (d, *J* = 8.1 Hz, 1H), 4.62 (dt, *J* = 8.1, 5.5 Hz, 1H), 3.87 (s, 3H), 3.29 (dd, *J* = 15.0, 5.5 Hz, 1H), 3.21 (dd, *J* = 15.0, 5.5 Hz, 1H), 2.71 (s, 3H), 1.42 (s, 9H), 1.39 (s, 9H); ^13^C{^1^H} NMR (CDCl_3_, 101 MHz): δ 170.5, 167.7, 166.2, 163.6, 161.9, 155.4, 129.4, 128.7, 114.3, 112.8, 81.9, 79.7, 55.4, 52.8, 39.5, 28.3, 27.9, 26.2; MS (ESI) *m*/*z* 444 (M + H^+^, 100); HRMS (ESI) *m*/*z*: [M + H]^+^ Calcd for C_24_H_33_N_3_O_5_H 444.2493; found 444.2497.

### *tert-*Butyl (2*S*)-2-(*tert*-Butoxycarbonylamino)-3-[4′-(4″-cyanophenyl)-2′-methylpyrimidin-6′-yl]propanoate (**11h**)

*tert-*Butyl (2*S*)-2-(*tert*-butoxycarbonylamino)-3-[4′-(4″-cyanophenyl)-2′-methylpyrimidin-6′-yl]propanoate (**11h**) was synthesized as described for **11e** using *tert*-butyl (2*S*)-(*tert*-butoxycarbonylamino)-4-oxo-6-(4′-cyanophenyl)hex-5-ynoate (**10d**) (0.084 g, 0.21 mmol), acetamidine hydrochloride (0.030 g, 0.32 mmol), potassium carbonate (0.070 g, 0.50 mmol), and ytterbium triflate (0.013 g, 0.021 mmol). Purification by flash column chromatography eluting with 30% ethyl acetate in petroleum ether gave *tert-*butyl (2*S*)-2-(*tert*-butoxycarbonylamino)-3-[4′-(4″-cyanophenyl)-2′-methylpyrimidin-6′-yl]propanoate (**11h**) (0.065 g, 65%) as a colorless oil. IR (neat) 3393, 2885, 2237, 1744, 1592, 1501, 1450, 1247, 1033, 661 cm^–1^; [α]_D_^24^ +30.0 (*c* 0.1, CHCl_3_); ^1^H NMR (CDCl_3_, 400 MHz): δ 8.18 (d, *J* = 8.0 Hz, 2H), 7.81–7.77 (m, 2H), 7.44 (s, 1H), 5.54 (d, *J* = 8.1 Hz, 1H), 4.67–4.62 (m, 1H), 3.37–3.26 (m, 2H), 2.76 (s, 3H), 1.41 (br s, 18H); ^13^C{^1^H} NMR (CDCl_3_, 101 MHz): δ 170.3, 168.3, 167.4, 161.9, 155.4, 141.2, 132.6, 127.8, 118.4, 114.1, 82.2, 79.8, 52.7, 39.7, 28.3, 27.9, 26.1; MS (ESI) *m/z* 439 (M + H^+^, 100); HRMS (ESI) *m*/*z*: [M + H]^+^ Calcd for C_24_H_30_N_4_O_4_H 439.2340; found 439.2341.

### *tert-*Butyl (2*S*)-2-(*tert*-Butoxycarbonylamino)-3-(4′-phenylpyrimidin-6′-yl)propanoate (**11i**)

*tert*-Butyl (2*S*)-(*tert*-butoxycarbonylamino)-4-oxo-6-phenylhex-5-ynoate (**10a**) (0.083 g, 0.22 mmol) was dissolved in tetrahydrofuran (15 mL), followed by sequential addition of formamidine hydrochloride (0.18 g, 2.20 mmol), potassium carbonate (0.61 g, 4.40 mmol), and ytterbium triflate (0.027 g, 0.044 mmol). The mixture was heated to 50 °C for 48 h and was concentrated *in vacuo*. The residue was redissolved in dichloromethane (20 mL), washed with a saturated solution of sodium hydrogen carbonate (20 mL), brine (20 mL), dried (MgSO_4_), and concentrated *in vacuo*. Purification by flash column chromatography eluting with 30% ethyl acetate in petroleum ether gave *tert-*butyl (2*S*)-2-(*tert*-butoxycarbonylamino)-3-(4′-phenylpyrimidin-6′-yl)propanoate (**11i**) (0.053 g, 64%) as a colorless oil. IR (neat) 3668, 2977, 1713, 1587, 1512, 1368, 1254, 1156, 1024, 840, 747 cm^–1^; [α]_D_^19^ +40.0 (*c* 0.1, CHCl_3_); ^1^H NMR (CDCl_3_, 400 MHz): δ 9.15 (s, 1H), 8.09–8.05 (m, 2H), 7.60 (s, 1H), 7.53–7.49 (m, 3H), 5.69 (d, *J* = 8.5 Hz, 1H), 4.69–4.64 (m, 1H), 3.36 (dd, *J* = 15.2, 5.7 Hz, 1H), 3.29 (dd, *J* = 15.2, 5.0 Hz, 1H), 1.42 (s, 9H), 1.39 (s, 9H); ^13^C{^1^H} NMR (CDCl_3_, 101 MHz): δ 170.4, 166.8, 163.9, 158.5, 155.4, 136.6, 131.0, 129.0, 127.1, 116.8, 82.2, 79.8, 52.8, 39.7, 28.3, 27.9; MS (ESI) *m*/*z* 400 (M + H^+^, 100): HRMS (ESI) *m*/*z*: [M + H]^+^ Calcd for C_22_H_29_N_3_O_4_H 400.2231; found 400.2229.

### *tert-*Butyl (2*S*)-2-(*tert*-Butoxycarbonylamino)-3-[4′-(2″-naphthyl)pyrimidin-6′-yl]propanoate (**11j**)

*tert-*Butyl (2*S*)-2-(*tert*-butoxycarbonylamino)-3-[4′-(2″-naphthyl)pyrimidin-6′-yl]propanoate (**11j**) was synthesized as described for **11i** using *tert*-butyl (2*S*)-(*tert*-butoxycarbonylamino)-4-oxo-6-(2′-naphthyl)hex-5-ynoate (**10b**) (0.050 g, 0.12 mmol), formamidine hydrochloride (0.097 g, 1.20 mmol), potassium carbonate (0.33 g, 2.40 mmol), and ytterbium triflate (0.015 g, 0.024 mmol). Purification by flash column chromatography eluting with 30% ethyl acetate in petroleum ether gave *tert-*butyl (2*S*)-2-(*tert*-butoxycarbonylamino)-3-[4′-(2″-naphthyl)pyrimidin-6′-yl]propanoate (**11j**) (0.028 g, 52%) as a white solid. Mp 162–165 °C; IR (neat) 3430, 2979, 1715, 1613, 1497, 1366, 1152, 752, 694 cm^–1^; [α]_D_^19^ +34.0 (*c* 0.1, CHCl_3_); ^1^H NMR (CDCl_3_, 400 MHz): δ 9.20 (s, 1H), 8.61 (br s, 1H), 8.15 (dd, *J* = 8.6, 1.5 Hz, 1H), 7.99–7.95 (m, 2H), 7.92–7.87 (m, 1H), 7.75 (s, 1H), 7.59–7.53 (m, 2H), 5.72 (d, *J* = 8.2 Hz, 1H), 4.72–4.67 (m, 1H), 3.40 (dd, *J* = 15.1, 5.9 Hz, 1H), 3.33 (dd, *J* = 15.1, 5.1 Hz, 1H), 1.43 (s, 9H), 1.40 (s, 9H); ^13^C{^1^H} NMR (CDCl_3_, 101 MHz): δ 170.5, 166.9, 163.8, 158.5, 155.5, 134.6, 133.7, 133.3, 129.0, 128.84, 128.75, 127.5, 126.7, 123.8, 116.9, 82.2, 79.9, 52.8, 39.7, 28.3, 27.9; MS (ESI) *m*/*z* 448 ([M – H]^−^, 100); HRMS (ESI) *m*/*z*: [M – H]^−^ Calcd for C_26_H_30_N_3_O_4_ 448.2242; found 448.2230.

### *tert-*Butyl (2*S*)-2-(*tert*-Butoxycarbonylamino)-3-[4′-(4″-methoxyphenyl)pyrimidin-6′-yl]propanoate (**11k**)

*tert-*Butyl (2*S*)-2-(*tert*-butoxycarbonylamino)-3-[4′-(4″-methoxyphenyl)pyrimidin-6′-yl]propanoate (**11k**) was synthesized as described for **11i** using *tert*-butyl (2*S*)-(*tert*-butoxycarbonylamino)-4-oxo-6-(4′-methoxyphenyl)hex-5-ynoate (**10c**) (0.099 g, 0.25 mmol), formamidine hydrochloride (0.200 g, 2.50 mmol), potassium carbonate (0.69 g, 5.00 mmol), and ytterbium triflate (0.031 g, 0.050 mmol). Purification by flash column chromatography eluting with 30% ethyl acetate in petroleum ether gave *tert-*butyl (2*S*)-2-(*tert*-butoxycarbonylamino)-3-[4′-(4″-methoxyphenyl)pyrimidin-6′-yl]propanoate (**11k**) (0.054 g, 50%) as a colorless oil. IR (neat) 3368, 2979, 1714, 1592, 1529, 1365, 1254, 1150, 1025 cm^–1^; [α]_D_^18^ +19.0 (*c* 0.1, CHCl_3_); ^1^H NMR (CDCl_3_, 400 MHz): δ 9.08 (s, 1H), 8.07–8.03 (m, 2H), 7.53 (s, 1H), 7.03–7.00 (m, 2H), 5.71 (d, *J* = 8.2 Hz, 1H), 4.65 (dt, *J* = 8.2, 5.6 Hz, 1H), 3.88 (s, 3H), 3.33 (dd, *J* = 15.1, 5.6 Hz, 1H), 3.25 (dd, *J* = 15.1, 5.6 Hz, 1H), 1.42 (s, 9H), 1.38 (s, 9H); ^13^C{^1^H} NMR (CDCl_3_, 101 MHz): δ 170.5, 166.4, 163.4, 162.1, 158.4, 155.5, 128.9, 128.7, 115.8, 114.4, 82.1, 79.8, 55.4, 52.8, 39.6, 28.3, 27.9; MS (ESI) *m*/*z* 430 (M + H^+^, 100); HRMS (ESI) *m*/*z*: [M + H]^+^ Calcd for C_23_H_31_N_3_O_5_H 430.2336; found 430.2339.

### *tert-*Butyl (2*S*)-2-(*tert*-Butoxycarbonylamino)-3-[4′-(4″-cyanophenyl)pyrimidin-6′-yl]propanoate (**11l**)

*tert-*Butyl (2*S*)-2-(*tert*-butoxycarbonylamino)-3-[4′-(4″-cyanophenyl)pyrimidin-6′-yl]propanoate (**11l**) was synthesized as described for **11i** using *tert*-butyl (2*S*)-(*tert*-butoxycarbonylamino)-4-oxo-6-(4′-cyanophenyl)hex-5-ynoate (**10d**) (0.053 g, 0.13 mmol), formamidine hydrochloride (0.024 g, 1.30 mmol), potassium carbonate (0.036 g, 2.60 mmol), and ytterbium triflate (0.016 g, 0.026 mmol). Purification by flash column chromatography eluting with 30% ethyl acetate in petroleum ether gave *tert-*butyl (2*S*)-2-(*tert*-butoxycarbonylamino)-3-[4′-(4″-cyanophenyl)pyrimidin-6′-yl]propanoate (**11l**) (0.032 g, 60%) as a colorless oil. IR (neat) 3436, 2979, 2230, 1710, 1588, 1499, 1367, 1253, 1153, 1024, 903, 725 cm^–1^; [α]_D_^20^ +20.0 (*c* 0.1, CHCl_3_); ^1^H NMR (CDCl_3_, 400 MHz): δ 9.19 (s, 1H), 8.20 (d, *J* = 8.5 Hz, 2H), 7.82–7.79 (m, 2H), 7.65 (s, 1H), 5.61 (d, *J* = 8.0 Hz, 1H), 4.70–4.65 (m, 1H), 3.41–3.31 (m, 2H), 1.41 (s, 9H), 1.40 (s, 9H); ^13^C{^1^H} NMR (CDCl_3_, 101 MHz): δ 170.3, 167.7, 161.7, 158.7, 155.4, 140.7, 132.8, 127.7, 118.3, 117.2, 114.5, 82.4, 79.9, 52.7, 39.9, 28.3, 27.9; MS (ESI) *m*/*z* 425 (M + H^+^, 100); HRMS (ESI) *m*/*z*: [M + H]^+^ Calcd for C_23_H_28_N_4_O_4_H 425.2183; found 425.2183.

### (2*S*)-2-Amino-3-(2′-phenyl-4′-phenylpyrimidin-6′-yl)propanoic Acid Hydrochloride (**12a**)

*tert-*Butyl (2*S*)-2-(*tert*-butoxycarbonylamino)-3-(2′-phenyl-4′-phenylpyrimidin-6′-yl)propanoate (**11a**) (0.11 g, 0.23 mmol) was dissolved in acetonitrile (1 mL). To the reaction mixture was added 1 M hydrochloric acid (10 mL) and the mixture was stirred at room temperature for 18 h. The reaction mixture was then concentrated *in vacuo*. Purification by recrystallization from diethyl ether (5 mL) gave (2*S*)-2-amino-3-(2′-phenyl-4′-phenylpyrimidin-6′-yl)propanoic acid hydrochloride (**12a**) as a white solid (0.077 g, 95%). Mp 195–200 °C; IR (neat) 3357, 2932, 2158, 1717, 1574, 1537, 1377, 1029, 749, 688 cm^–1^; [α]_D_^20^ +19.8 (*c* 0.1, MeOH); ^1^H NMR (CD_3_OD, 400 MHz): δ 8.60–8.53 (m, 2H), 8.34–8.28 (m, 2H), 7.84 (s, 1H), 7.60–7.49 (m, 6H), 4.65 (t, *J* = 5.4 Hz, 1H), 3.63 (d, *J* = 5.4 Hz, 2H); ^13^C{^1^H} NMR (CD_3_OD, 101 MHz): δ 169.9, 164.9, 164.6, 164.2, 137.4, 136.5, 131.0, 130.7, 128.7, 128.2, 127.0, 114.1, 50.7, 35.8; MS (ESI) *m*/*z* 342 (M + Na^+^, 100); HRMS (ESI) *m*/*z*: [M + Na]^+^ Calcd for C_19_H_17_N_3_O_2_Na 342.1213; found 342.1212.

### (2*S*)-2-Amino-3-[4′-(2″-naphthyl)-2′-phenylpyrimidin-6′-yl]propanoic Acid Hydrochloride (**12b**)

(2*S*)-2-Amino-3-[4′-(2″-naphthyl)-2′-phenylpyrimidin-6′-yl]propanoic acid hydrochloride (**12b**) was prepared as described for **12a** using *tert-*butyl (2*S*)-2-(*tert*-butoxycarbonylamino)-3-[4′-(2″-naphthyl)-2′-phenylpyrimidin-6′-yl]propanoate (**11b**) (0.11 g, 0.23 mmol). Following completion, the reaction mixture was concentrated *in vacuo*. This gave (2*S*)-2-amino-3-[4′-(2″-naphthyl)-2′-phenylpyrimidin-6′-yl]propanoic acid hydrochloride (**12b**) as a colorless oil (0.077 g, 95%). IR (neat) 3305, 2826, 1746, 1571, 1537, 1377, 1082, 760, 697 cm^–1^; [α]_D_^24^ +19.1 (*c* 0.1, MeOH); ^1^H NMR (CD_3_OD, 400 MHz): δ 8.86 (br s, 1H), 8.64–8.59 (m, 2H), 8.42 (dd, *J* = 8.0, 3.6 Hz, 1H), 8.08–8.00 (m, 3H), 7.96–7.93 (m, 1H), 7.62–7.52 (m, 5H), 4.67 (t, *J* = 5.4 Hz, 1H), 3.67 (d, *J* = 5.4 Hz, 2H); ^13^C{^1^H} NMR (CD_3_OD, 101 MHz): δ 169.8, 164.8, 164.7, 164.0, 137.1, 135.0, 133.6, 133.3, 130.9, 128.8, 128.4, 128.33, 128.26, 127.6, 127.44, 127.42, 126.5, 123.7, 114.5, 50.7, 35.8; MS (ESI) *m*/*z* 370 (M + H^+^, 100); HRMS (ESI) *m*/*z*: [M + H]^+^ Calcd for C_23_H_19_N_3_O_2_H 370.1550; found 370.1558.

### (2*S*)-2-Amino-3-[4′-(4″-methoxyphenyl)-2′-phenylpyrimidin-6′-yl]propanoic Acid Hydrochloride (**12c**)

(2*S*)-2-Amino-3-[4′-(4″-methoxyphenyl)-2′-phenylpyrimidin-6′-yl]propanoic acid hydrochloride (**12c**) was prepared as described for **12a** using *tert-*butyl (2*S*)-2-(*tert*-butoxycarbonylamino)-3-[4′-(4″-methoxyphenyl)-2′-phenylpyrimidin-6′-yl]propanoate (**11c**) (0.124 g, 0.280 mmol). This gave (2*S*)-2-amino-3-[4′-(4″-methoxyphenyl)-2′-phenylpyrimidin-6′-yl]propanoic acid hydrochloride (**12c**) as a white solid (0.0764 g, 95%). Mp 175–180 °C; IR (neat) 3354, 2835, 1745, 1588, 1631, 1563, 1428, 1254, 1176, 1081, 840, 758 cm^–1^; [α]_D_^18^ +22.4 (*c* 0.1, MeOH); ^1^H NMR (CD_3_OD, 400 MHz): δ 8.53–8.50 (m, 2H), 8.34 (d, *J* = 8.6 Hz, 2H), 7.97 (s, 1H), 7.62–7.55 (m, 3H), 7.13 (d, *J* = 8.6 Hz, 2H), 4.71 (t, *J* = 5.6 Hz, 1H), 3.91 (s, 3H), 3.70 (d, *J* = 5.6 Hz, 2H); ^13^C{^1^H} NMR (CD_3_OD, 101 MHz): δ 169.4, 165.4, 163.7, 163.4, 162.1, 134.6, 131.9, 129.9, 128.6, 127.1, 114.4, 114.3, 54.9, 50.8, 35.1; MS (ESI) *m*/*z* 350 (M + H^+^, 100); HRMS (ESI) *m*/*z*: [M + H]^+^ Calcd for C_20_H_19_N_3_O_3_H 350.1499; found 350.1500.

### (2*S*)-2-Amino-3-[4′-(4″-cyanophenyl)-2′-phenylpyrimidin-6′-yl]propanoic Acid Hydrochloride (**12d**)

(2*S*)-2-Amino-3-[4′-(4″-cyanophenyl)-2′-phenylpyrimidin-6′-yl]propanoic acid hydrochloride (**12d**) was prepared as described for **12a** using *tert-*butyl (2*S*)-2-(*tert*-butoxycarbonylamino)-3-[4′-(4″-cyanophenyl)-2′-phenylpyrimidin-6′-yl]propanoate (**11d**) (0.088 g, 0.18 mmol). Following completion, the reaction mixture was concentrated *in vacuo*. This gave (2*S*)-2-amino-3-[4′-(4″-cyanophenyl)-2′-phenylpyrimidin-6′-yl]propanoic acid hydrochloride (**12d**) as a yellow oil (0.057 g, 92%). IR (neat) 3354, 2936, 1712, 1646, 1588, 1516, 1366, 1251, 1173, 770 cm^–1^; [α]_D_^22^ +15.0 (*c* 0.1, MeOH); ^1^H NMR (CD_3_OD, 400 MHz): δ 8.58–8.54 (m, 2H), 8.49 (d, *J* = 7.9 Hz, 2H), 7.96 (s, 1H), 7.92 (d, *J* = 7.9 Hz, 2H), 7.55–7.50 (m, 3H), 4.69 (t, *J* = 4.6 Hz, 1H), 3.69 (d, *J* = 4.6 Hz, 2H); ^13^C{^1^H} NMR (CD_3_OD, 101 MHz): δ 169.8, 165.7, 164.4, 162.6, 140.8, 137.0, 132.6, 130.9, 128.3, 128.2, 127.9, 117.9, 115.0, 114.2, 50.6, 36.0; MS (ESI) *m*/*z* 345 (M + H^+^, 100); HRMS (ESI) *m*/*z*: [M + H]^+^ Calcd for C_20_H_16_N_4_O_2_H 345.1346; found 345.1349.

### (2*S*)-2-Amino-3-(2′-methyl-4′-phenylpyrimidin-6′-yl)propanoic Acid Hydrochloride (**12e**)

(2*S*)-2-Amino-3-(2′-methyl-4′-phenylpyrimidin-6′-yl)propanoic acid hydrochloride (**12e**) was prepared as described for **12a** using *tert-*butyl (2*S*)-2-(*tert*-butoxycarbonylamino)-3-(2′-methyl-4′-phenylpyrimidin-6′-yl)propanoate (**11e**) (0.22 g, 0.53 mmol). This gave (2*S*)-2-amino-3-(2′-methyl-4′-phenylpyrimidin-6′-yl)propanoic acid hydrochloride (**12e**) as a white solid (0.13 g, 95%). Mp 145–149 °C; IR (neat) 3371, 2918, 1745, 1612, 1593, 1505, 1440, 1368, 1223, 1055, 752, 695 cm^–1^; [α]_D_^24^ +17.6 (*c* 0.1, MeOH); ^1^H NMR (CD_3_OD, 400 MHz): δ 8.36 (s, 1H), 8.26–8.22 (m, 2H), 7.75–7.62 (m, 3H), 4.81–4.75 (m, 1H), 3.80 (dd, *J* = 16.4, 6.1 Hz, 1H), 3.72 (dd, *J* = 16.4, 7.1 Hz, 1H), 2.98 (s, 3H); ^13^C{^1^H} NMR (CD_3_OD, 101 MHz): δ 168.9, 166.7, 164.61, 164.55, 133.2, 132.4, 129.3, 128.5, 116.6, 50.6, 35.4, 22.0; MS (ESI) *m*/*z* 258 (M + H^+^, 100); HRMS (ESI) *m*/*z*: [M + H]^+^ Calcd for C_14_H_15_N_3_O_2_H 258.1237; found 258.1238.

### (2*S*)-2-Amino-3-[2′-methyl-4′-(2″-naphthyl)pyrimidin-6′-yl]propanoic Acid Hydrochloride (**12f**)

(2*S*)-2-Amino-3-[2′-methyl-4′-(2″-naphthyl)pyrimidin-6′-yl]propanoic acid hydrochloride (**12f**) was prepared as described for **12a** using *tert-*butyl (2*S*)-2-(*tert*-butoxycarbonylamino)-3-[2′-methyl-4′-(2″-naphthyl)pyrimidin-6′-yl]propanoate (**11f**) (0.0650 g, 0.14 mmol). Following completion, the reaction mixture was concentrated *in vacuo*. This gave (2*S*)-2-amino-3-[2′-methyl-4′-(2″-naphthyl)pyrimidin-6′-yl]propanoic acid hydrochloride (**12f**) as a colorless oil (0.040 g, 93%). IR (neat) 3354, 2952, 1724, 1512, 1347, 1214, 1056, 812, 743 cm^–1^; [α]_D_^22^ +18.0 (*c* 0.1, MeOH); ^1^H NMR (CD_3_OD, 400 MHz): δ 8.87 (s, 1H), 8.54 (s, 1H), 8.21 (d, *J* = 8.9 Hz, 1H), 8.10–8.03 (m, 2H), 7.94 (d, *J* = 8.9 Hz, 1H), 7.67–7.56 (m, 2H), 4.82–4.79 (m, 1H), 3.82 (dd, *J* = 16.5, 4.9 Hz, 1H), 3.78 (dd, *J* = 16.5, 6.9 Hz, 1H), 2.97 (s, 3H); ^13^C{^1^H} NMR (CD_3_OD, 101 MHz): δ 168.7, 168.0, 165.4, 163.7, 135.8, 132.9, 130.9, 129.4, 129.3, 129.2, 128.9, 127.6, 127.3, 123.8, 117.2, 50.7, 35.0, 21.5; MS (ESI) *m*/*z* 308 (M + H^+^, 100); HRMS (ESI) *m*/*z*: [M + H]^+^ Calcd for C_18_H_17_N_3_O_2_H 308.1394; found 308.1399.

### (2*S*)-2-Amino-3-[4′-(4″-methoxyphenyl)-2′-methylpyrimidin-6′-yl]propanoic Acid Hydrochloride (**12g**)

(2*S*)-2-Amino-3-[4′-(4″-methoxyphenyl)-2′-methylpyrimidin-6′-yl]propanoic acid hydrochloride (**12g**) was prepared as described for **12a** using *tert-*butyl (2*S*)-2-(*tert*-butoxycarbonylamino)-3-[4′-(4″-methoxyphenyl)-2′-methylpyrimidin-6′-yl]propanoate (**11g**) (0.12 g, 0.28 mmol). This gave (2*S*)-2-amino-3-[4′-(4″-methoxyphenyl)-2′-methylpyrimidin-6′-yl]propanoic acid hydrochloride (**12g**) as a white solid (0.077 g, 95%). Mp 174–180 °C; IR (neat) 3262, 2830, 1742, 1584, 1251, 1180, 1031, 894, 621 cm^–1^; [α]_D_^18^ +13.0 (*c* 0.1, MeOH); ^1^H NMR (CD_3_OD, 400 MHz): δ 8.23 (dd, *J* = 7.9, 2.1 Hz, 2H), 8.16 (s, 1H), 7.17 (dd, *J* = 7.9, 2.1 Hz, 2H), 4.70 (t, *J* = 6.7 Hz, 1H), 3.93 (s, 3H), 3.68 (dd, *J* = 16.2, 6.7 Hz, 1H), 3.60 (dd, *J* = 16.2, 6.7 Hz, 1H), 2.90 (s, 3H); ^13^C{^1^H} NMR (CD_3_OD, 101 MHz): δ 168.8, 164.9, 163.83, 163.82, 130.8, 124.0, 115.4, 114.9, 55.0, 50.7, 35.1, 21.7; MS (ESI) *m*/*z* 288 (M + H^+^, 100); HRMS (ESI) *m*/*z*: [M + H]^+^ Calcd for C_15_H_17_N_3_O_3_H 288.1343; found 288.1349.

### (2*S*)-2-Amino-3-[4′-(4″-cyanophenyl)-2′-methylpyrimidin-6′-yl]propanoic Acid Hydrochloride (**12h**)

(2*S*)-2-Amino-3-[4′-(4″-cyanophenyl)-2′-methylpyrimidin-6′-yl]propanoic acid hydrochloride (**12h**) was prepared as described for **12a** using *tert-*butyl (2*S*)-2-(*tert*-butoxycarbonylamino)-3-[4′-(4″-cyanophenyl)-2′-methylpyrimidin-6′-yl]propanoate (**11h**) (0.065 g, 0.15 mmol). Following completion, the reaction mixture was concentrated *in vacuo*. This gave (2*S*)-2-amino-3-[4′-(4″-cyanophenyl)-2′-methylpyrimidin-6′-yl]propanoic acid (**12h**) as a yellow oil (0.040 g, 94%). IR (neat) 3325, 2922, 1641, 1592, 1526, 1490, 1362, 1011 cm^–1^; [α]_D_^25^ +14.0 (*c* 0.1, MeOH); ^1^H NMR (CD_3_OD, 400 MHz): δ 8.36–8.32 (m, 2H), 7.94–7.89 (m, 3H), 4.61 (dd, *J* = 7.4, 4.7 Hz, 1H), 3.60 (dd, *J* = 16.7, 4.7 Hz, 1H), 3.51 (dd, *J* = 16.7, 7.4 Hz, 1H), 2.79 (s, 3H); ^13^C{^1^H} NMR (CD_3_OD, 101 MHz): δ 169.4, 167.8, 165.7, 162.9, 140.2, 132.5, 128.0, 117.8, 114.6, 114.5, 50.8, 35.5, 24.1; MS (ESI) *m*/*z* 283 (M + H^+^, 100); HRMS (ESI) *m*/*z*: [M + H]^+^ Calcd for C_15_H_14_N_4_O_2_H 283.1190; found 283.1189.

### (2*S*)-2-Amino-3-(4′-phenylpyrimidin-6′-yl)propanoic Acid Hydrochloride (**12i**)

(2*S*)-2-Amino-3-(4′-phenylpyrimidin-6′-yl)propanoic acid hydrochloride (**12i**) was prepared as described for **12a** using *tert-*butyl (2*S*)-2-(*tert*-butoxycarbonylamino)-3-(4′-phenylpyrimidin-6′-yl)propanoate (**11i**) (0.12 g, 0.31 mmol). This gave (2*S*)-2-amino-3-(4′-phenylpyrimidin-6′-yl)propanoic acid hydrochloride (**12i**) as a white solid (0.073 g, 95%). Mp 68–70 °C; IR (neat) 3440, 2891, 1741, 1631, 1587, 1466, 1325, 1228, 1160, 970, 693 cm^–1^; [α]_D_^18^ +21.0 (*c* 0.1, MeOH); ^1^H NMR (CD_3_OD, 400 MHz): δ 9.33 (s, 1H), 8.34 (s, 1H), 8.22 (d, *J* = 8.0 Hz, 2H), 7.71–7.57 (m, 3H), 4.73–4.68 (m, 1H), 3.74 (dd, *J* = 16.5, 5.3 Hz, 1H), 3.66 (dd, *J* = 16.5, 6.9 Hz, 1H); ^13^C{^1^H} NMR (CD_3_OD, 101 MHz): δ 169.1, 166.9, 163.9, 155.0, 133.4, 132.7, 129.2, 127.9, 118.5, 50.7, 35.7; MS (ESI) *m*/*z* 244 (M + H^+^, 100); HRMS (ESI) *m*/*z*: [M + H]^+^ Calcd for C_13_H_13_N_3_O_2_H 244.1081; found 244.1085.

### (2*S*)-2-Amino-3-[4′-(2″-naphthyl)pyrimidin-6′-yl]propanoic Acid Hydrochloride (**12j**)

(2*S*)-2-Amino-3-[4′-(2″-naphthyl)pyrimidin-6′-yl]propanoic acid hydrochloride (**12j**) was prepared as described for **12a** using *tert-*butyl (2*S*)-2-(*tert*-butoxycarbonylamino)-3-[4′-(2″-naphthyl)pyrimidin-6′-yl]propanoate (**11j**) (0.029 g, 0.070 mmol). This gave (2*S*)-2-amino-3-[4′-(2″-naphthyl)pyrimidin-6′-yl]propanoic acid hydrochloride (**12j**) as a white solid (0.019 g, 95%). Mp 153–155 °C; IR (neat) 3429, 2977, 1706, 1583, 1364, 1250, 1149, 729 cm^–1^; [α]_D_^19^ +18.2 (*c* 0.1, MeOH); ^1^H NMR (CD_3_OD, 400 MHz): δ 9.29 (s, 1H), 8.78 (br s, 1H), 8.42 (s, 1H), 8.21 (d, *J* = 8.4 Hz, 1H), 8.05–8.00 (m, 2H), 7.91 (d, *J* = 8.9 Hz, 1H), 7.64–7.53 (m, 2H), 4.72–4.67 (m, 1H), 3.71 (dd, *J* = 16.8, 4.7 Hz, 1H), 3.64 (dd, *J* = 16.8, 7.2 Hz, 1H); ^13^C{^1^H} NMR (CD_3_OD, 101 MHz): δ 169.2, 166.2, 164.1, 155.3, 135.4, 133.1, 130.8, 129.2, 129.1, 129.0, 128.4, 127.5, 126.9, 123.5, 118.4, 50.8, 35.6; MS (ESI) *m*/*z* 292 ([M – H]^−^, 100); HRMS (ESI) *m*/*z*: [M – H]^−^ Calcd for C_17_H_14_N_3_O_2_ 292.1092; found 292.1093.

### (2*S*)-2-Amino-3-[4′-(4″-methoxyphenyl)pyrimidin-6′-yl]propanoic Acid Hydrochloride (**12k**)

(2*S*)-2-Amino-3-[4′-(4″-methoxyphenyl)pyrimidin-6′-yl]propanoic acid hydrochloride (**12k**) was prepared as described for **12a** using *tert-*butyl (2*S*)-2-(*tert*-butoxycarbonylamino)-3-[4′-(4″-methoxyphenyl)pyrimidin-6′-yl]propanoate (**11k**) (0.73 g, 1.7 mmol). Following completion, the reaction mixture was concentrated *in vacuo*. This gave (2*S*)-2-amino-3-[4′-(4″-methoxyphenyl)pyrimidin-6′-yl]propanoic acid hydrochloride (**12k**) as a yellow oil (0.44 g, 95%); IR (neat) 3401, 2844, 1749, 1591, 1462, 1260, 1181, 1020, 841 cm^–1^; [α]_D_^23^ +15.0 (*c* 0.1, MeOH); ^1^H NMR (CD_3_OD, 400 MHz): δ 9.24 (s, 1H), 8.25 (d, *J* = 8.6 Hz, 2H), 8.22 (s, 1H), 7.15 (d, *J* = 8.6 Hz, 2H), 4.72–4.64 (m, 1H), 3.92 (s, 3H), 3.74–3.56 (m, 2H); ^13^C{^1^H} NMR (CD_3_OD, 101 MHz): δ 169.1, 165.9, 164.3, 163.4, 154.4, 130.0, 125.0, 117.2, 114.7, 54.9, 50.7, 35.5; MS (ESI) *m*/*z* 274 (M + H^+^, 100); HRMS (ESI) *m*/*z*: [M + H]^+^ Calcd for C_14_H_15_N_3_O_3_H 274.1186; found 274.1192.

### (2*S*)-2-Amino-3-[4′-(4″-cyanophenyl)pyrimidin-6′-yl]propanoic Acid Hydrochloride (**12l**)

(2*S*)-2-Amino-3-[4′-(4″-cyanophenyl)pyrimidin-6′-yl]propanoic acid hydrochloride (**12l**) was prepared as described for **12a** using *tert-*butyl (2*S*)-2-(*tert*-butoxycarbonylamino)-3-[4′-(4″-cyanophenyl)pyrimidin-6′-yl]propanoate (**11l**) (0.068 g, 0.16 mmol). Following completion, the reaction mixture was concentrated *in vacuo*. This gave (2*S*)-2-amino-3-[4′-(4″-cyanophenyl)pyrimidin-6′-yl]propanoic acid hydrochloride (**12l**) as a colorless oil (0.041 g, 95%). IR (neat) 3372, 2977, 1739, 1595, 1505, 1185, 1139, 837 cm^–1^; [α]_D_^22^ +12.0 (*c* 0.1, MeOH); ^1^H NMR (CD_3_OD, 400 MHz): δ 9.25 (s, 1H), 8.36 (d, *J* = 8.1 Hz, 2H), 8.25 (s, 1H), 7.88 (d, *J* = 8.1 Hz, 2H), 4.67–4.62 (m, 1H), 3.67 (dd, *J* = 16.6, 4.0 Hz, 1H), 3.59 (dd, *J* = 16.6, 7.2 Hz, 1H); ^13^C{^1^H} NMR (CD_3_OD, 101 MHz): δ 169.3, 165.9, 162.8, 157.1, 139.5, 132.7, 128.2, 118.2, 117.8, 114.7, 50.8, 35.5; MS (ESI) *m*/*z* 269 (M + H^+^, 100); HRMS (ESI) *m*/*z*: [M + H]^+^ Calcd for C_14_H_12_N_4_O_2_H 269.1033; found 269.1037.

## Data Availability

The data underlying this study are available in the published article and its online Supporting Material.

## Supporting Information Available

Photophysical data for α-amino acids **12a**–**l** and ^1^H and ^13^C NMR spectra for all compounds (PDF)
