# Supplementary material for: Synthesis and Photophysical Properties of Charge-Transfer-Based Pyrimidine-Derived α-Amino Acids
Source: J Org Chem. 2023 Aug 25;88(18):13214–24. doi: 10.1021/acs.joc.3c01437 (PMC10507667; doi:10.1021/acs.joc.3c01437)

Supporting Information for:

Synthesis and Photophysical Properties of Charge-Transfer Based Pyrimidine-Derived α-Amino Acids

*Sineenard Songsri, Alexander H. Harkiss and Andrew Sutherland**

School of Chemistry, The Joseph Black Building, University of Glasgow,

Glasgow G12 8QQ, United Kingdom.

Table of Contents

1. Photophysical Data for α-Amino Acids **12a–l** S2–S16
2. ^{1}H and ^{13}C NMR Spectra for all Compounds S17–S74

1. Photophysical Data for α-Amino Acids 12a–l

Table S1. Photophysical Data of Pyrimidine-Derived α-Amino Acids 12a–l.

amino acid	λ_{Abs} (nm)[a]	ε (cm^{-1} M^{-1})	λ_{Em} (nm)[a]	Φ_F[b]	brightness (cm^{-1} M^{-1})
**12a**	262	27800	396	0.0017	47
**12b**	311	24000	497	0.12	2880
**12c**	305	12700	314	0.003	38
**12d**	265	38700	316	0.004	155
**12e**	279	12600	308	0.002	25
**12f**	305	12800	490	0.11	1408
**12g**	299	16400	314, 381	0.016	262
**12h**	284	5300	306	0.003	16
**12i**	275	10700	308	0.003	32
**12j**	310	10400	421	0.30	3120
**12k**	306	13600	384	0.27	3672
**12l**	279	12,800	404	0.0007	9

[a]Spectra were recorded at 2 μM in methanol. [b]Quantum yields (Φ_F) were determined in methanol using anthracene and L-tryptophan as standards.

Absorption and Emission Spectra for 12a.

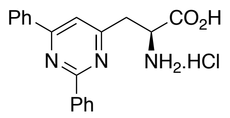

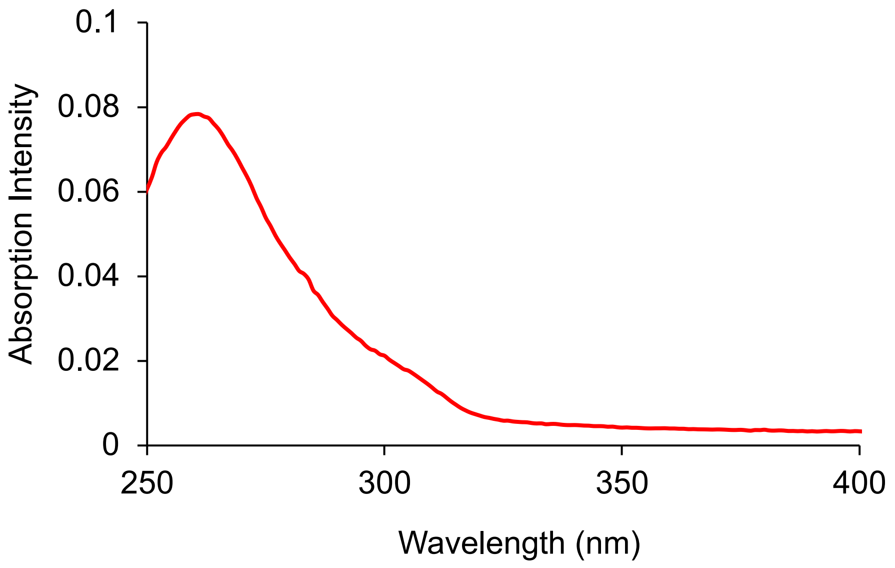

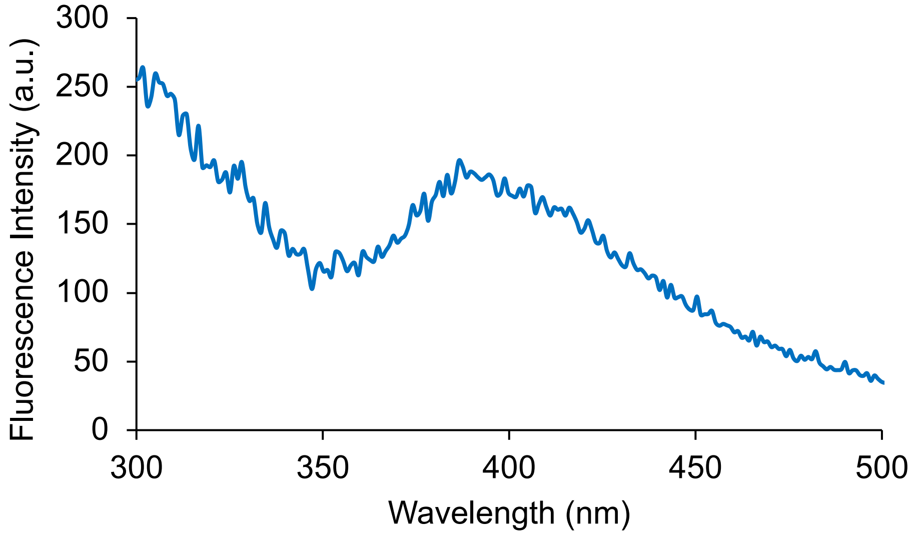

Absorption and Emission Spectra for 12b.

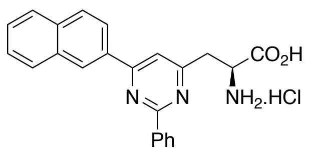

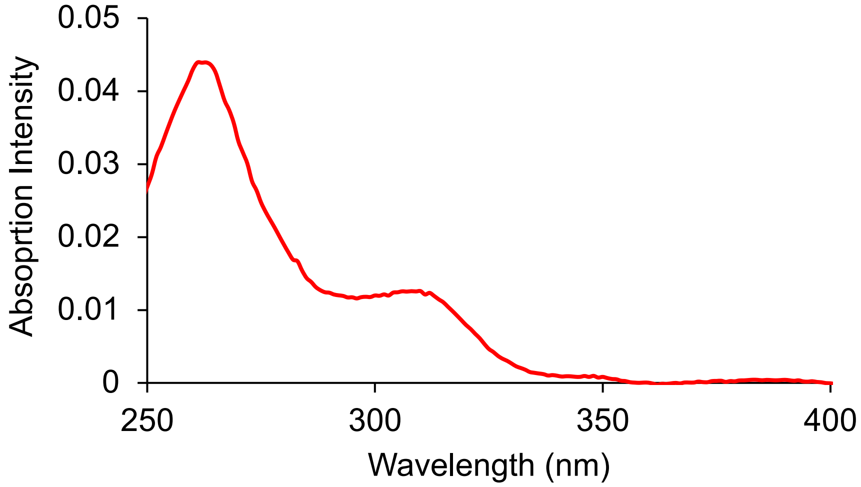

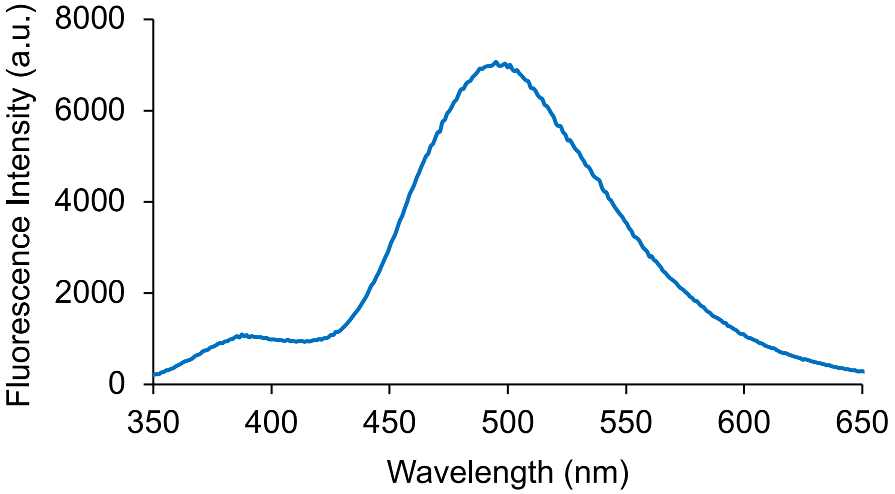

Absorption and Emission Spectra for 12c.

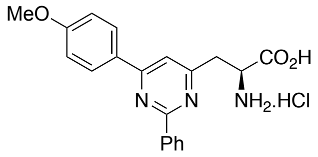

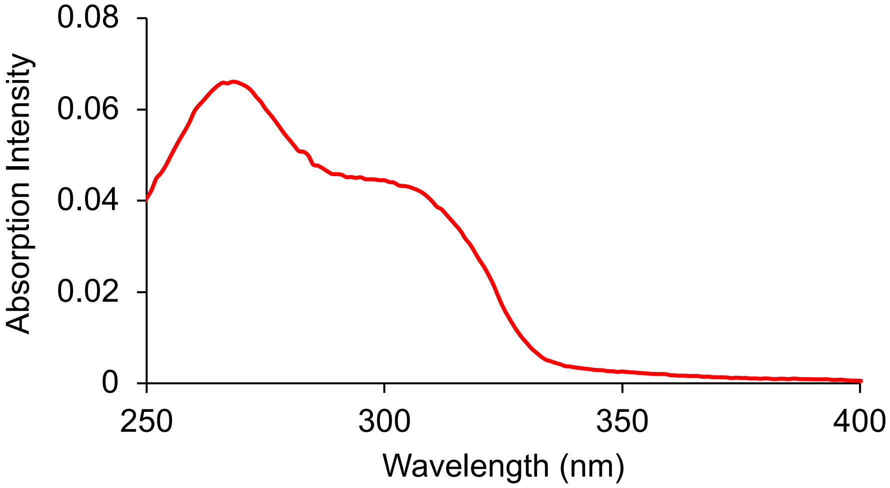

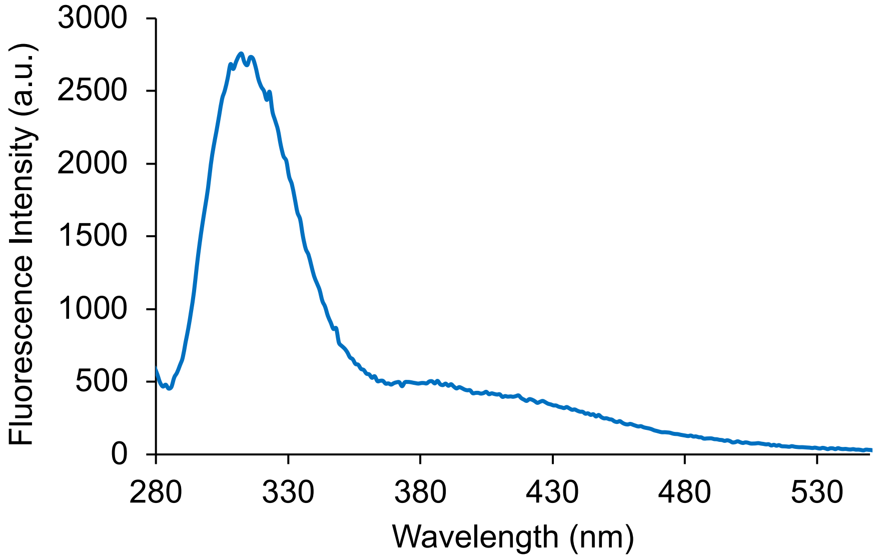

Absorption and Emission Spectra for 12d.

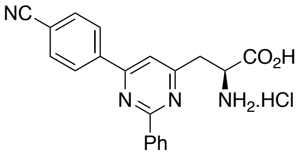

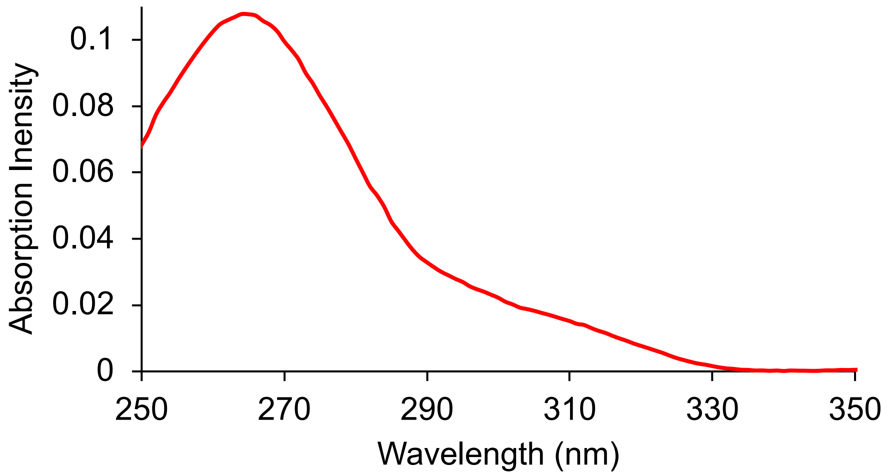

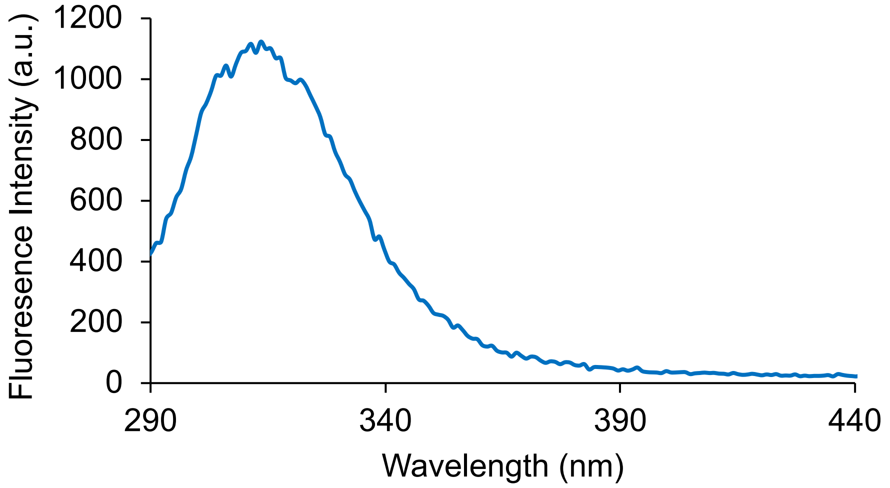

Absorption and Emission Spectra for 12e.

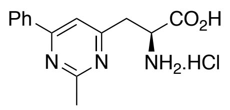

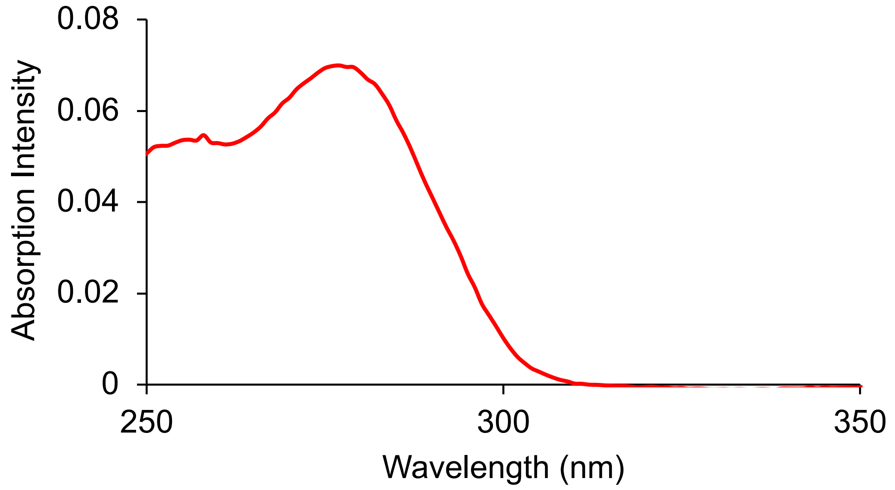

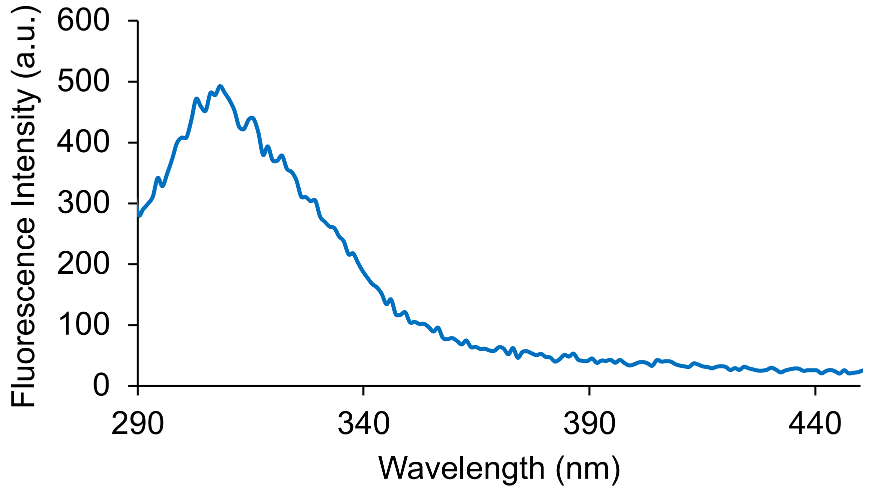

Absorption and Emission Spectra for 12f.

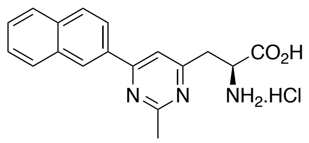

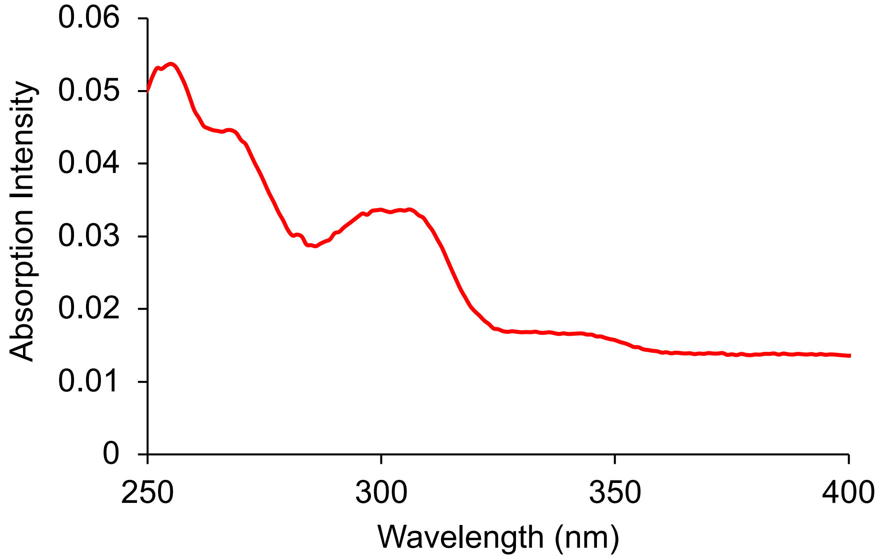

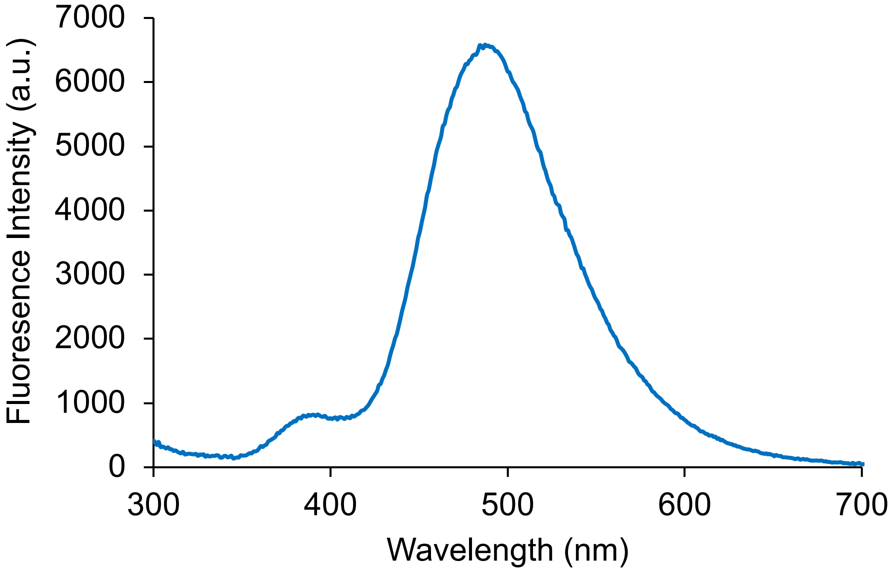

Absorption and Emission Spectra for 12g.

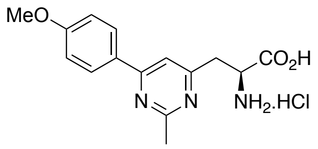

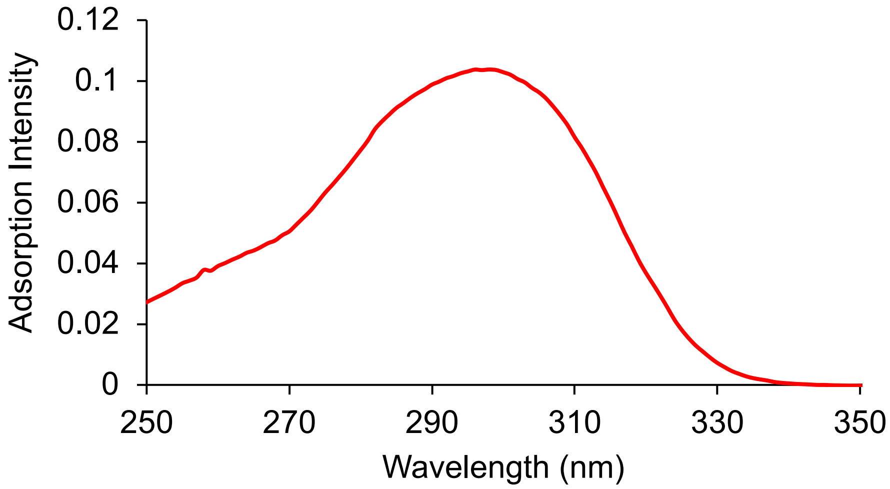

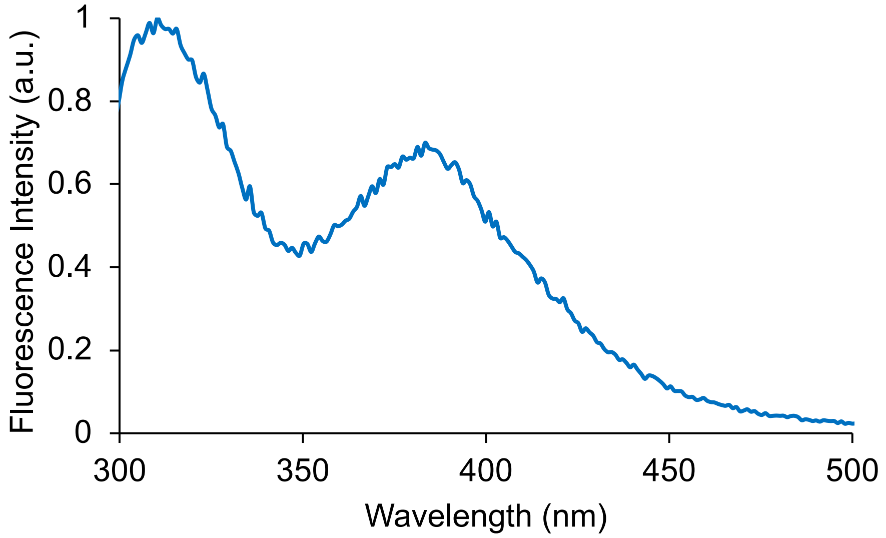

Absorption and Emission Spectra for 12h.

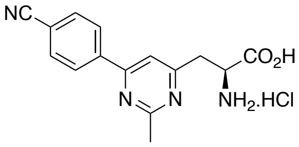

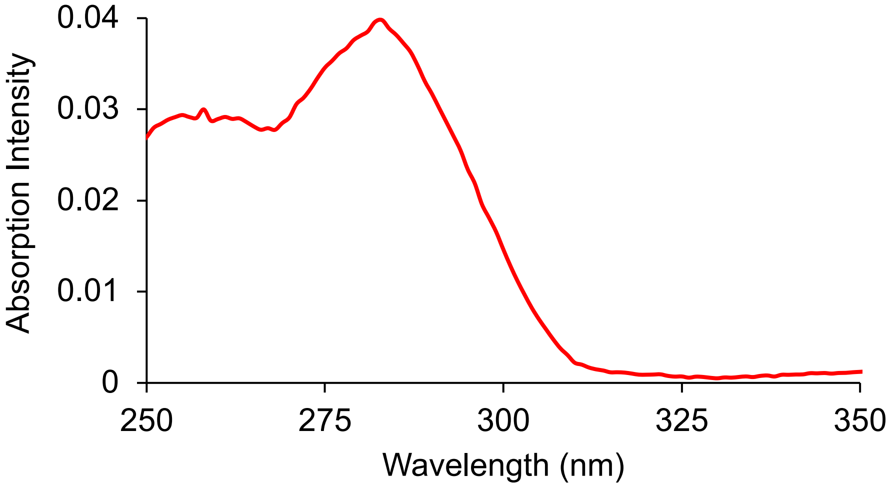

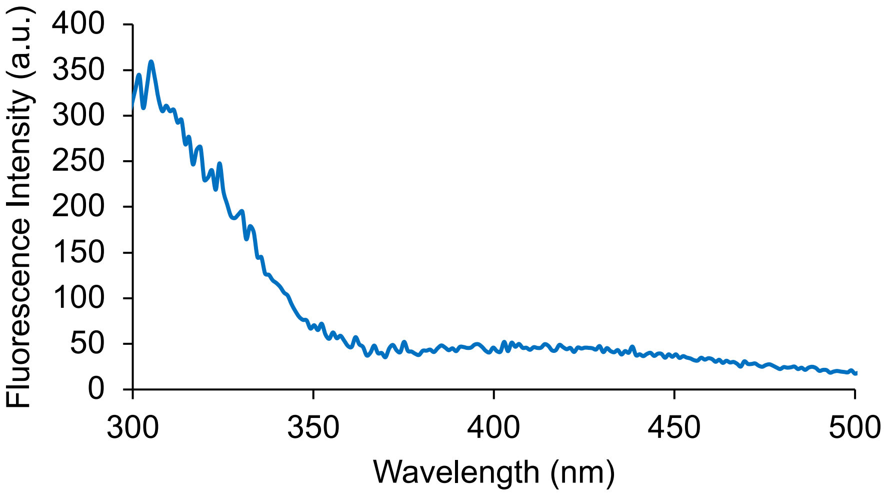

Absorption and Emission Spectra for 12i.

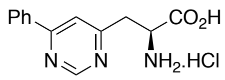

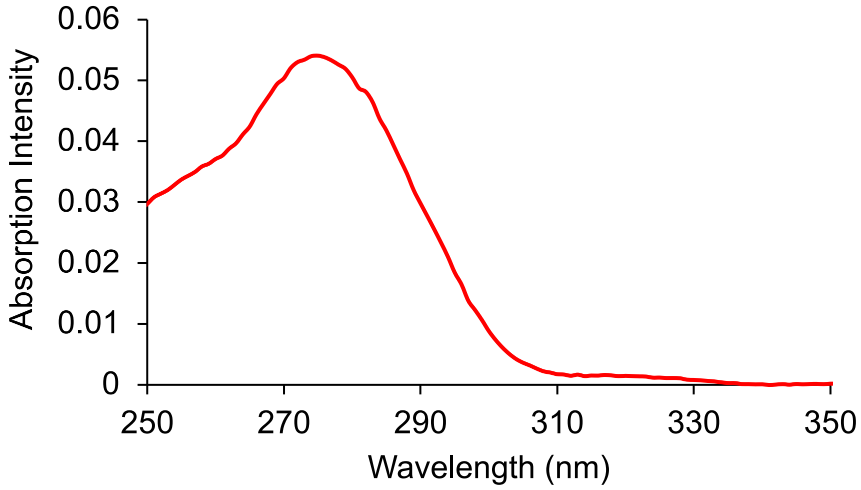

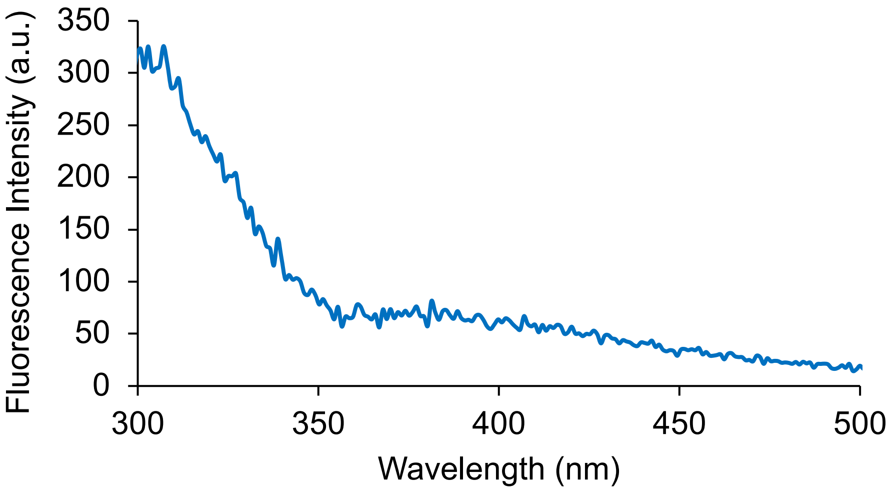

Absorption and Emission Spectra for 12j.

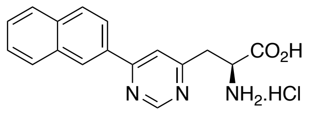

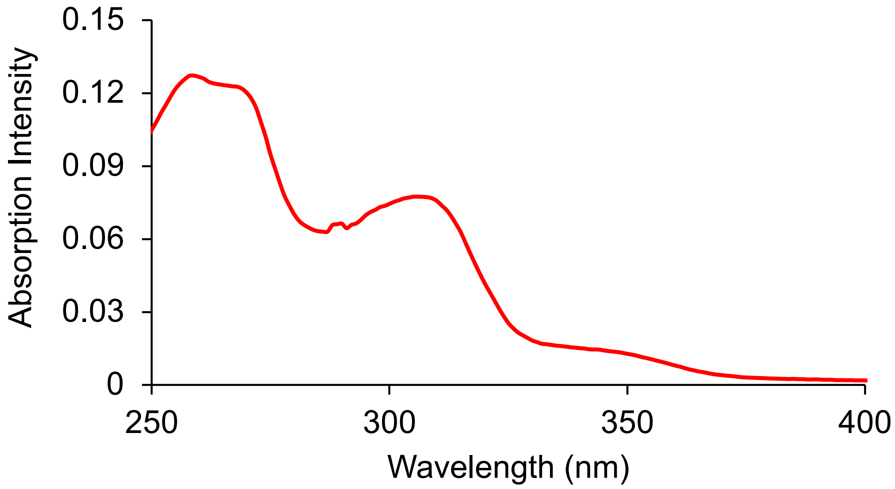

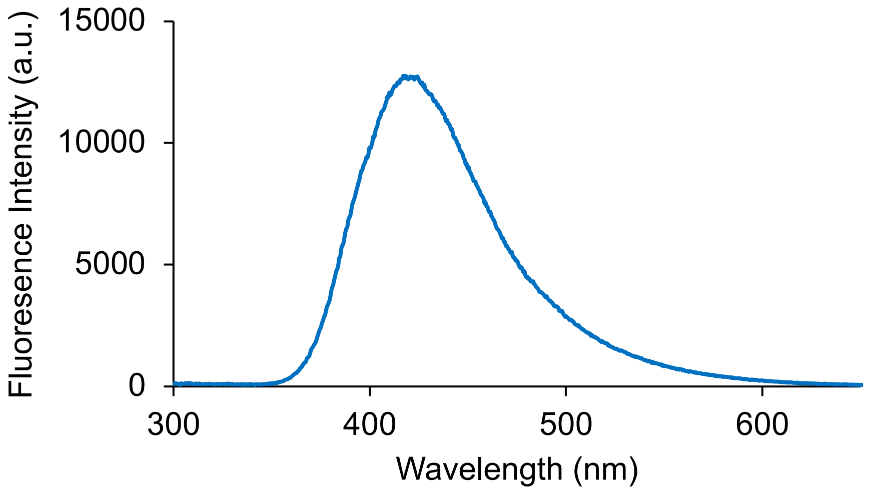

Absorption and Emission Spectra for 12k.

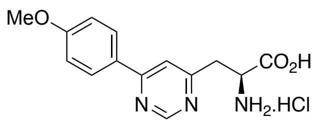

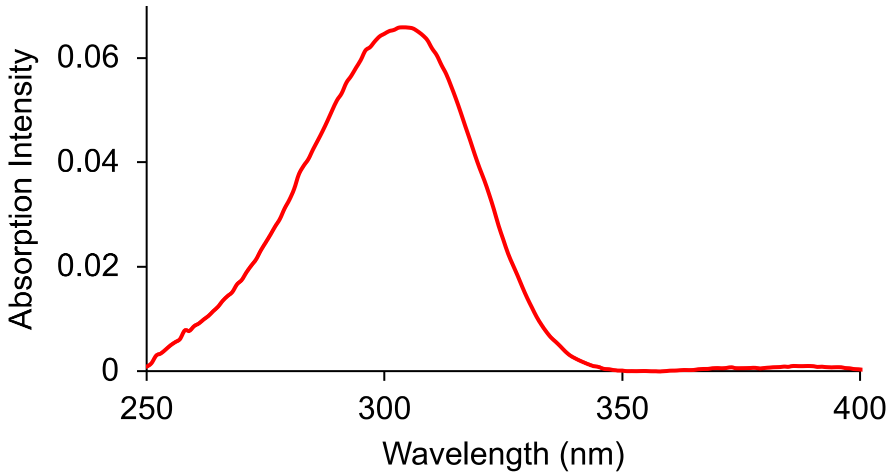

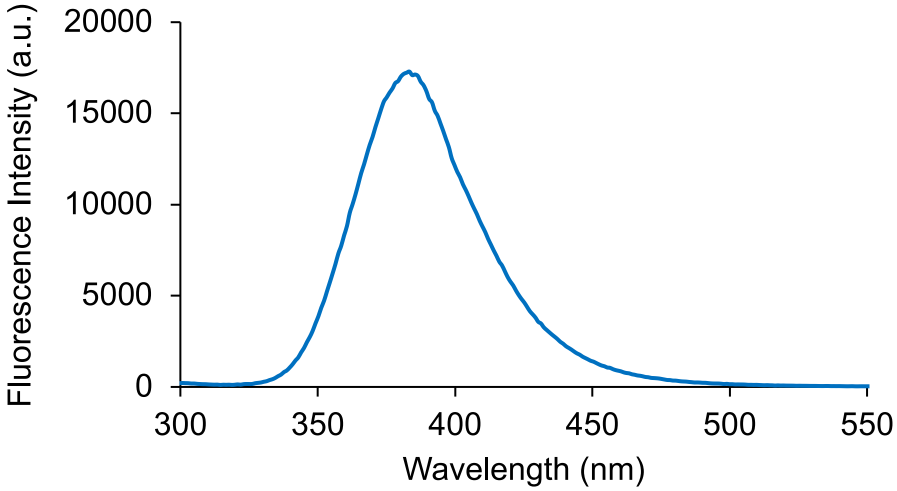

Absorption and Emission Spectra for 12l.

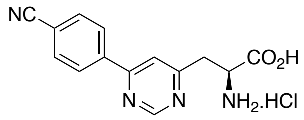

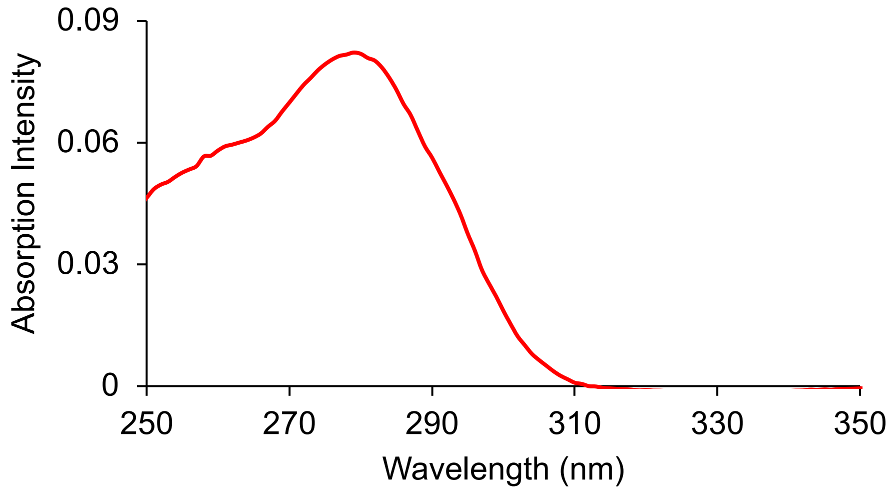

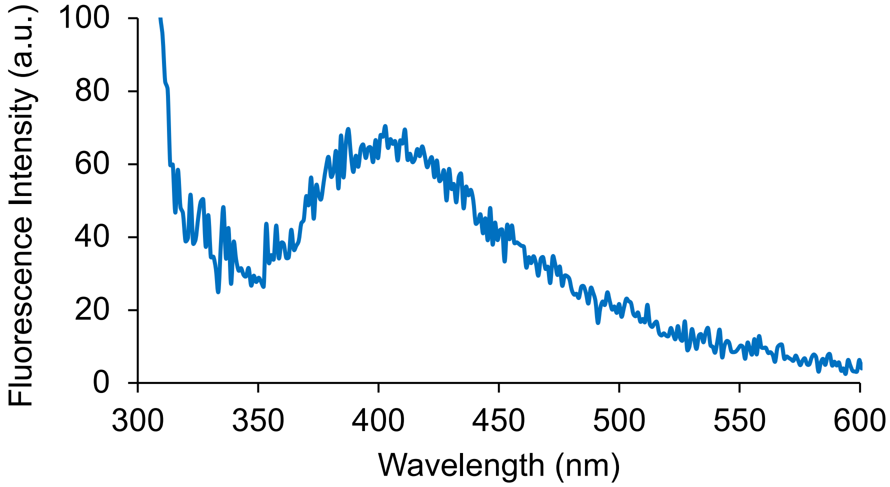

Absorption and Emission Spectra for Solvatochromic Study of 12k.

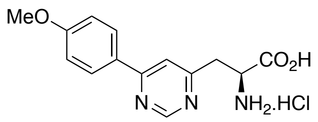

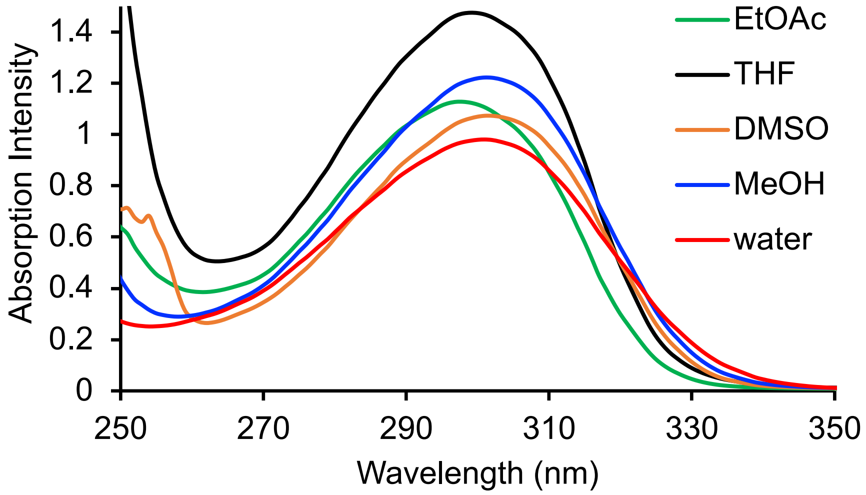

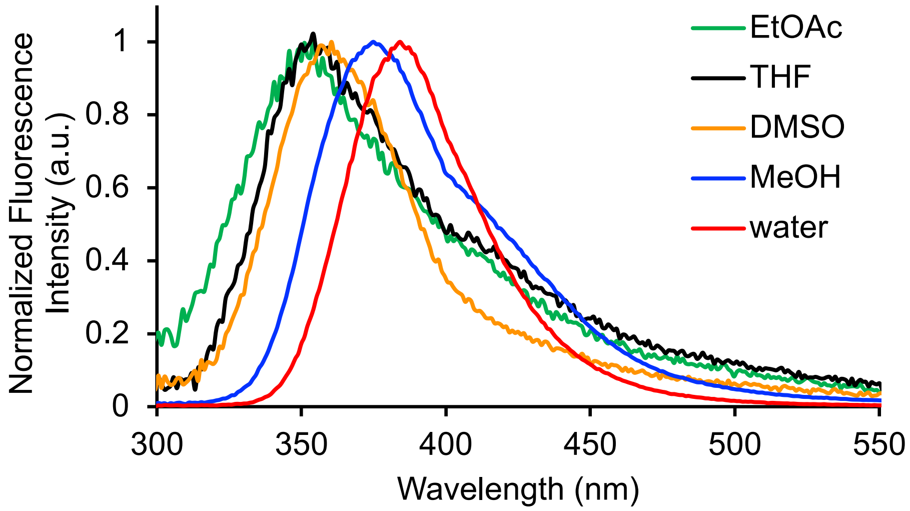

Lippert-Mataga Plot for 12k.

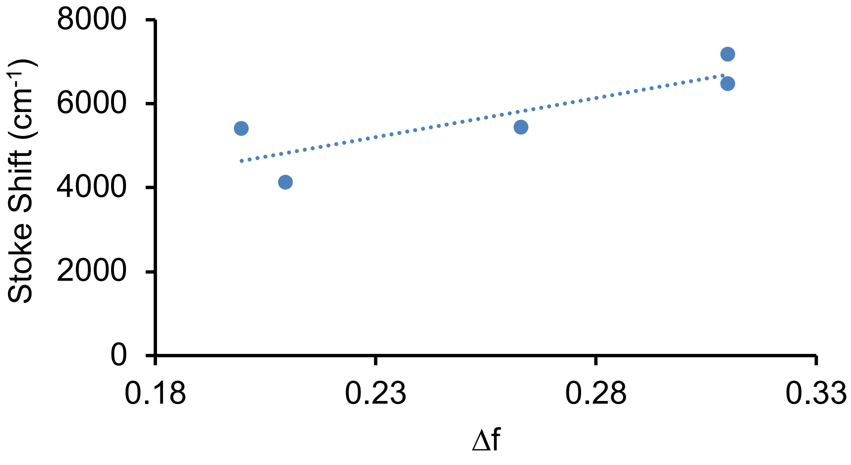

Absorption and Emission Spectra for pH Study of 12k.

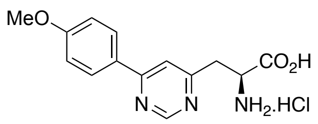

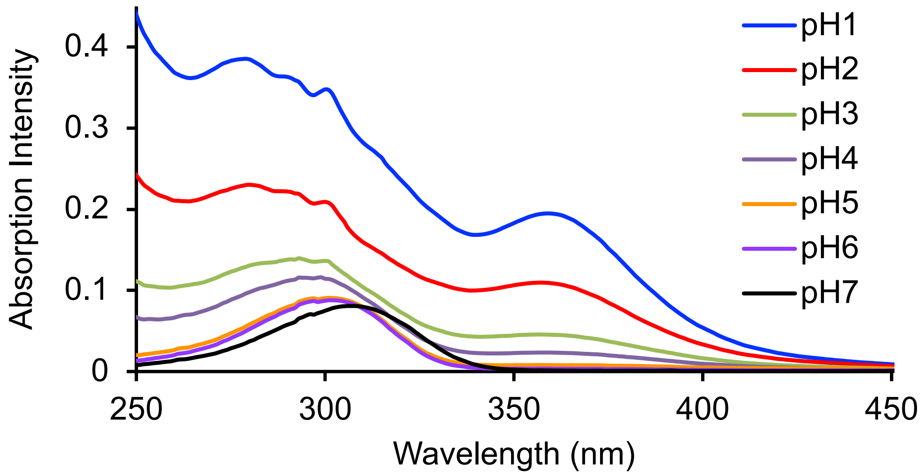

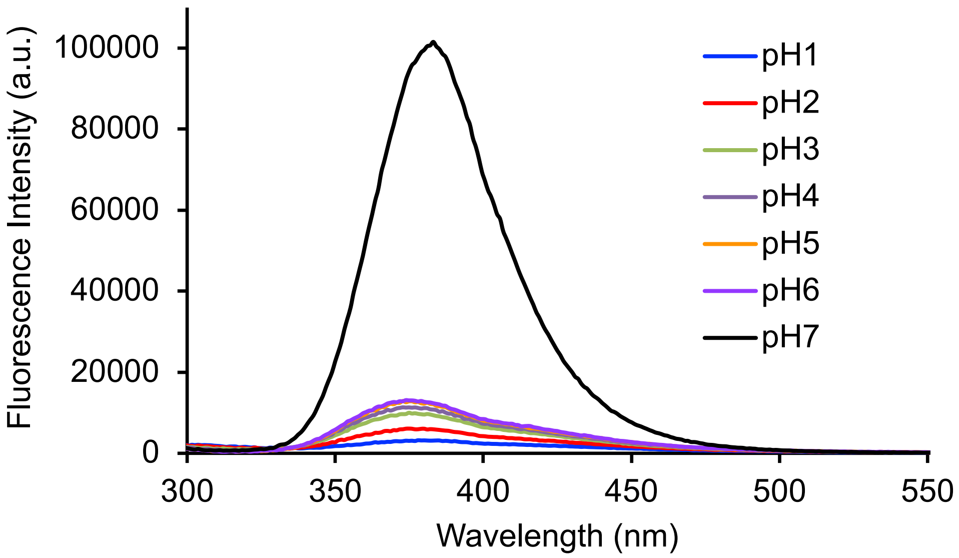

2. ^{1}H and ^{13}C NMR Spectra for all Compounds

^{1}H NMR (400 MHz, $CDCl_3$)

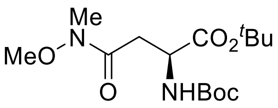

9

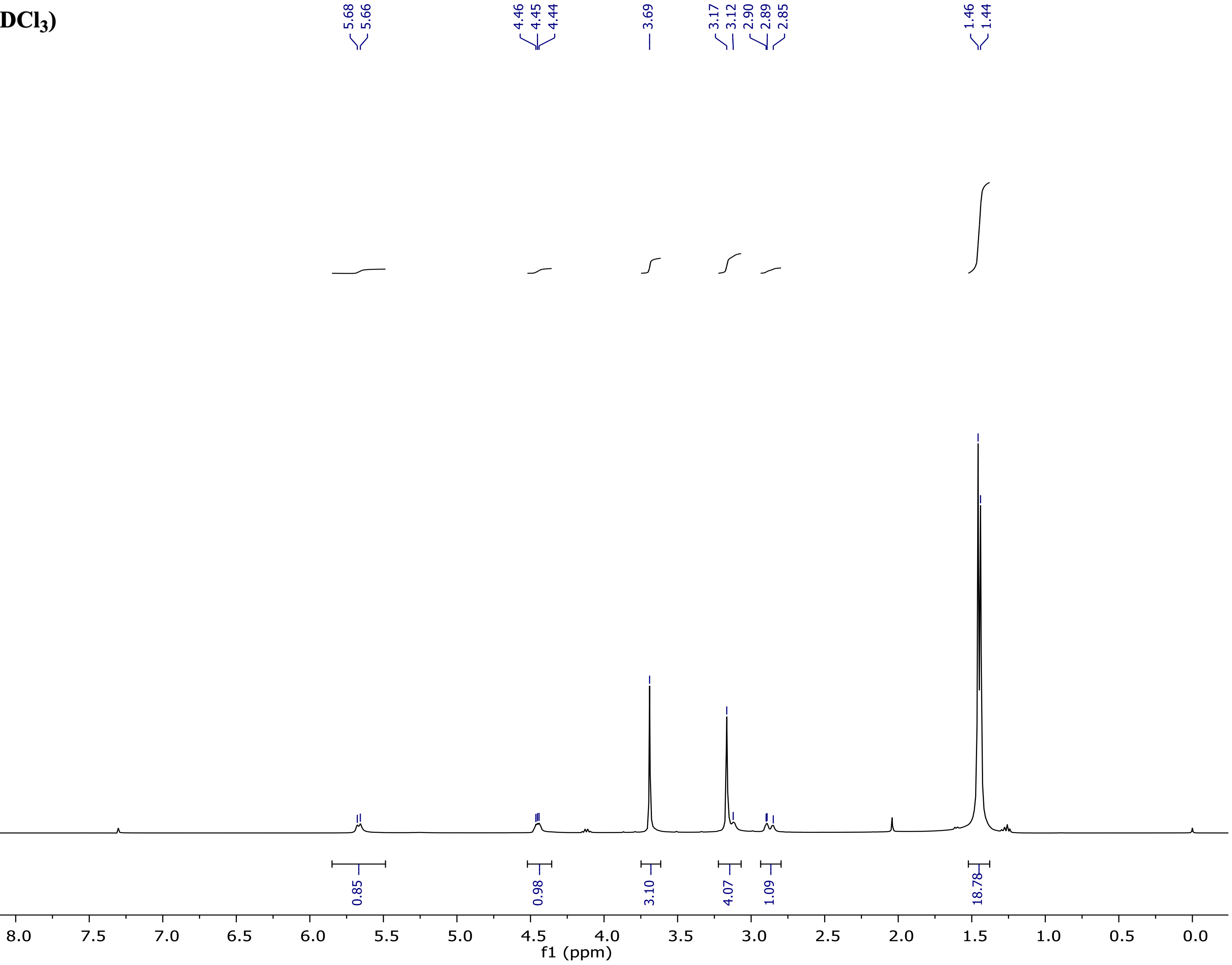

$^{13}C\{^1H\}$ NMR (101 MHz, $CDCl_3$)

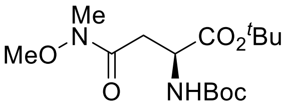

9

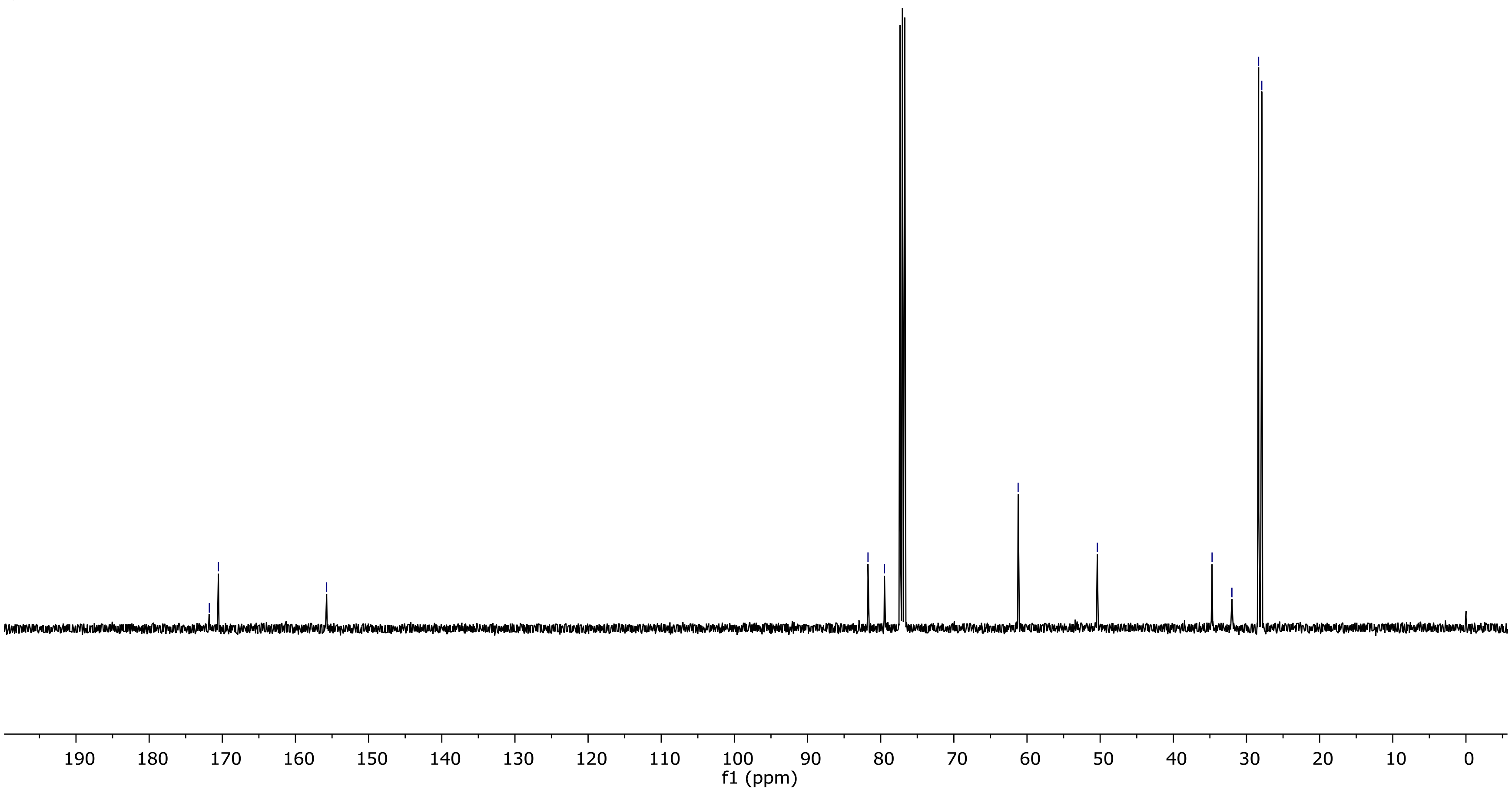

^{1}H NMR (400 MHz, $CDCl_3$)

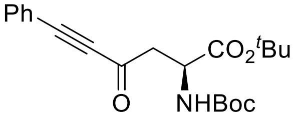

10a

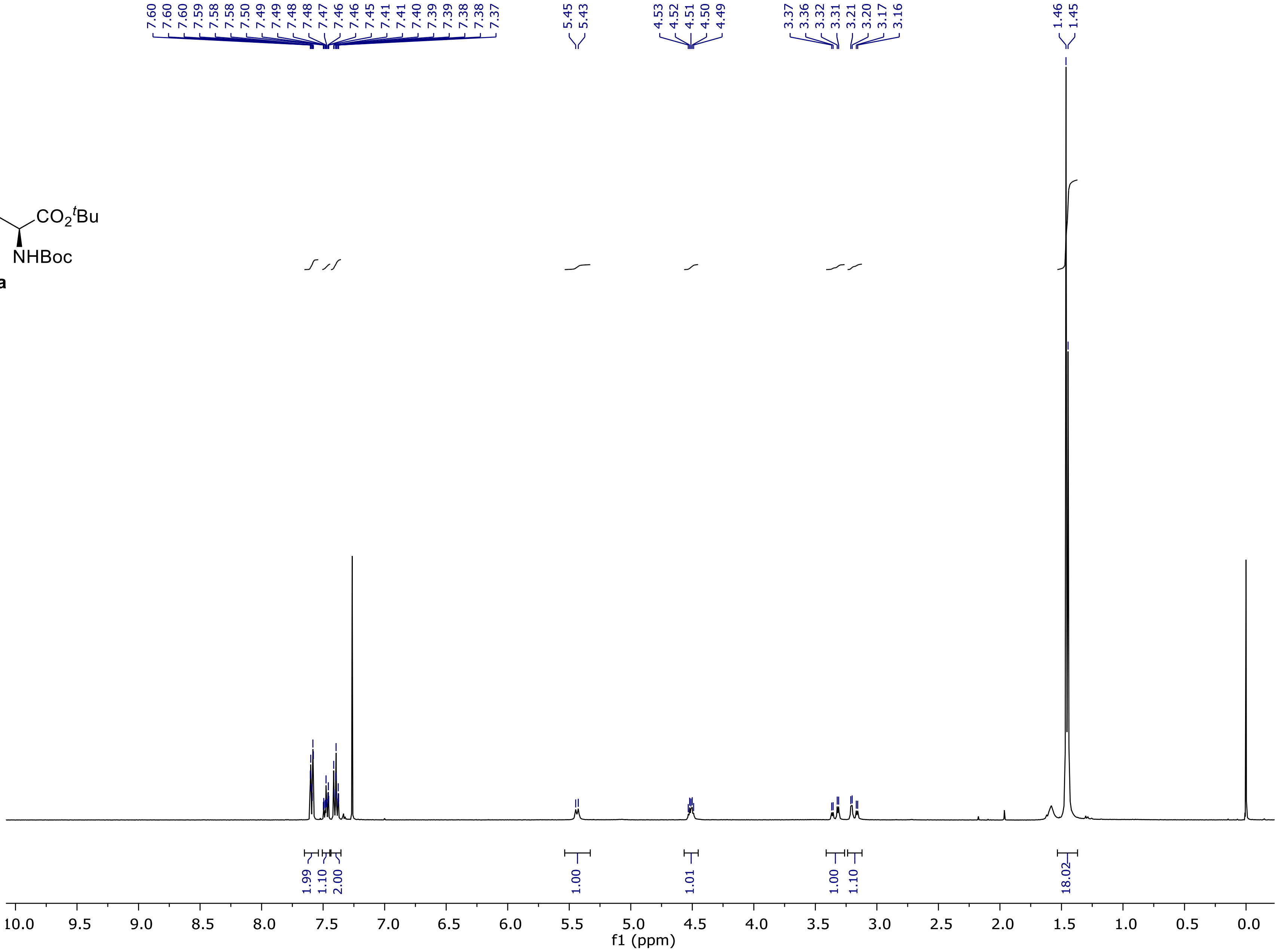

$^{13}C\{^1H\}$ NMR (101 MHz, $CDCl_3$)

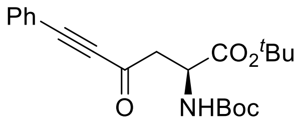

10a

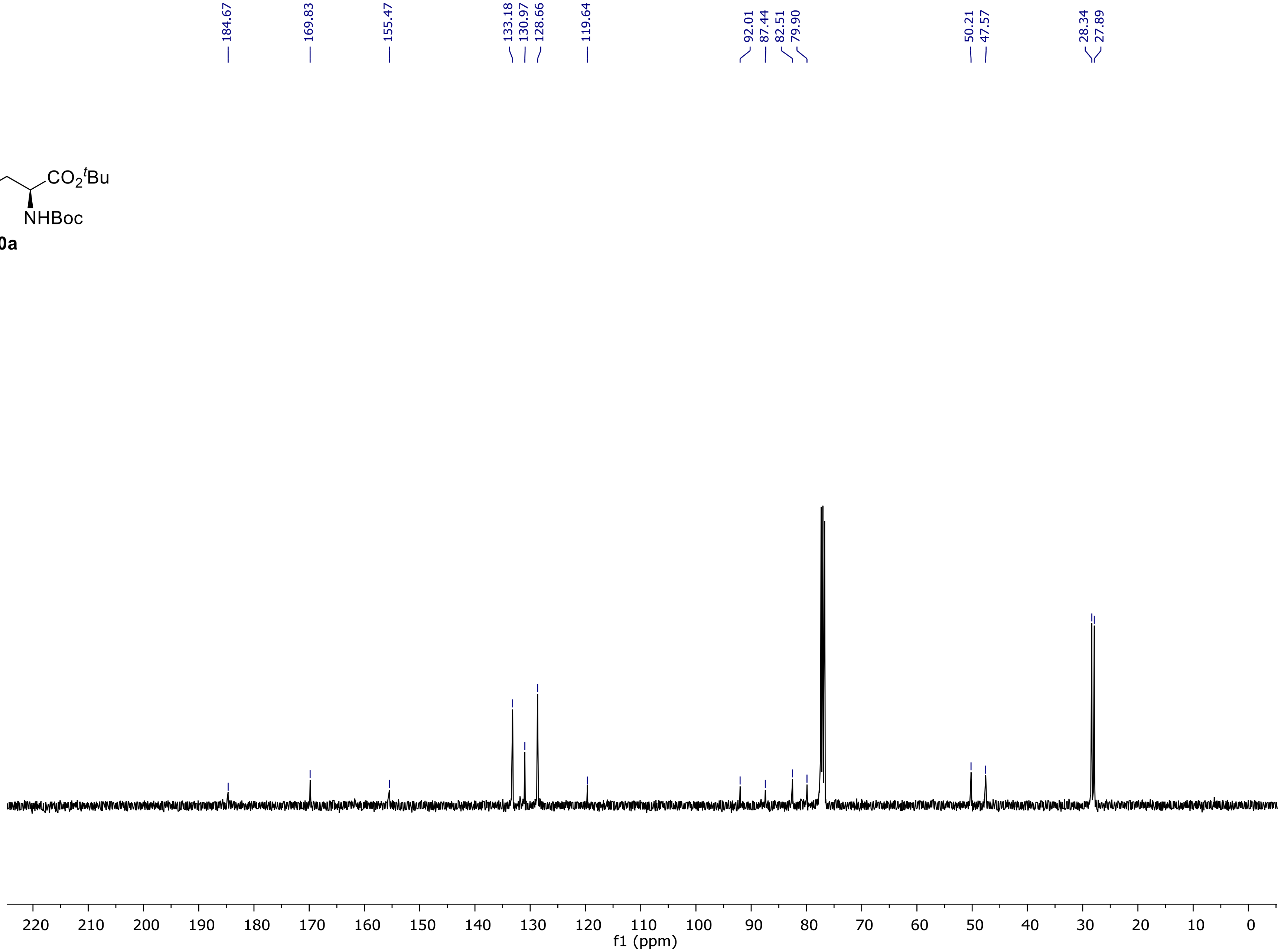

^{1}H NMR (400 MHz, $CDCl_3$)

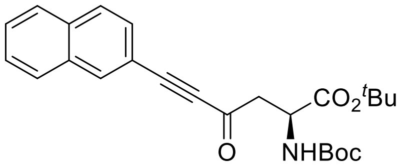

10b

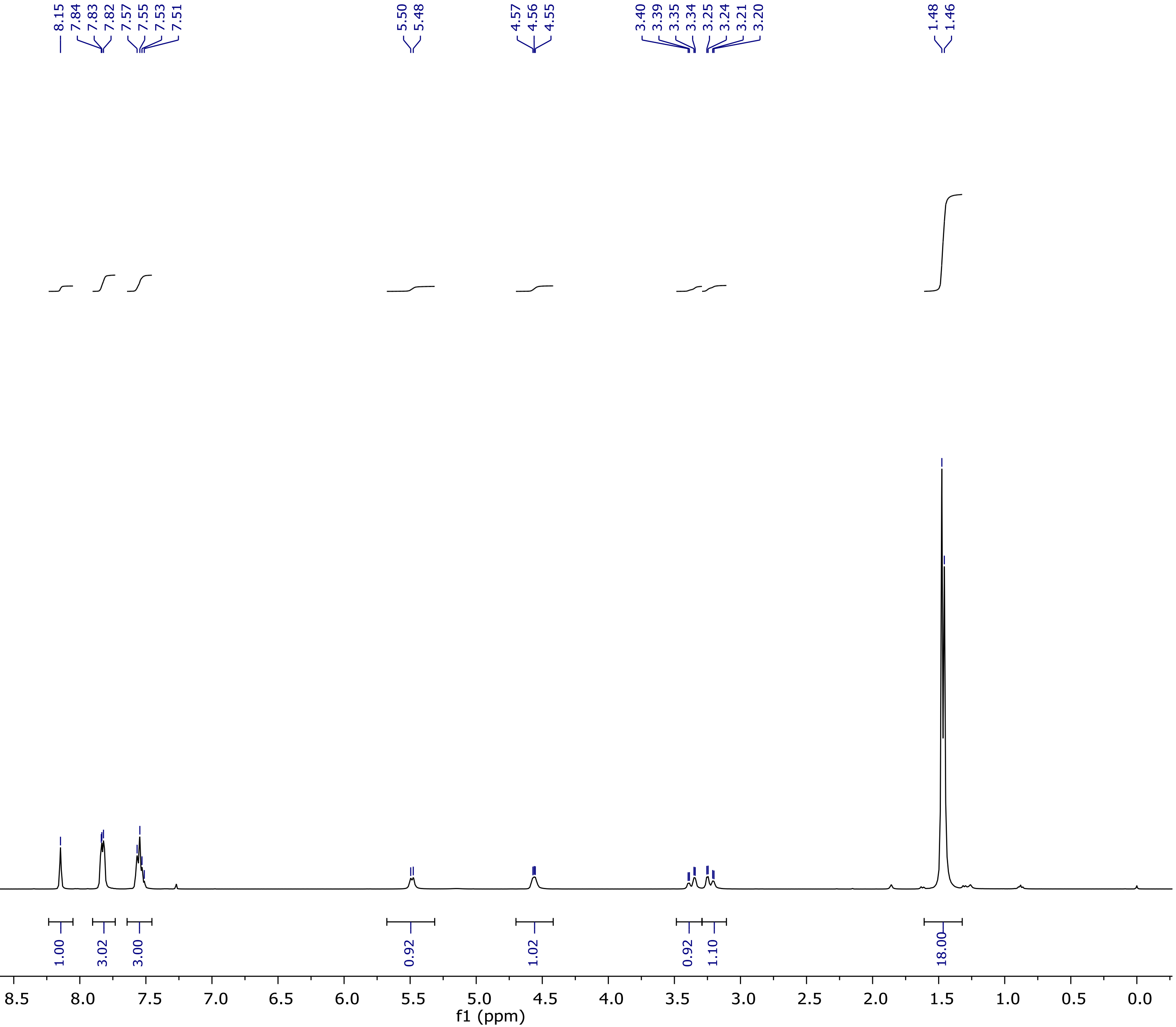

$^{13}C\{^1H\}$ NMR (101 MHz, $CDCl_3$)

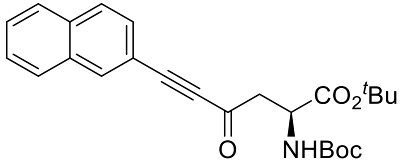

10b

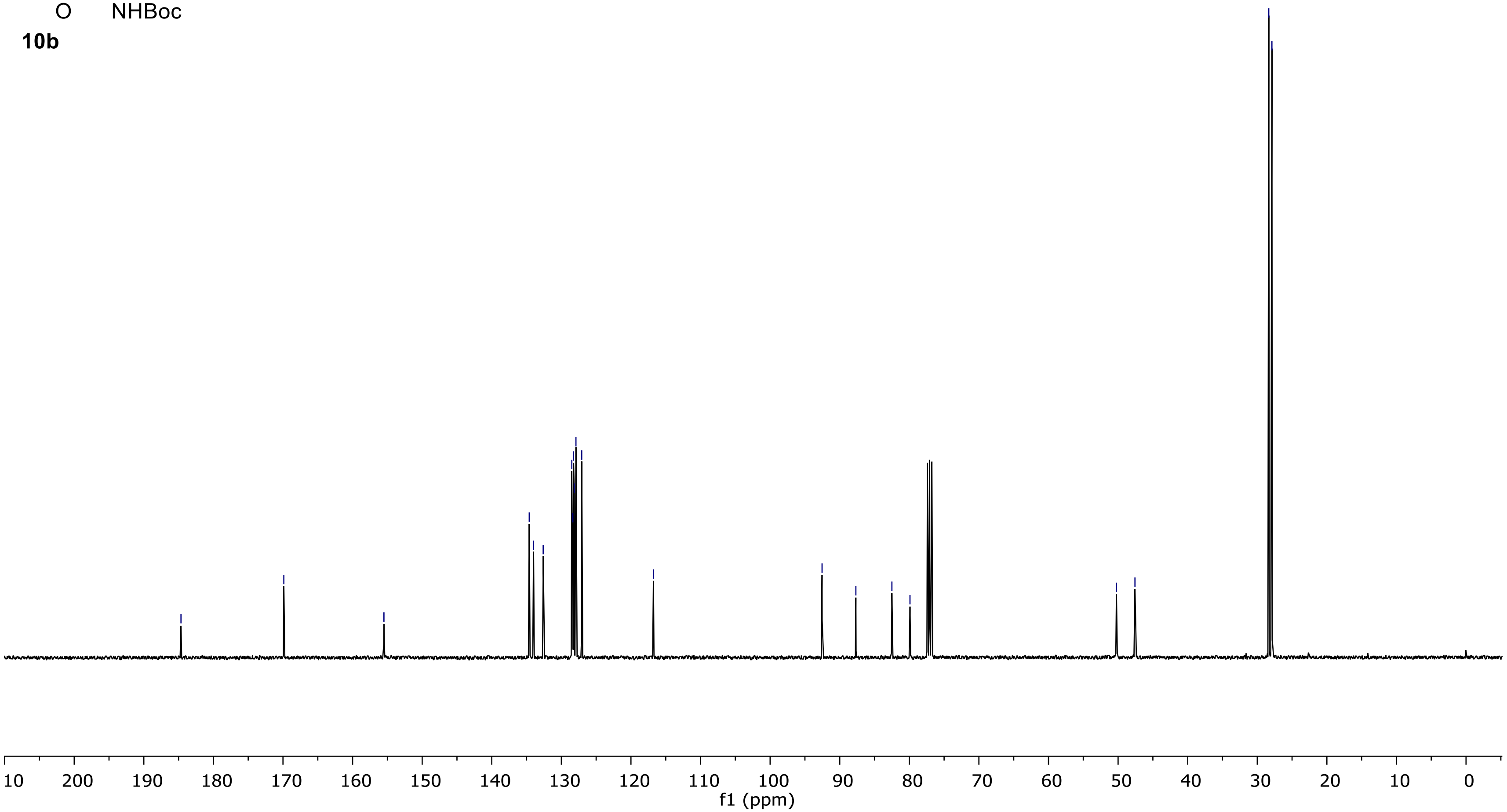

^{1}H NMR (400 MHz, $CDCl_3$)

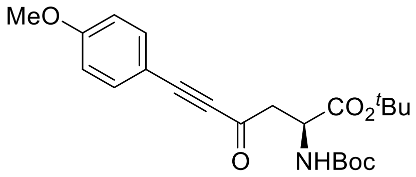

10c

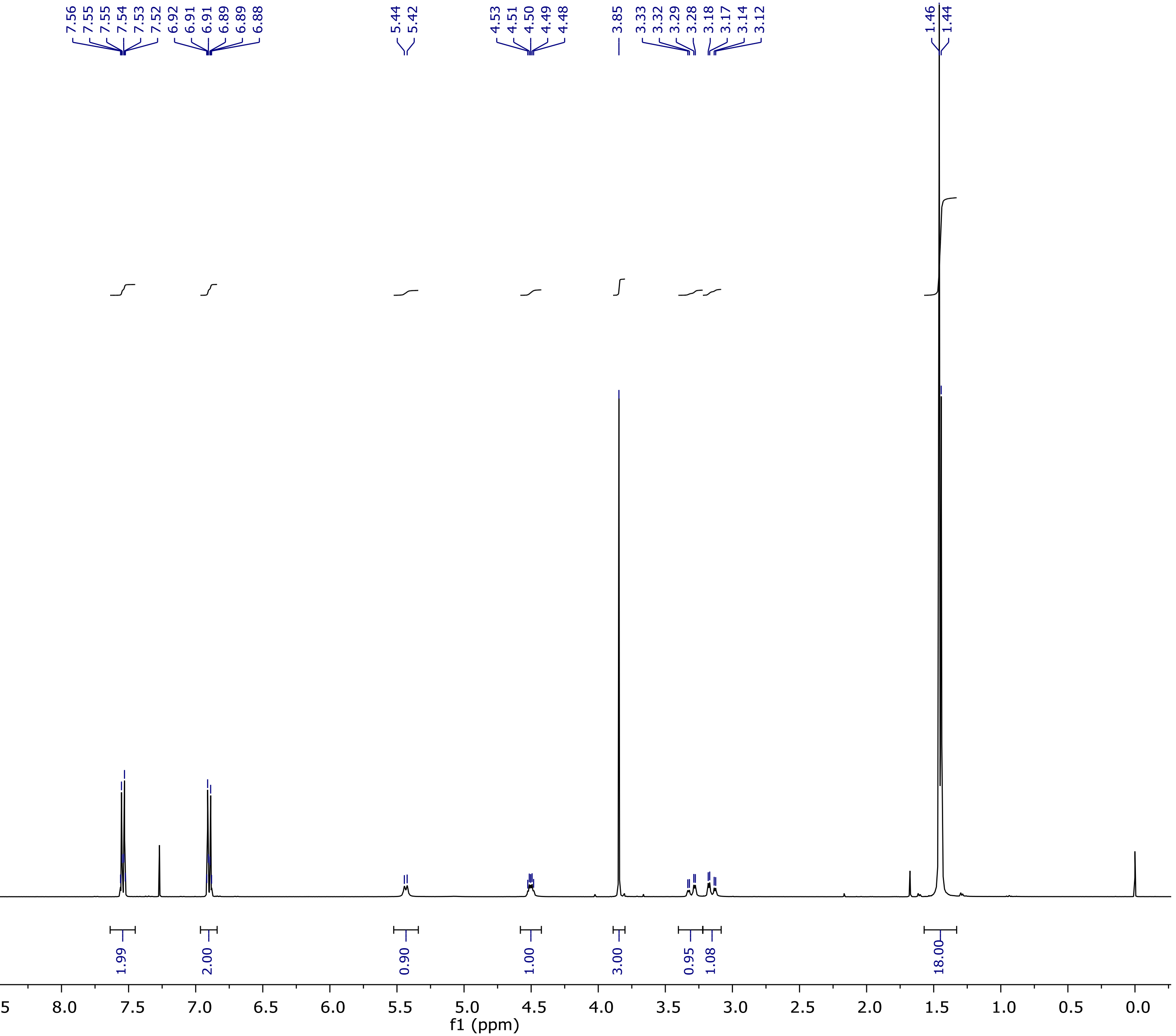

$^{13}C\{^{1}H\}$ NMR (101 MHz, $CDCl_3$)

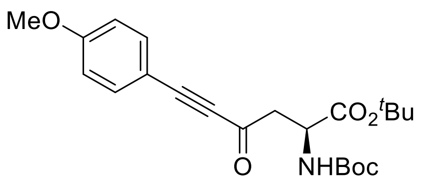

10c

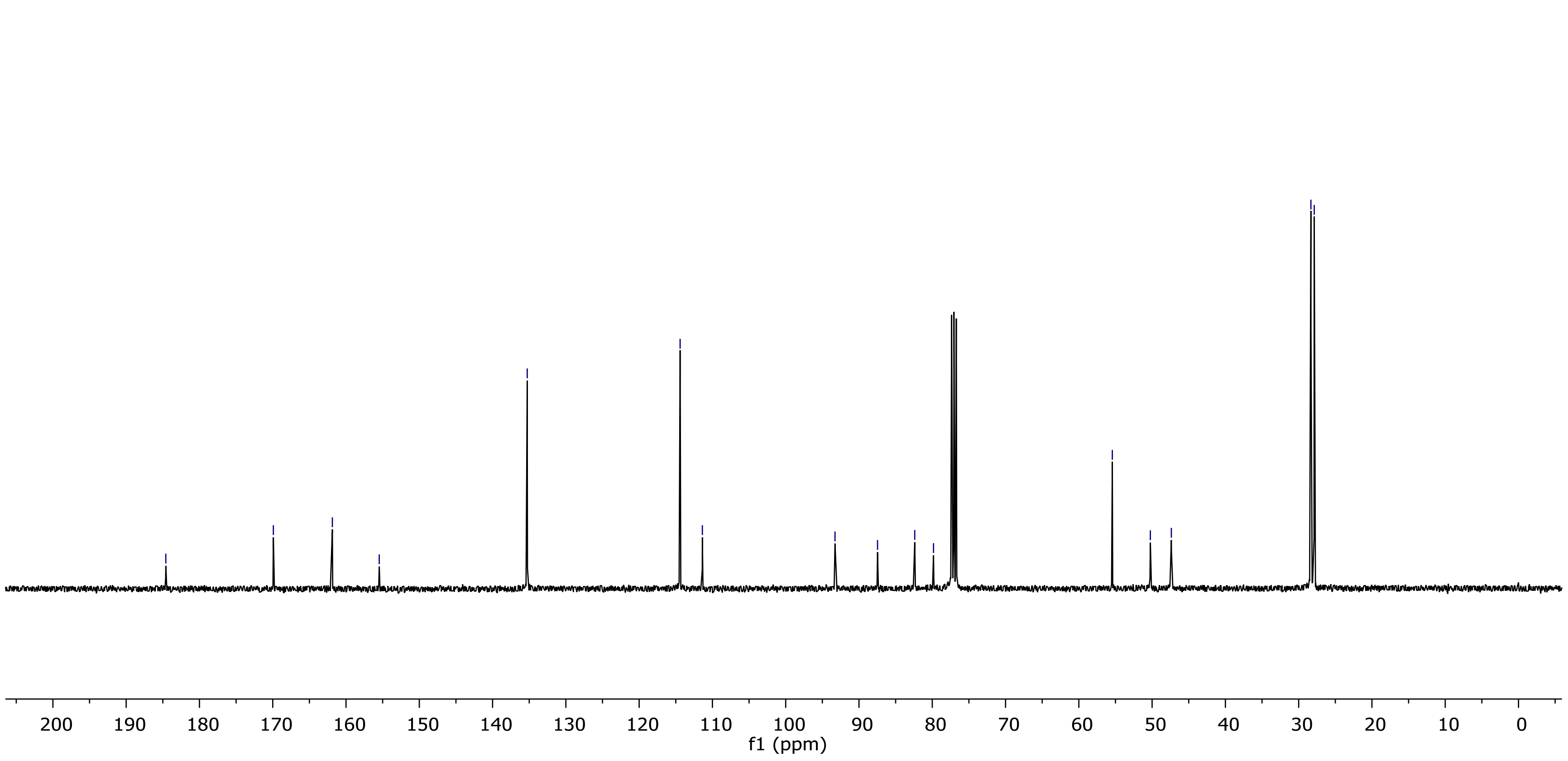

^{1}H NMR (400 MHz, $CDCl_3$)

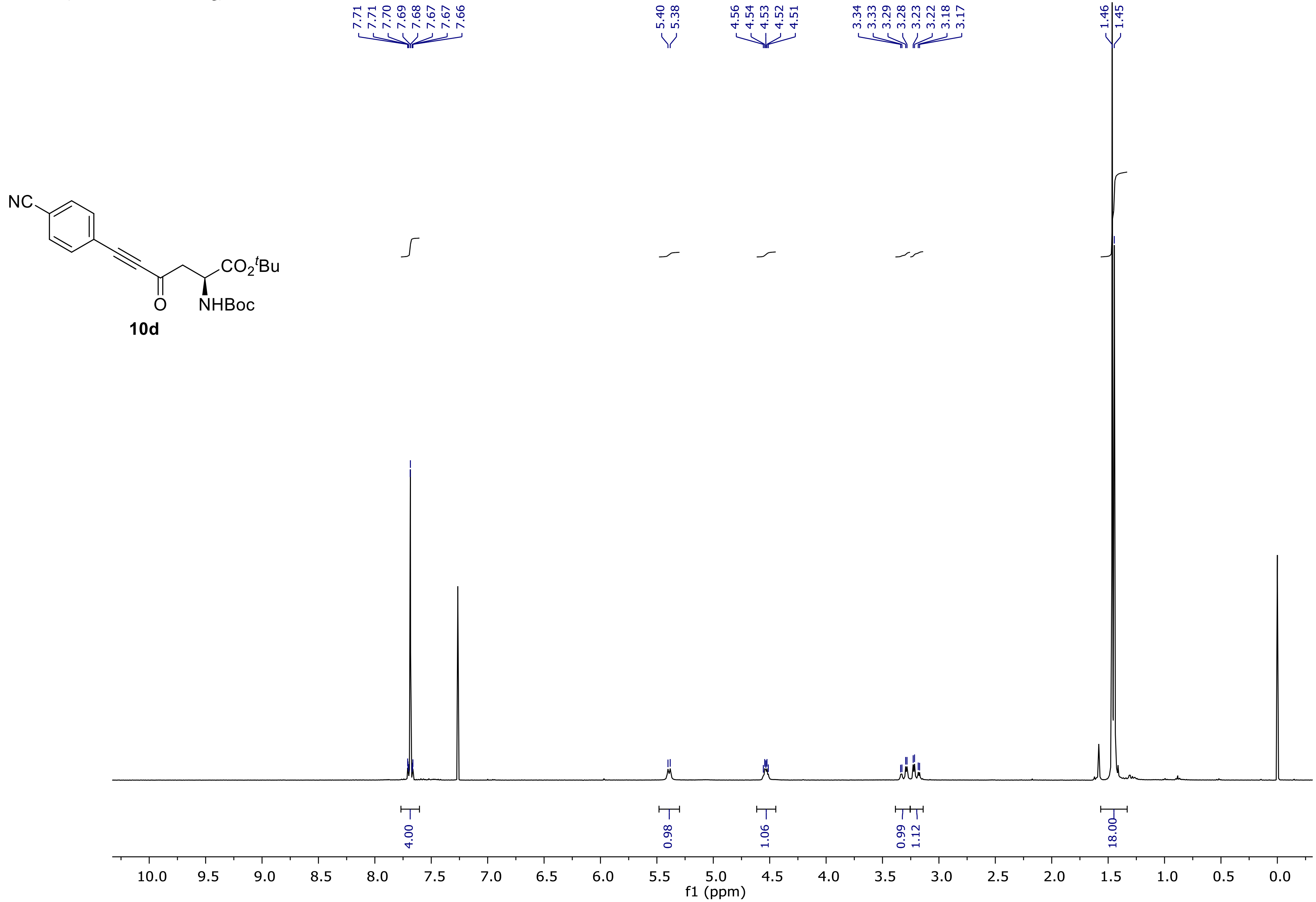

$^{13}C\{^{1}H\}$ NMR (101 MHz, $CDCl_3$)

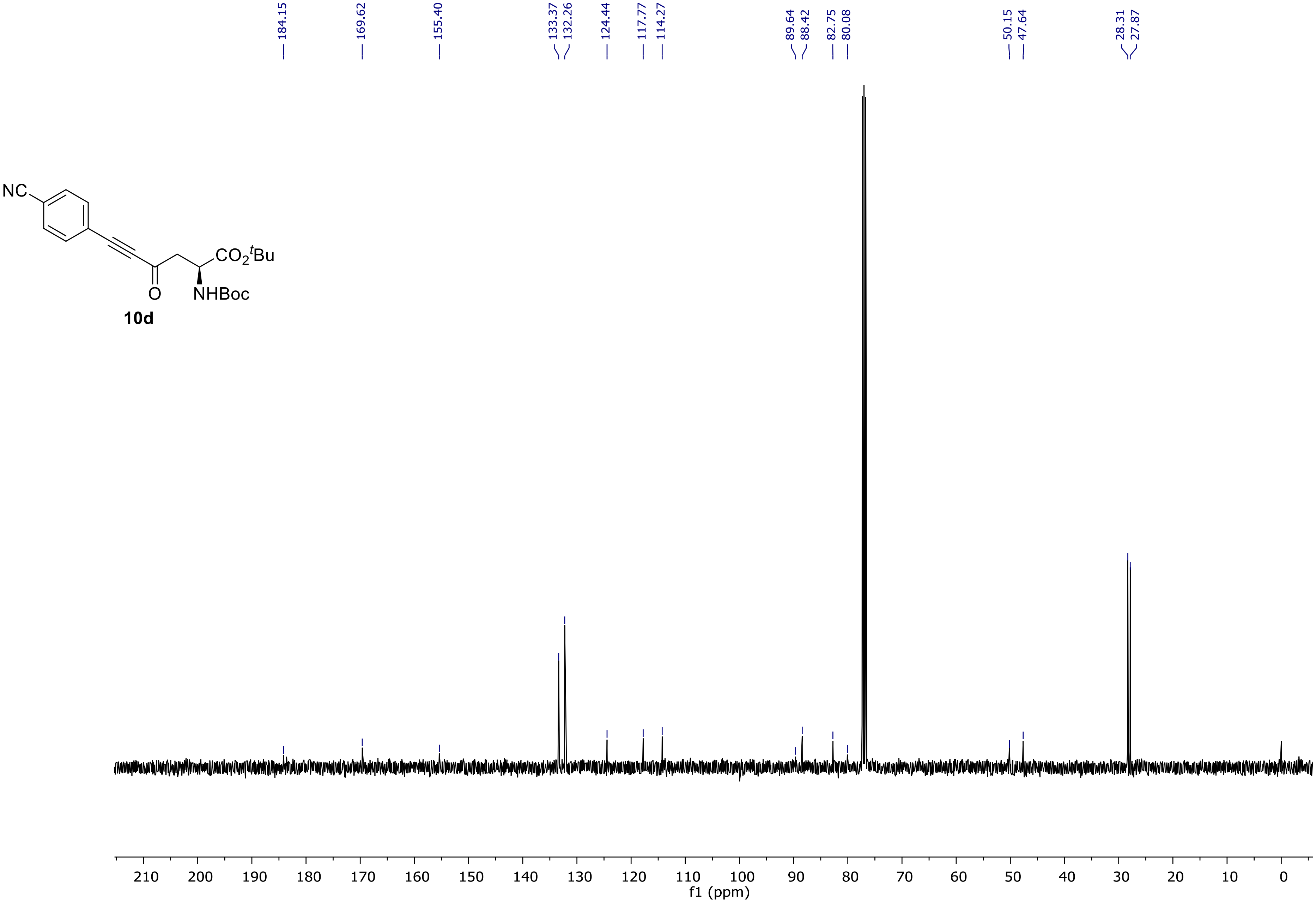

^1H NMR (400 MHz, $CDCl_3$)

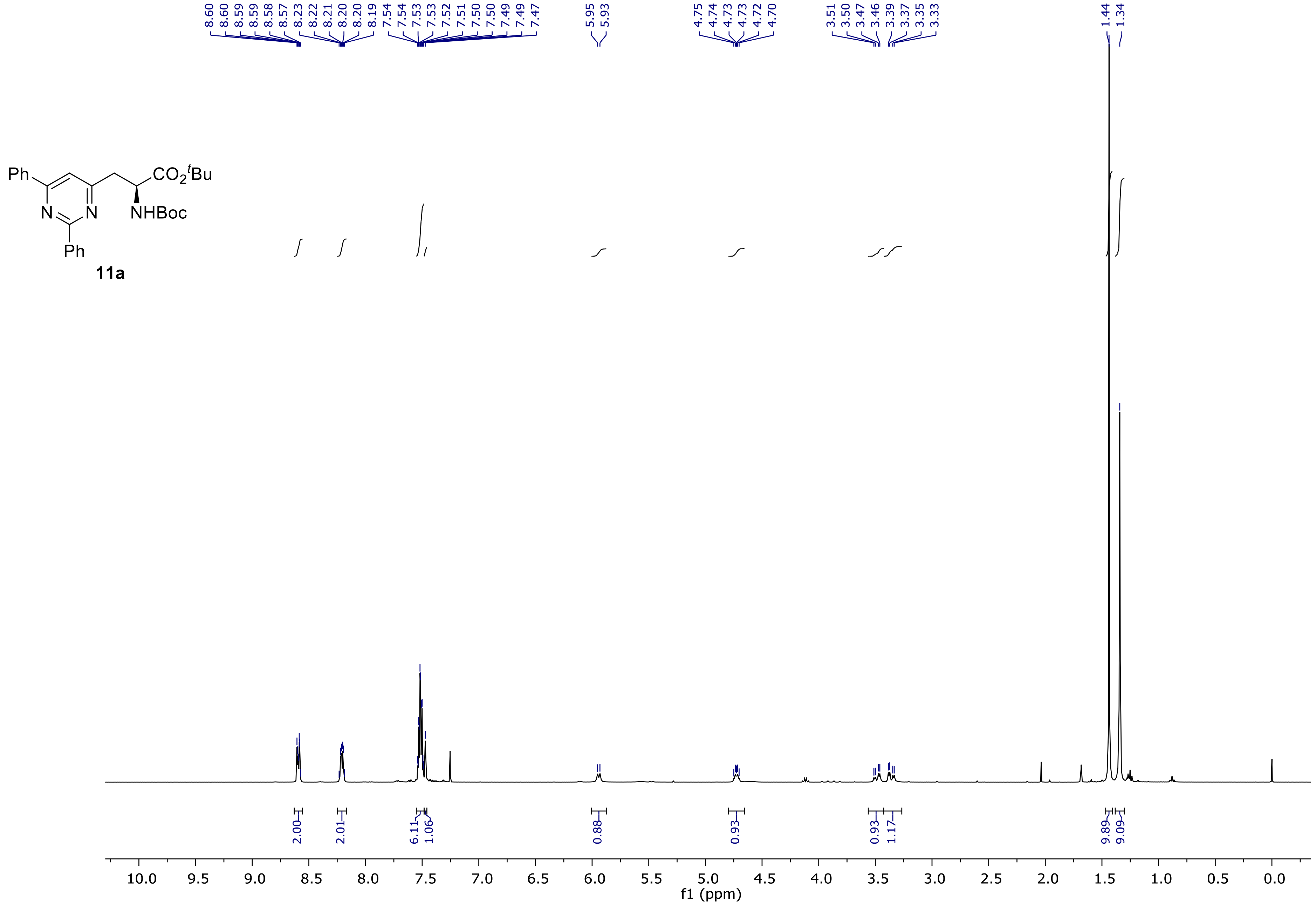

$^{13}C\{^{1}H\}$ NMR (101 MHz, $CDCl_3$)

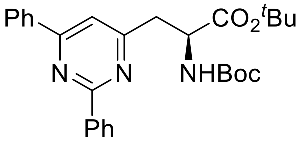

11a

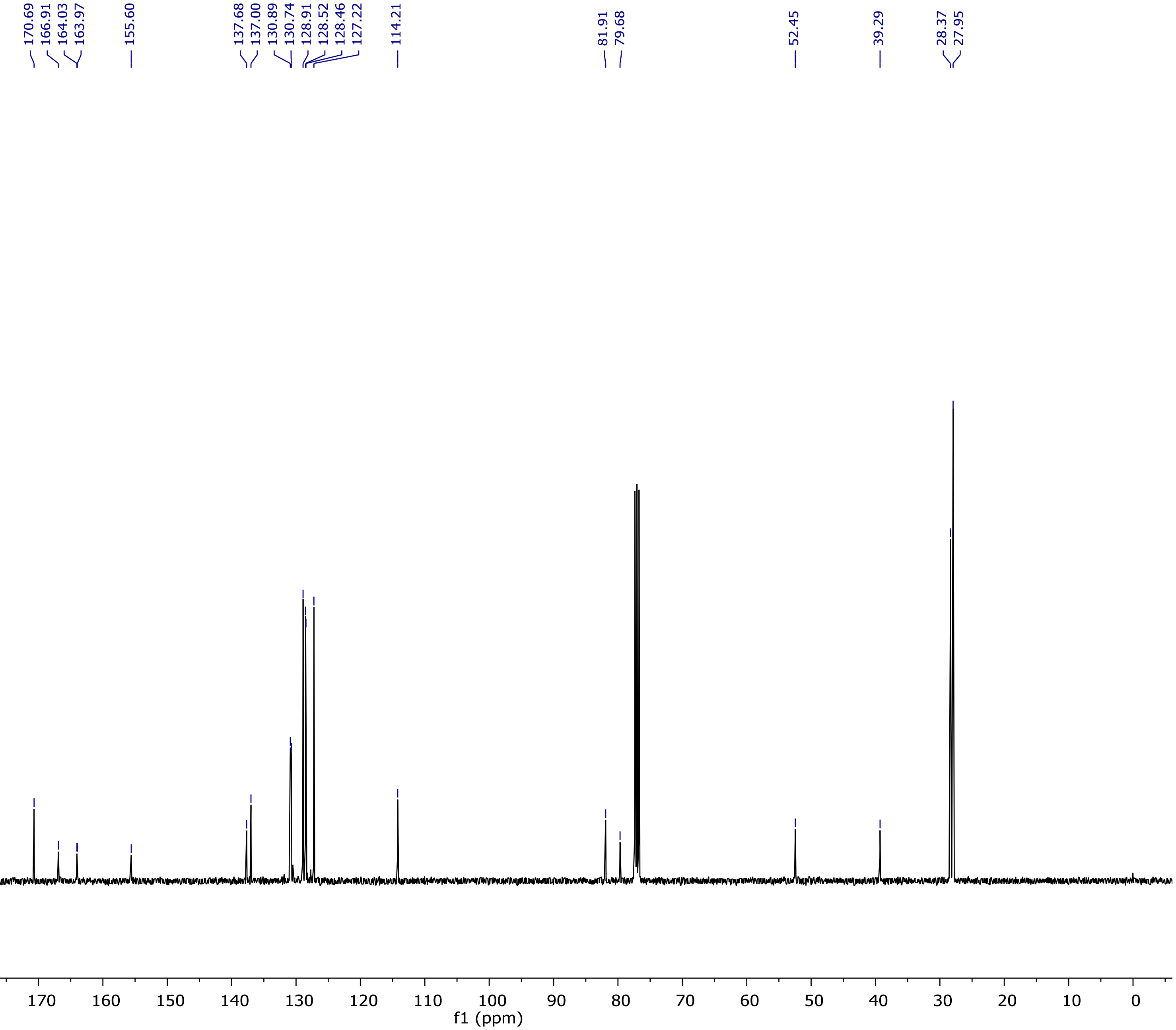

^{1}H NMR (400 MHz, $CDCl_3$)

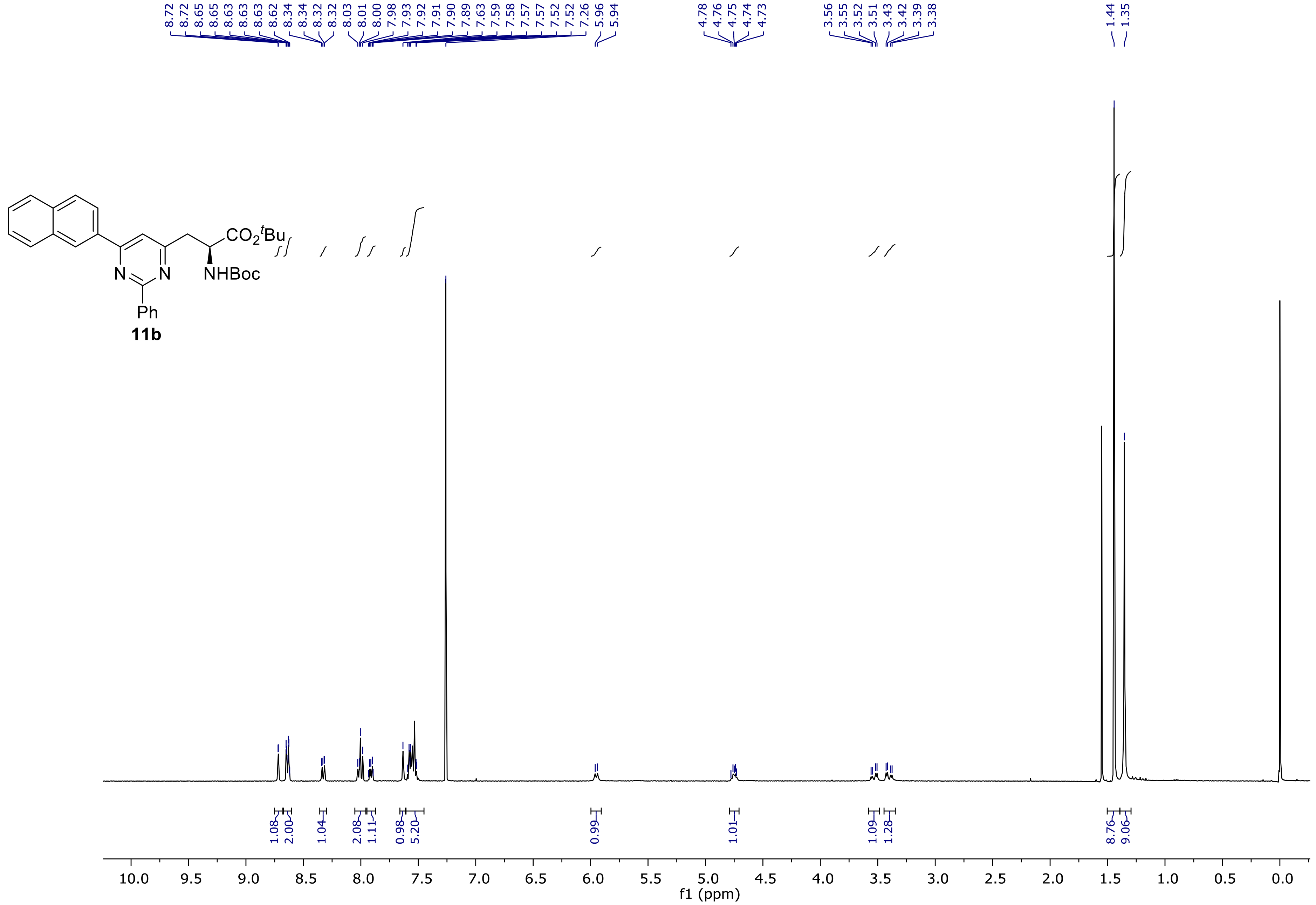

$^{13}C\{^{1}H\}$ **NMR (101 MHz, CDCl$_3$)**

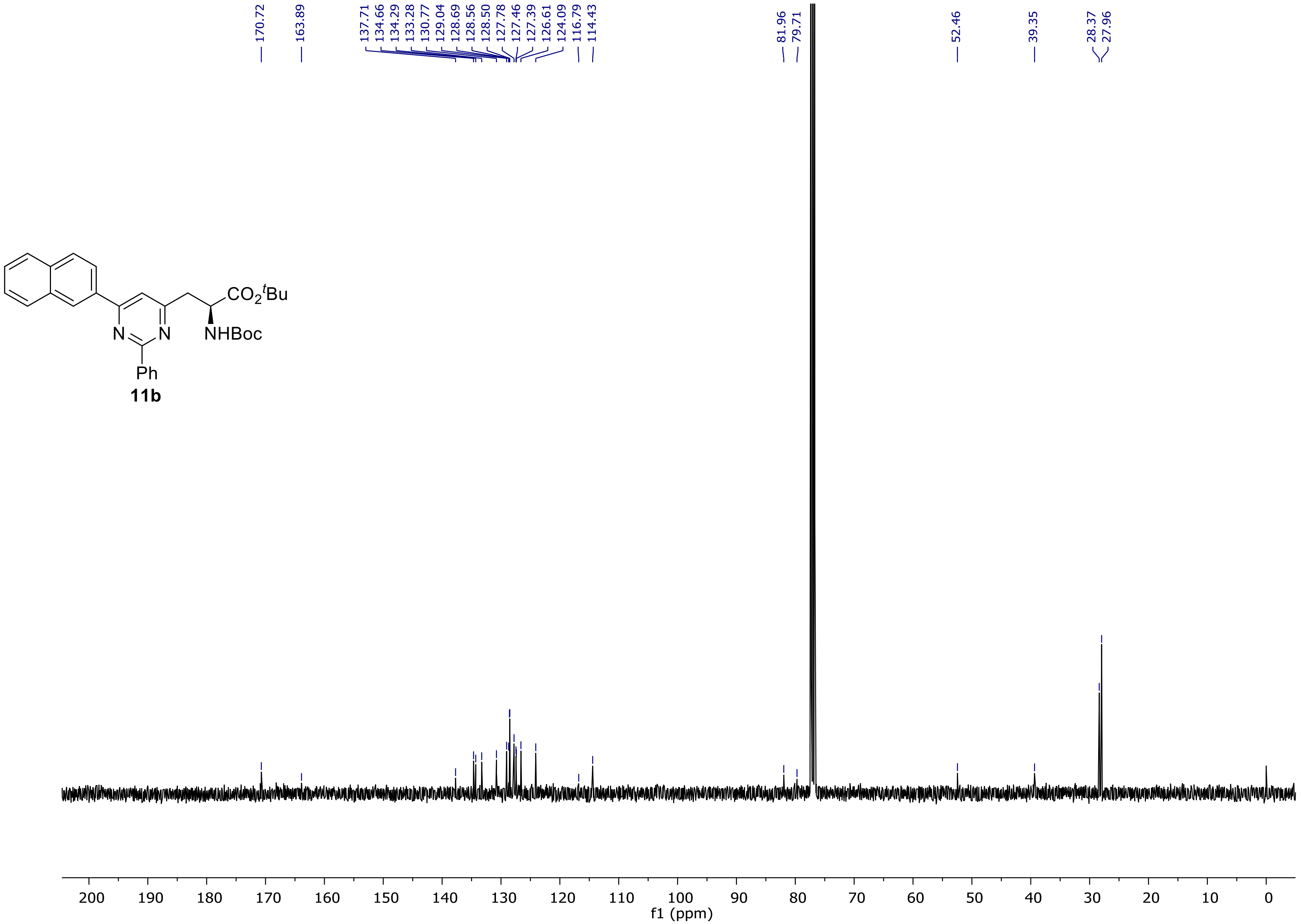

11b

^{1}H NMR (400 MHz, $CDCl_3$)

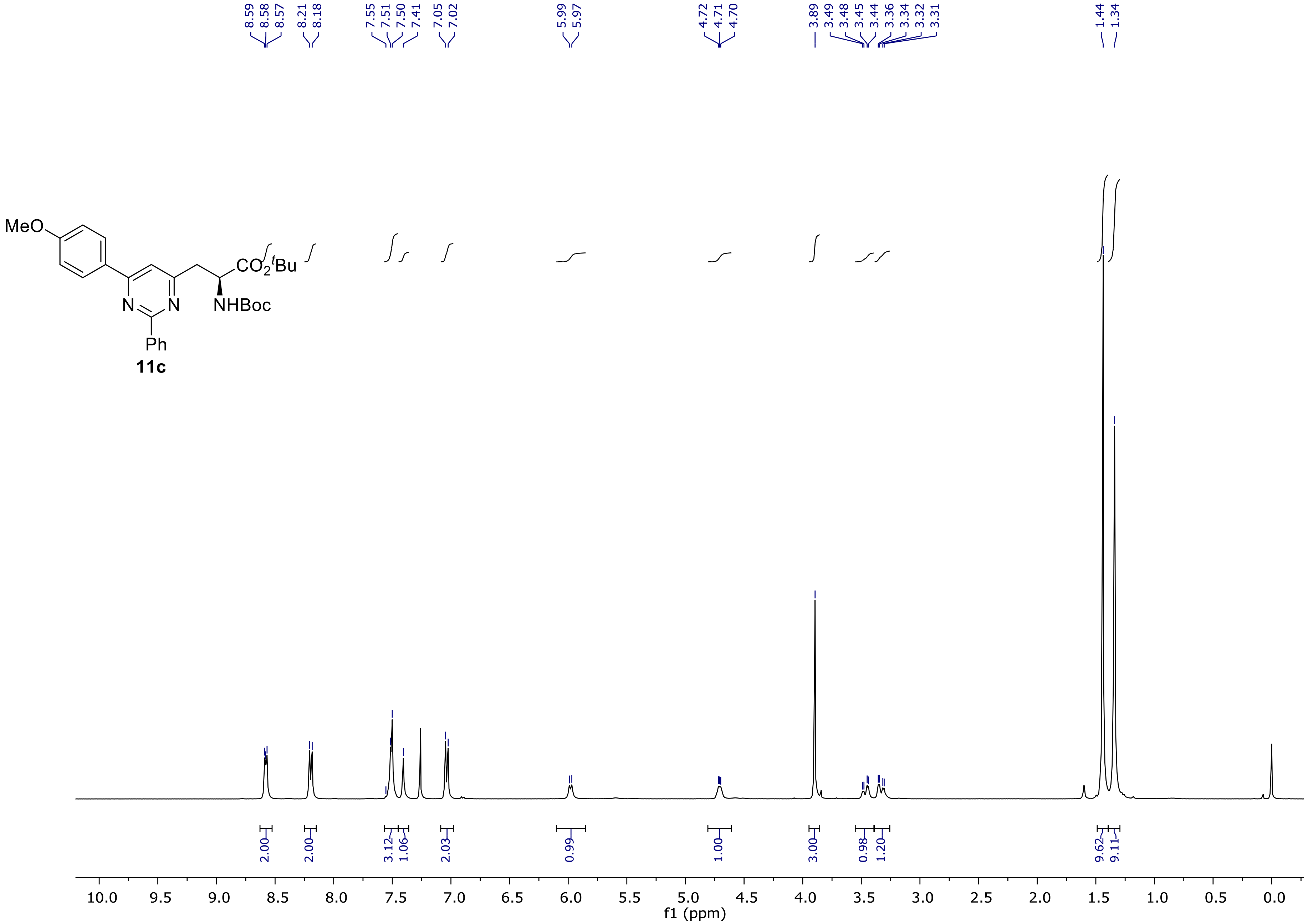

$^{13}C\{^{1}H\}$ NMR (101 MHz, $CDCl_3$)

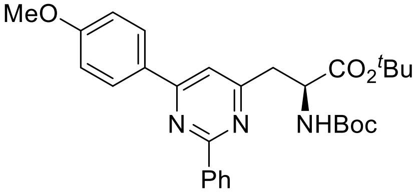

11c

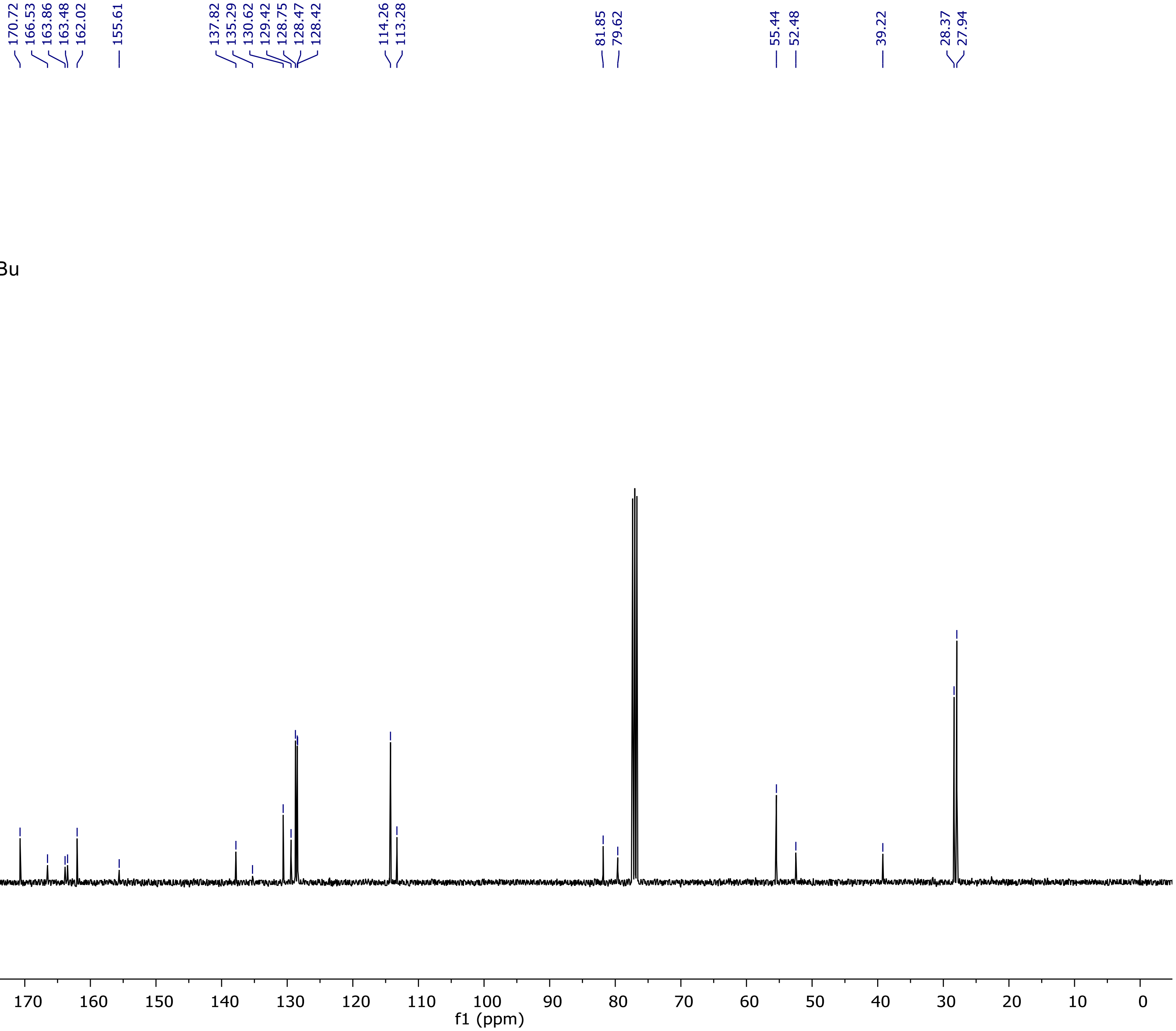

^{1}H NMR (400 MHz, $CDCl_3$)

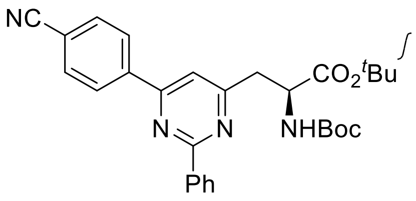

11d

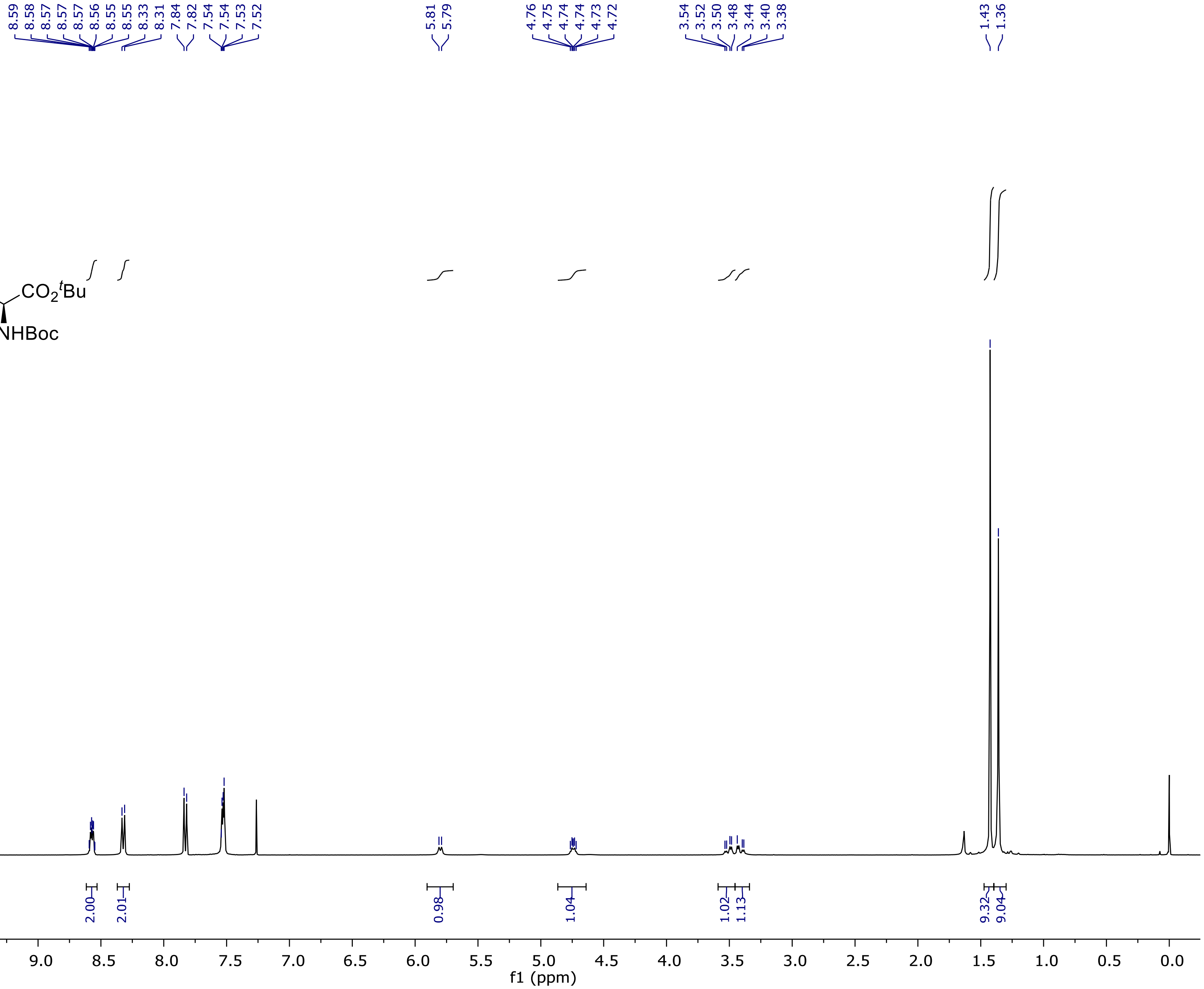

$^{13}C\{^{1}H\}$ NMR (101 MHz, $CDCl_3$)

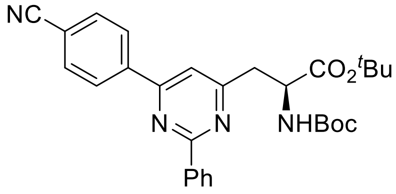

11d

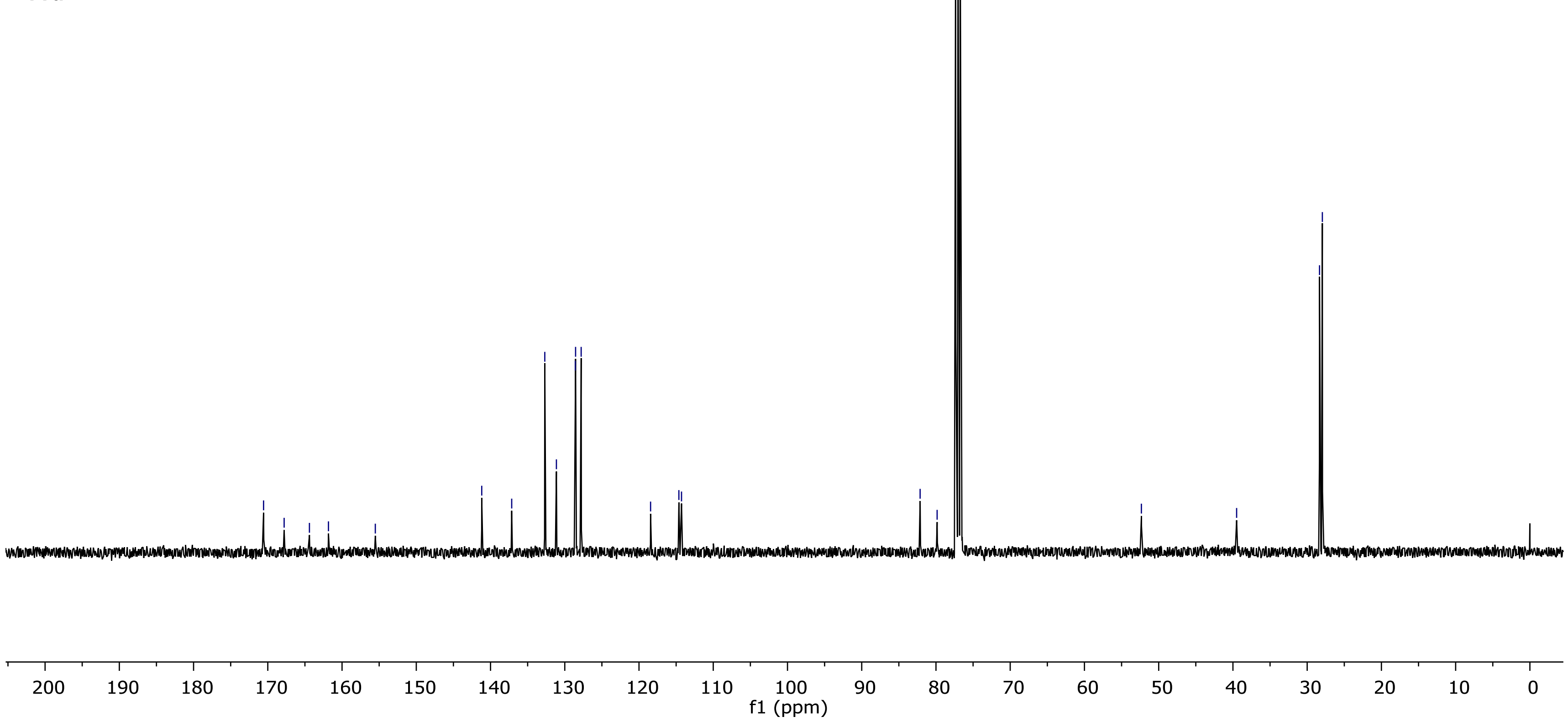

^{1}H NMR (400 MHz, CDCl$_3$)

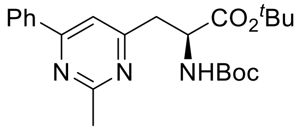

11e

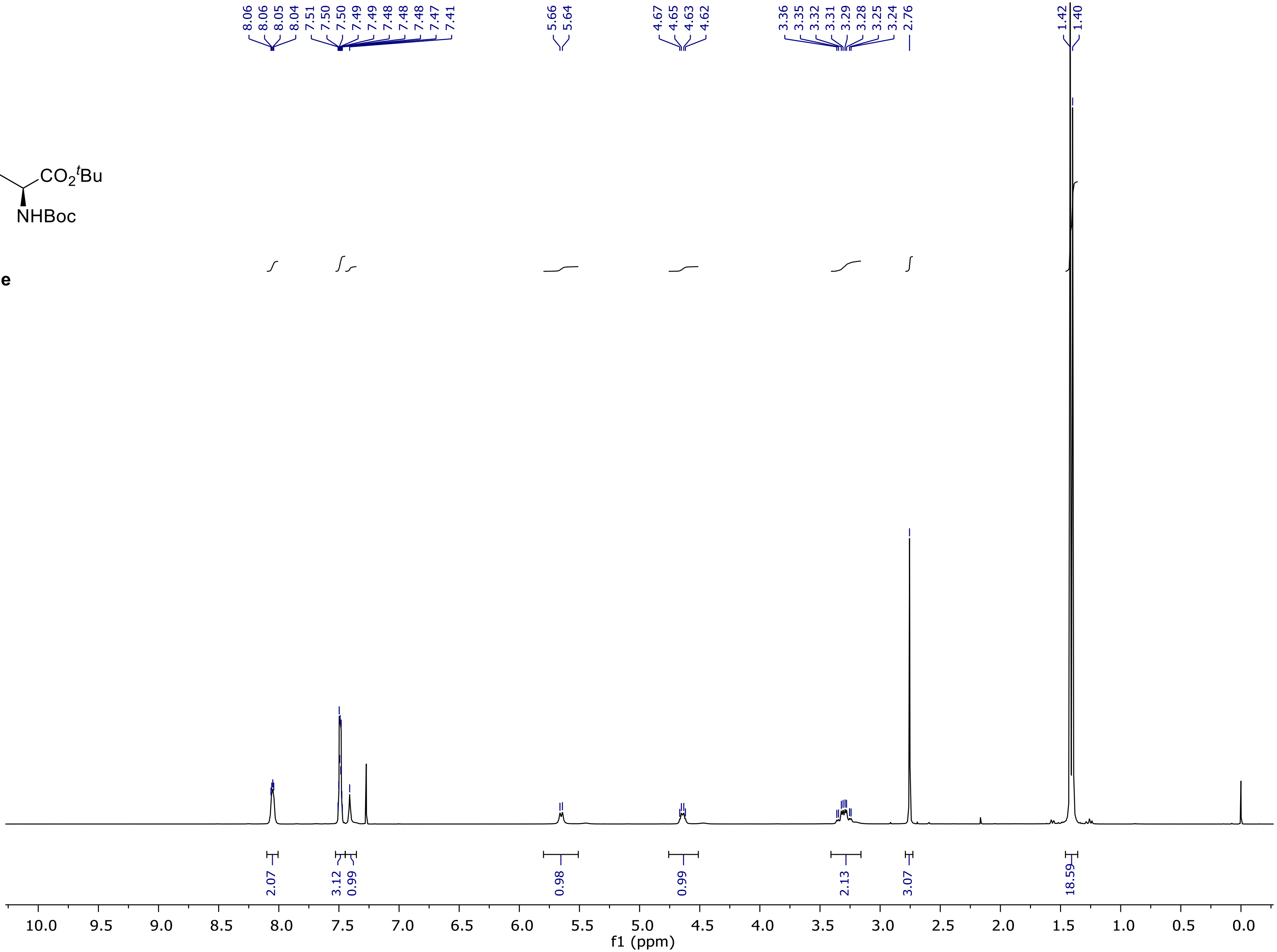

$^{13}C\{^{1}H\}$ NMR (101 MHz, $CDCl_3$)

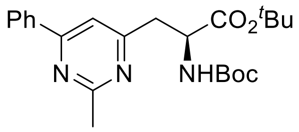

11e

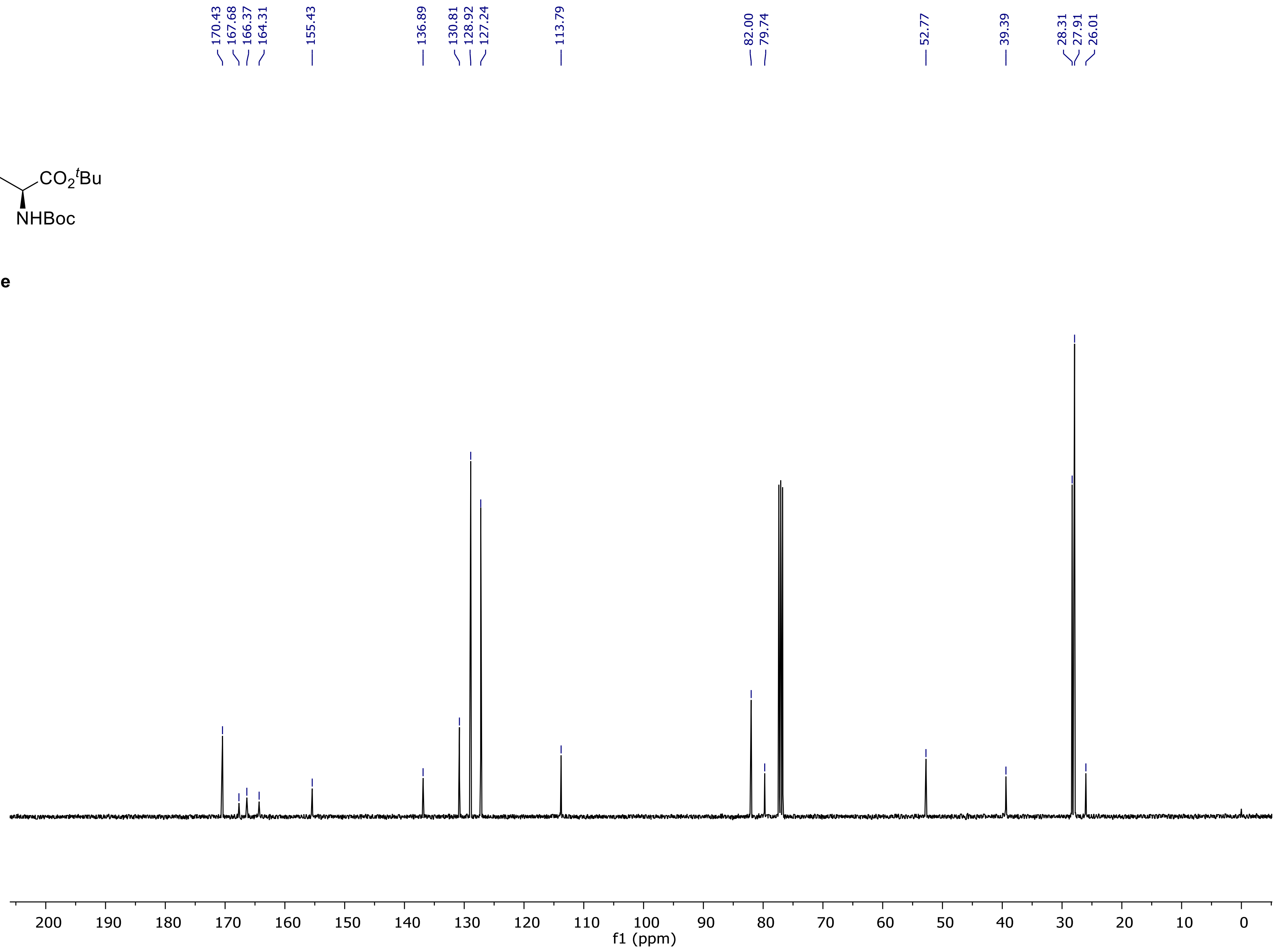

^{1}H NMR (400 MHz, $CDCl_3$)

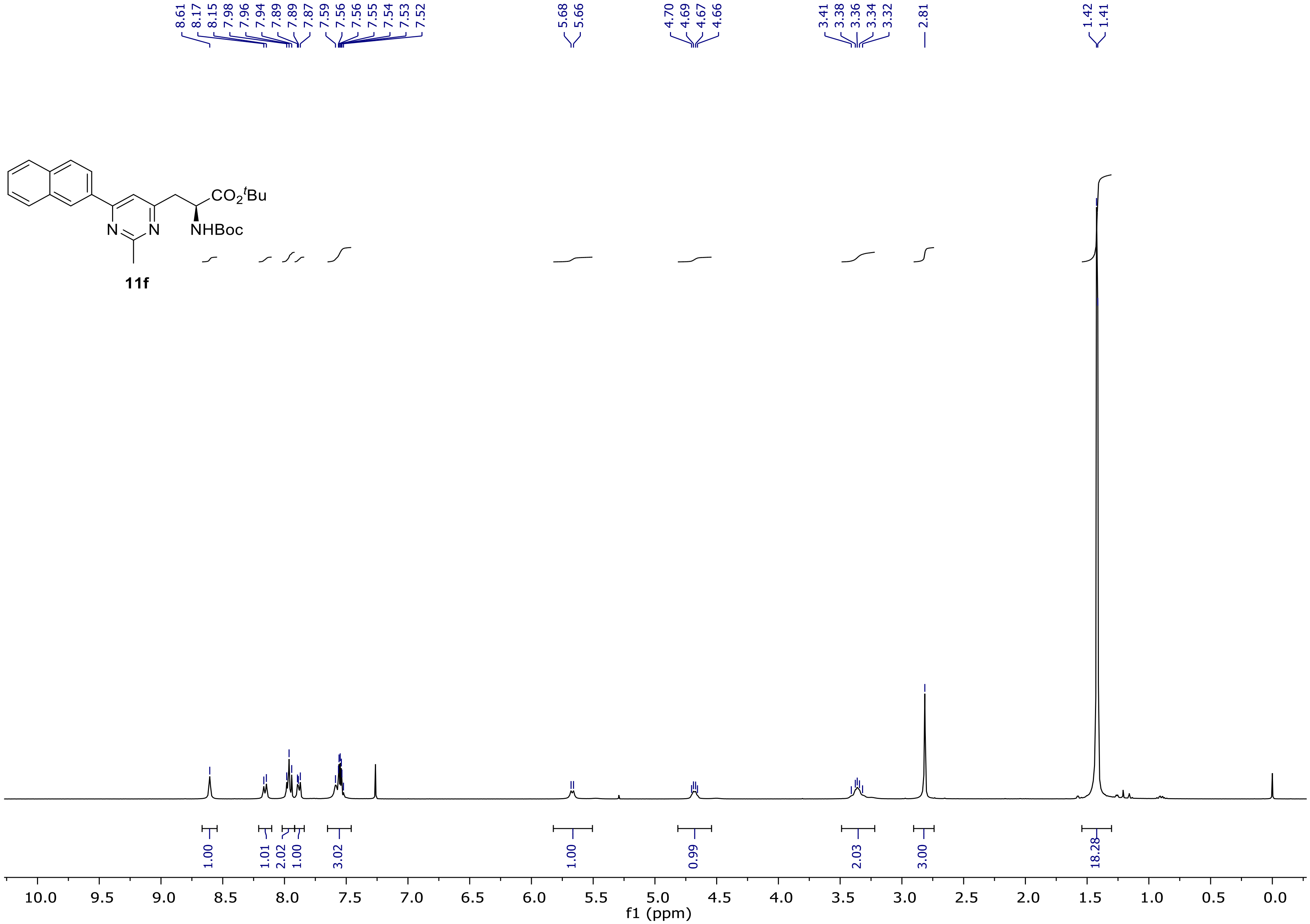

$^{13}C\{^{1}H\}$ **NMR (101 MHz, $CDCl_3$)**

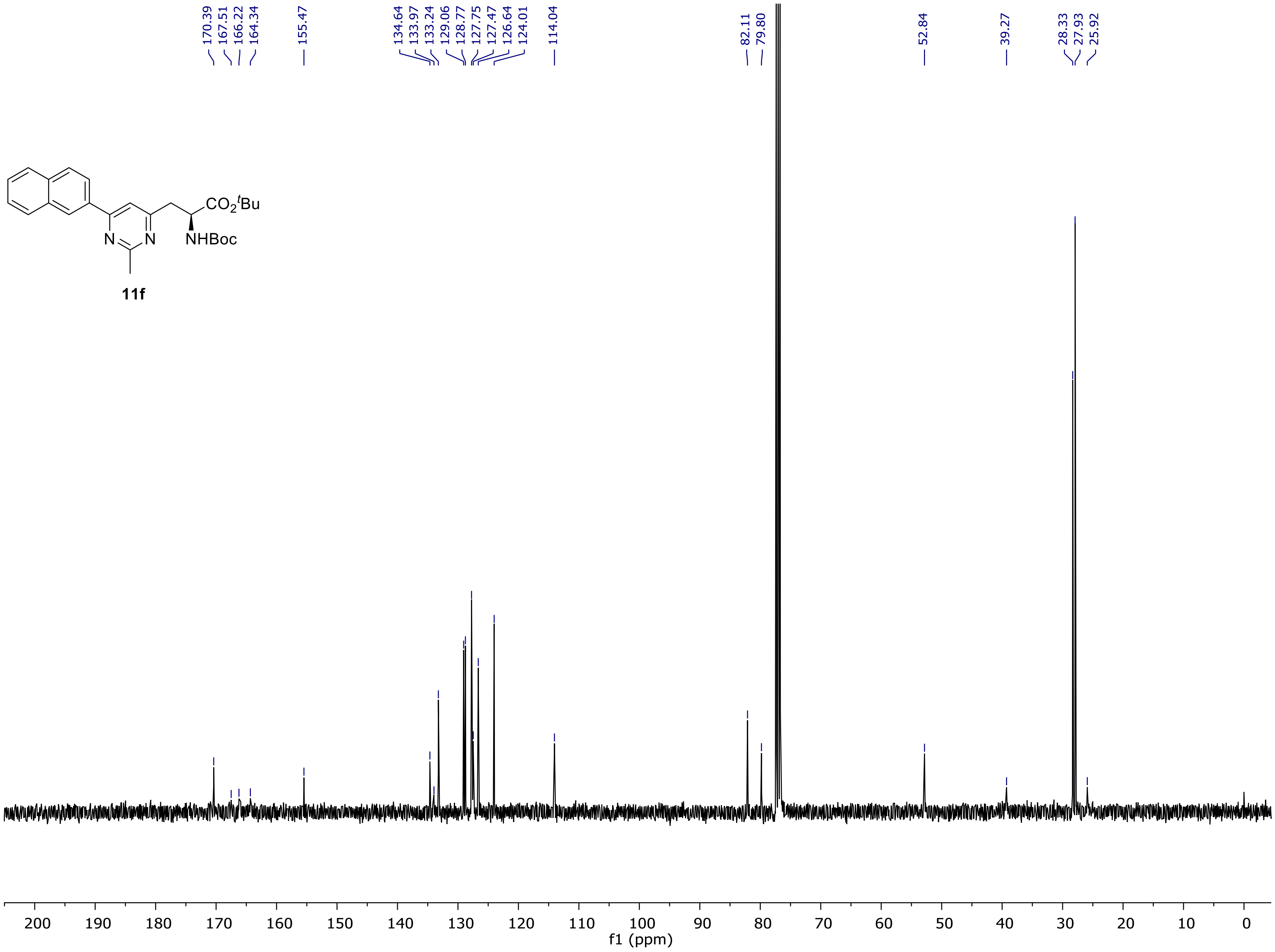

11f

^{1}H NMR (400 MHz, $CDCl_3$)

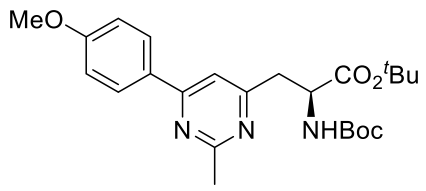

11g

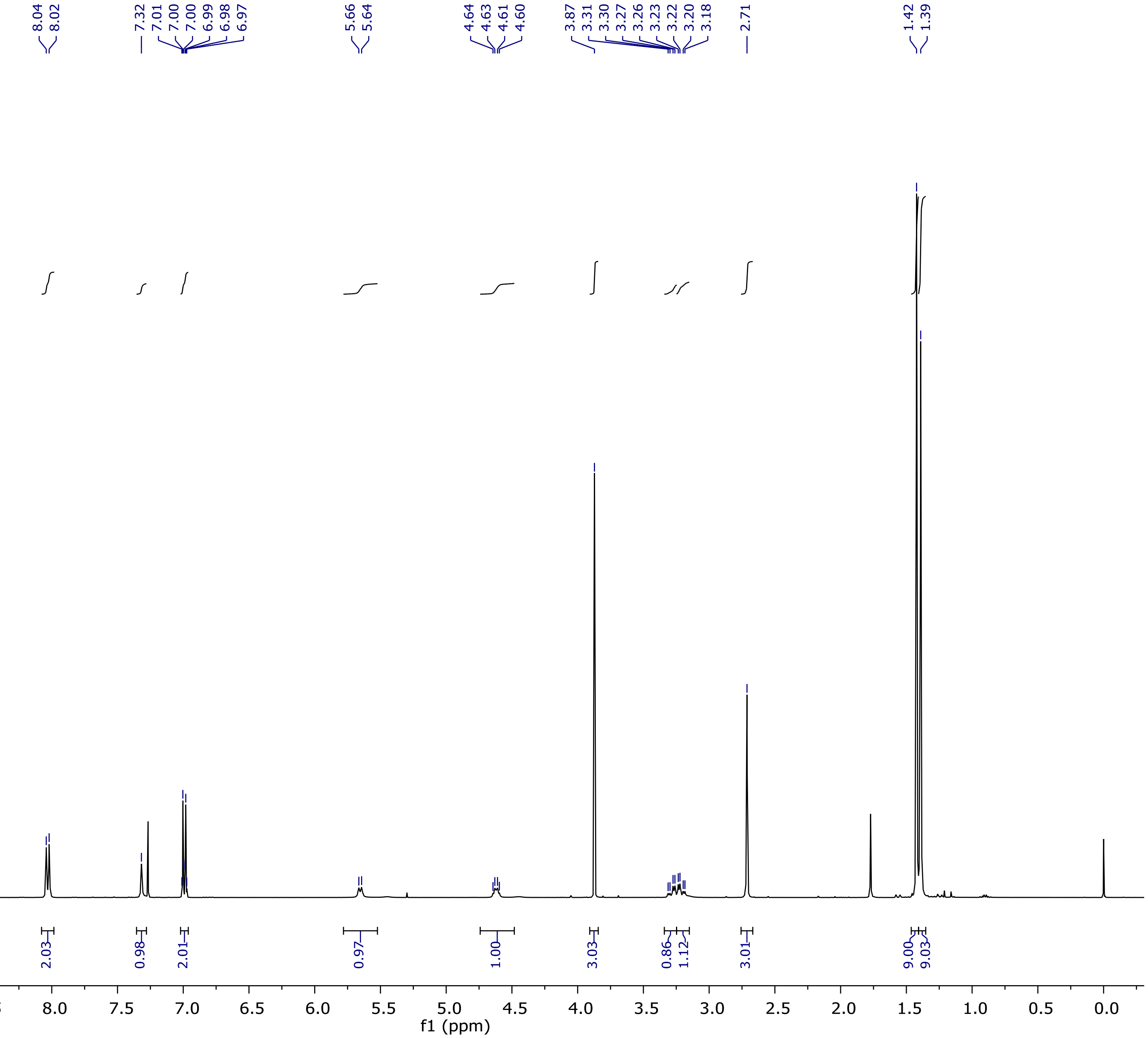

$^{13}C\{^{1}H\}$ NMR (101 MHz, $CDCl_3$)

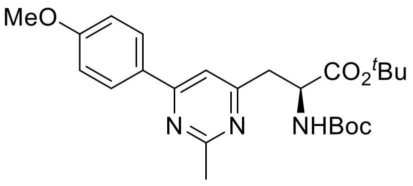

11g

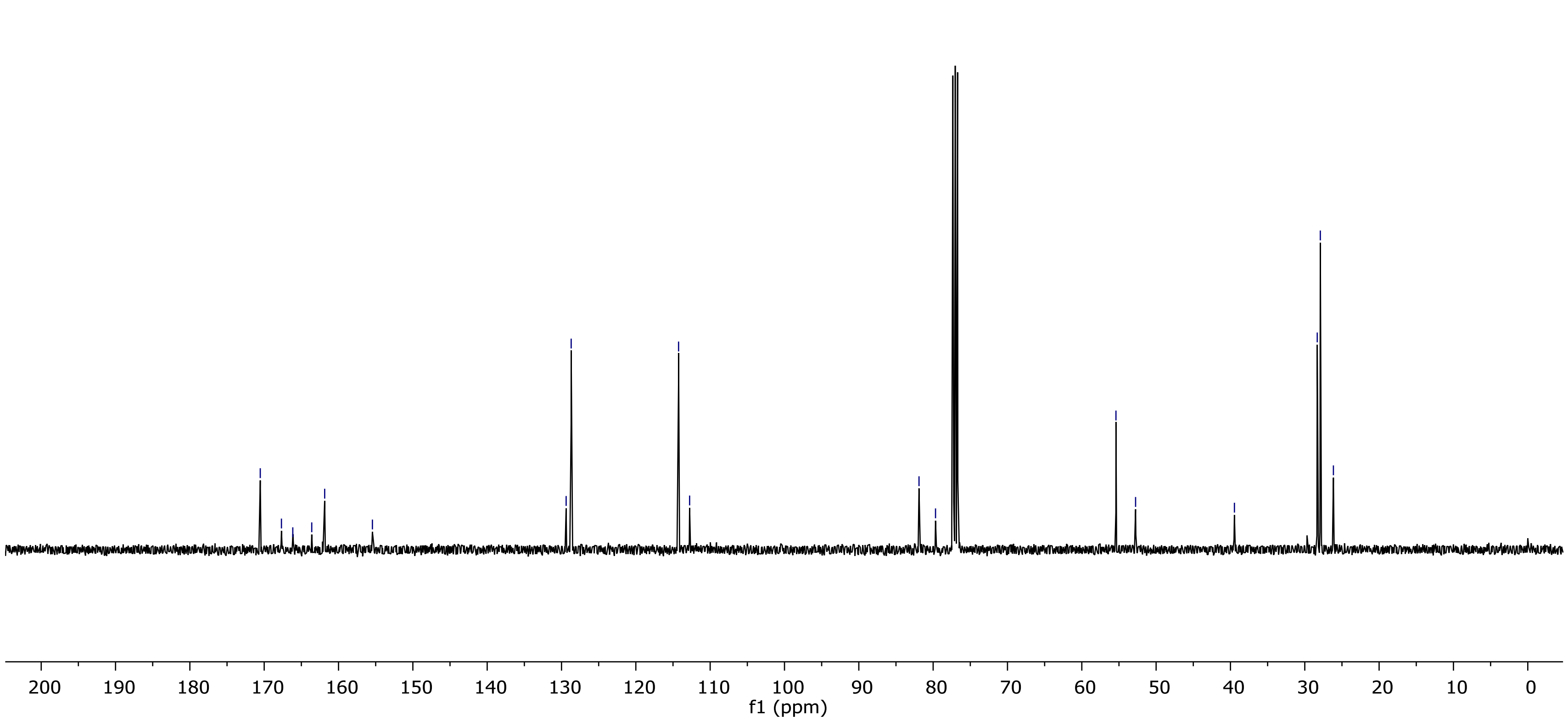

^{1}H NMR (400 MHz, $CDCl_3$)

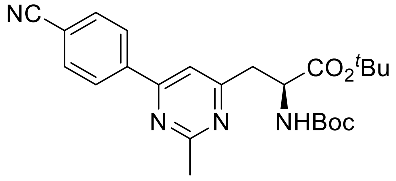

11h

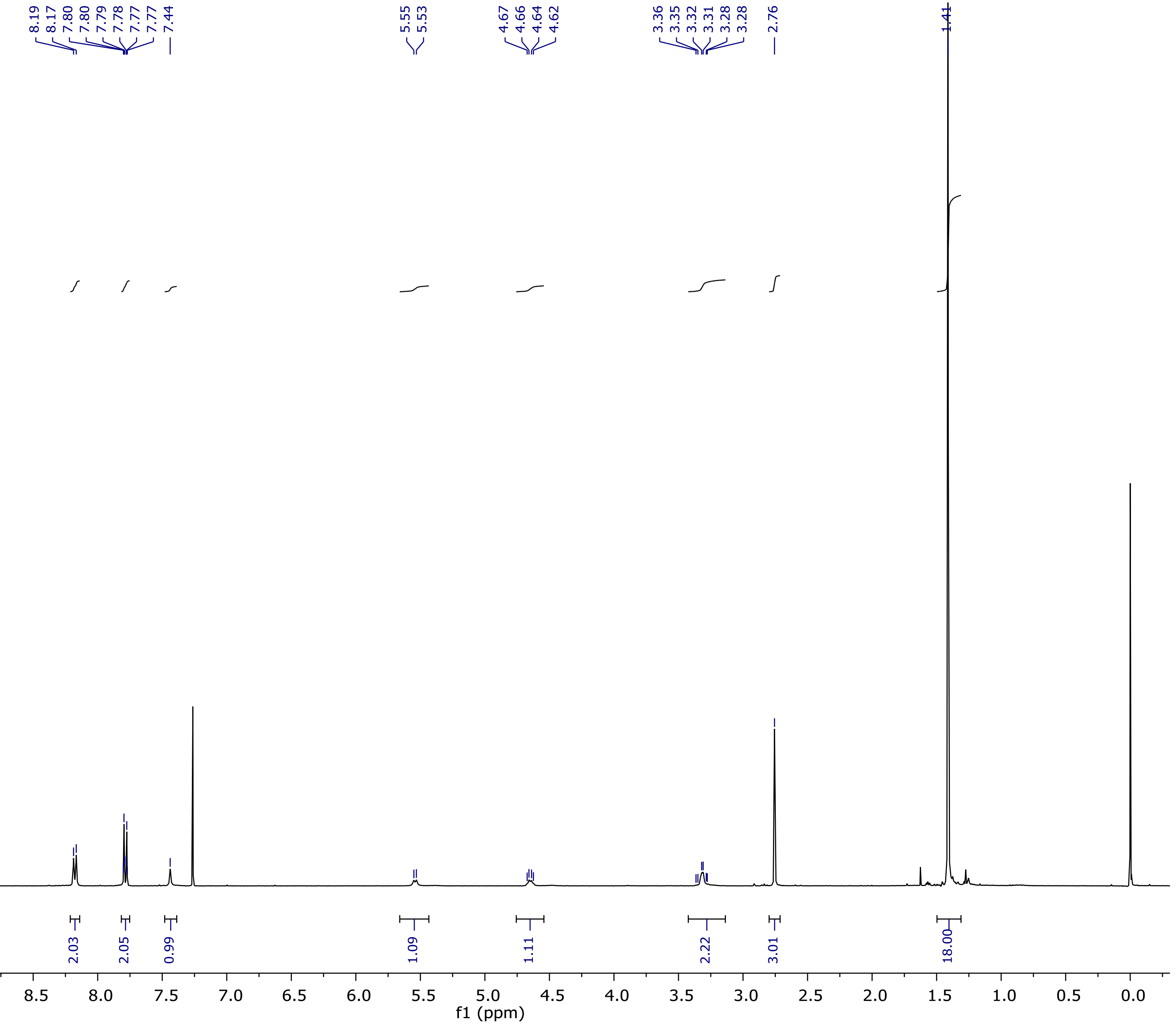

$^{13}C\{^1H\}$ NMR (101 MHz, CDCl$_3$)

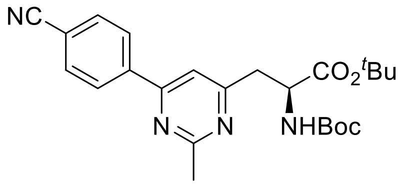

11h

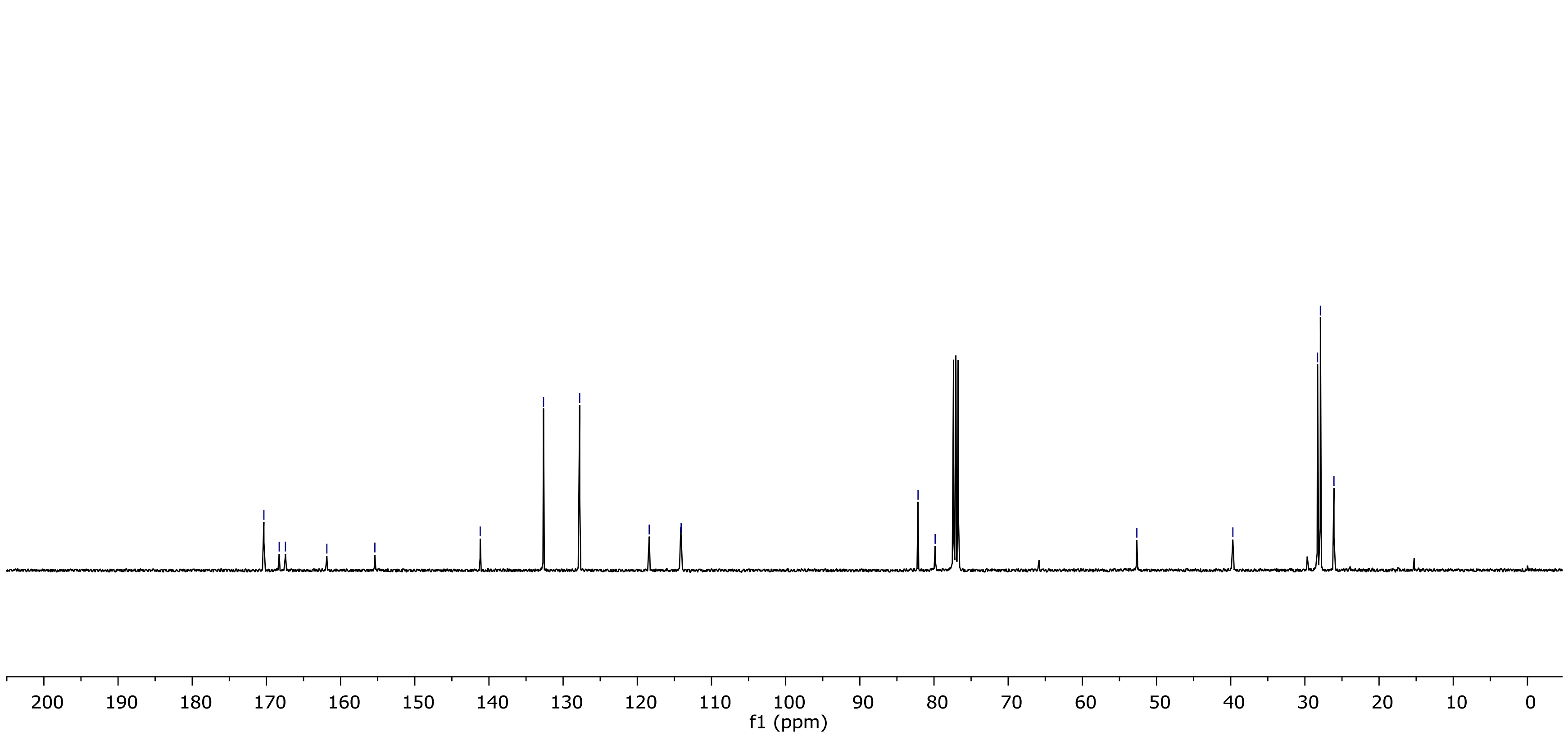

^{1}H NMR (400 MHz, $CDCl_3$)

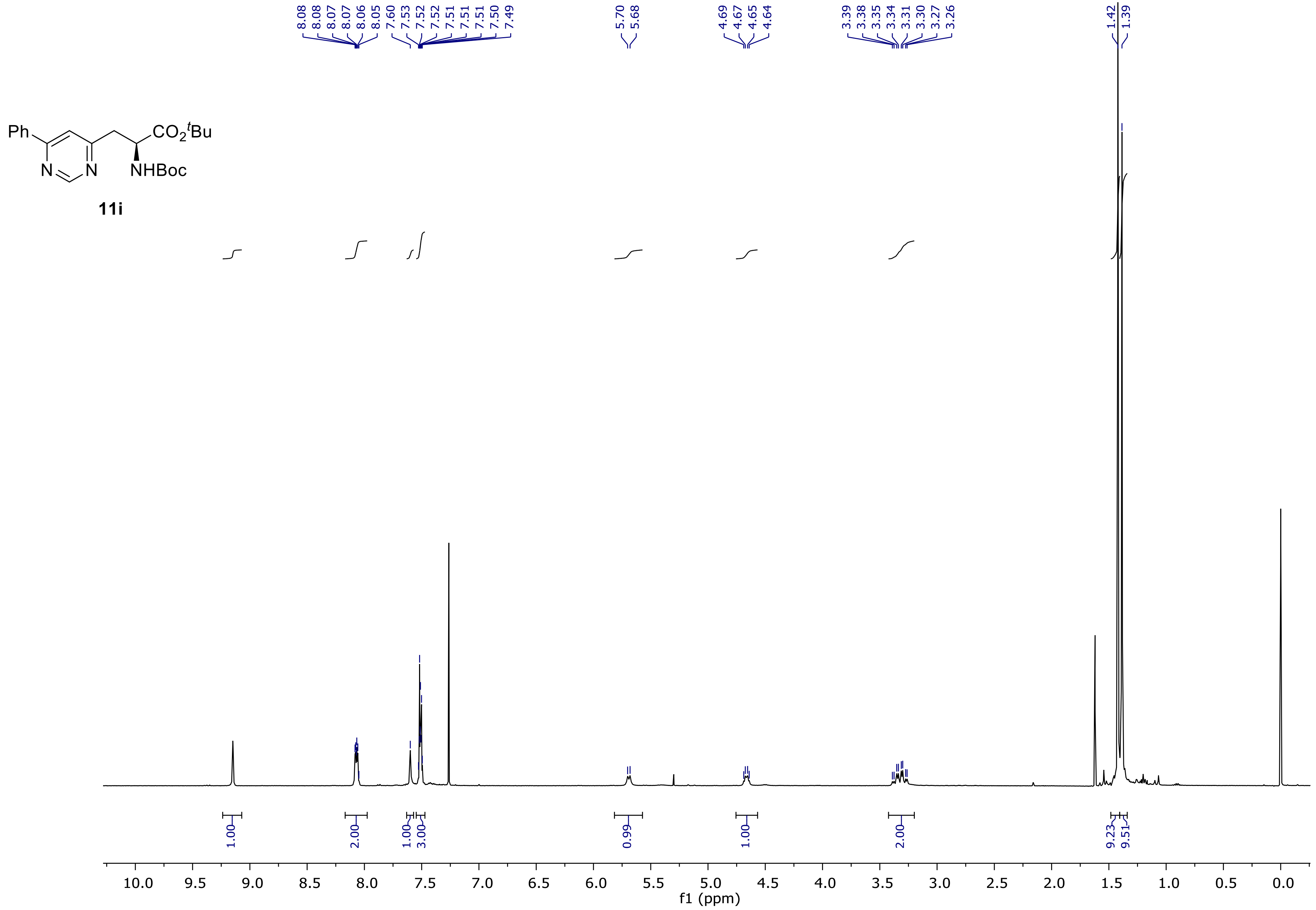

$^{13}C\{^{1}H\}$ NMR (101 MHz, $CDCl_3$)

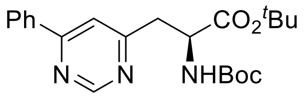

11i

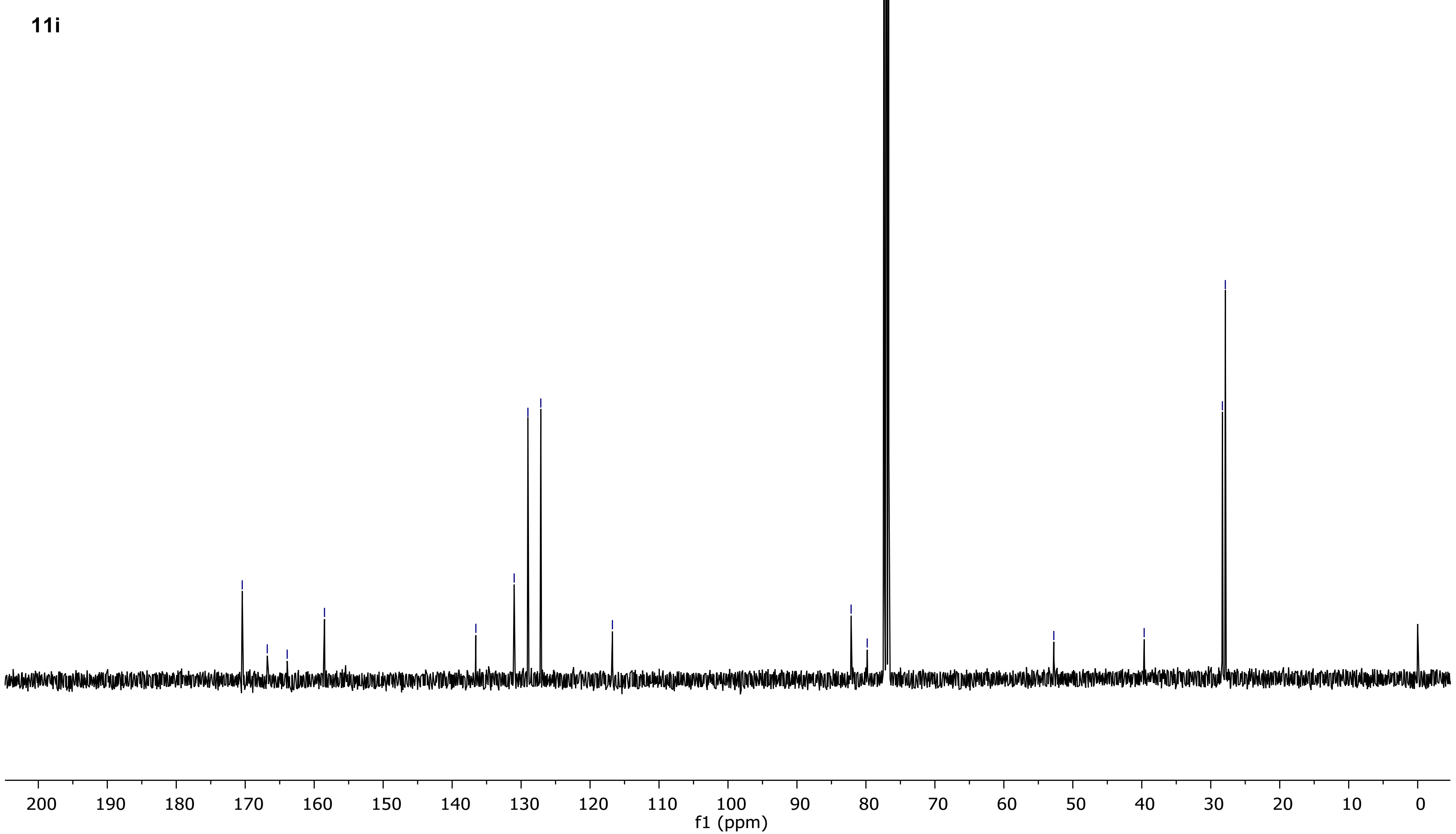

^{1}H NMR (400 MHz, CDCl$_3$)

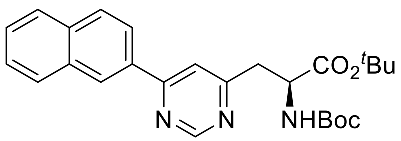

11j

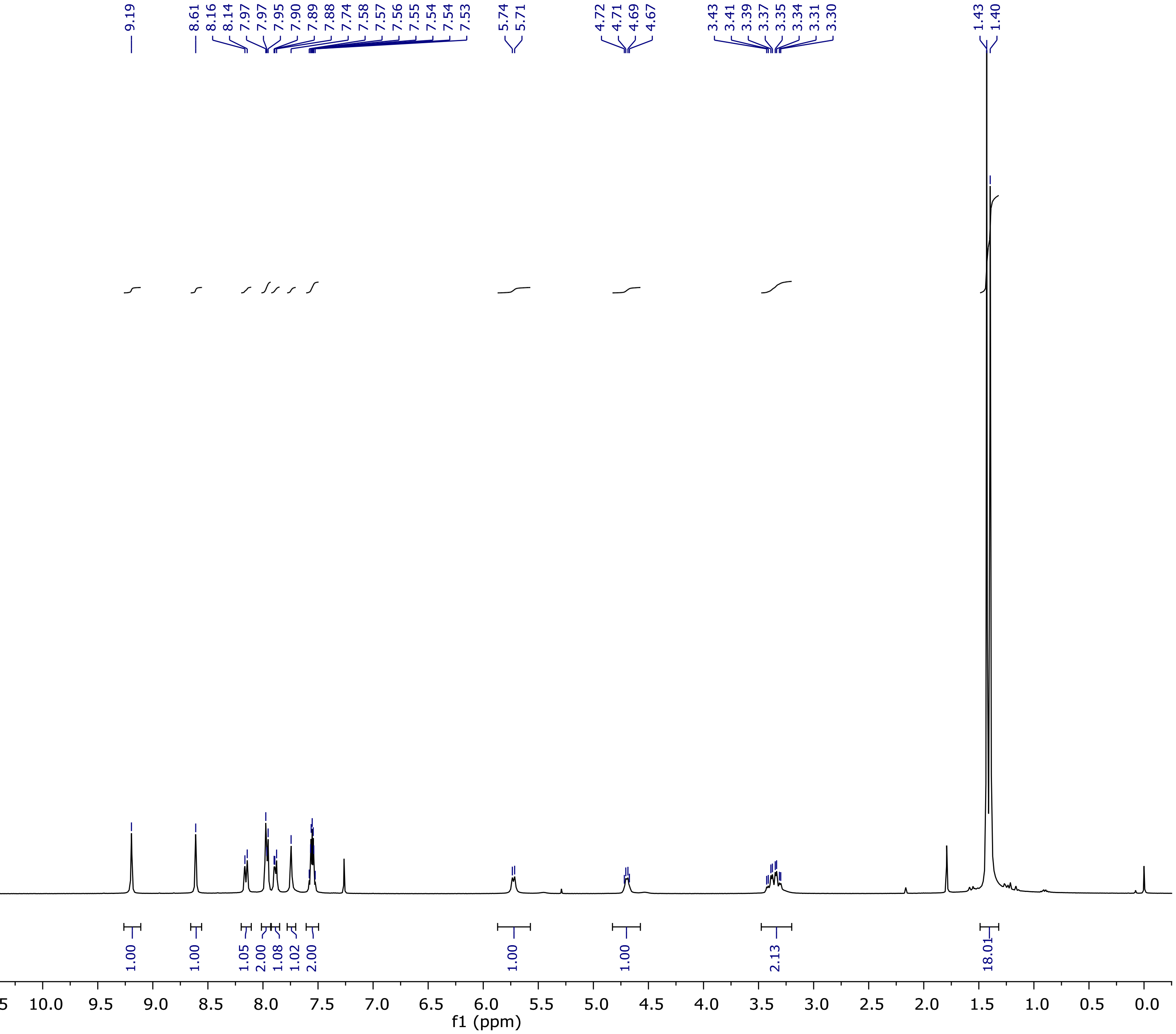

$^{13}C\{^1H\}$ NMR (101 MHz, $CDCl_3$)

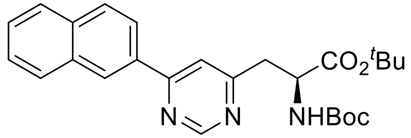

11j

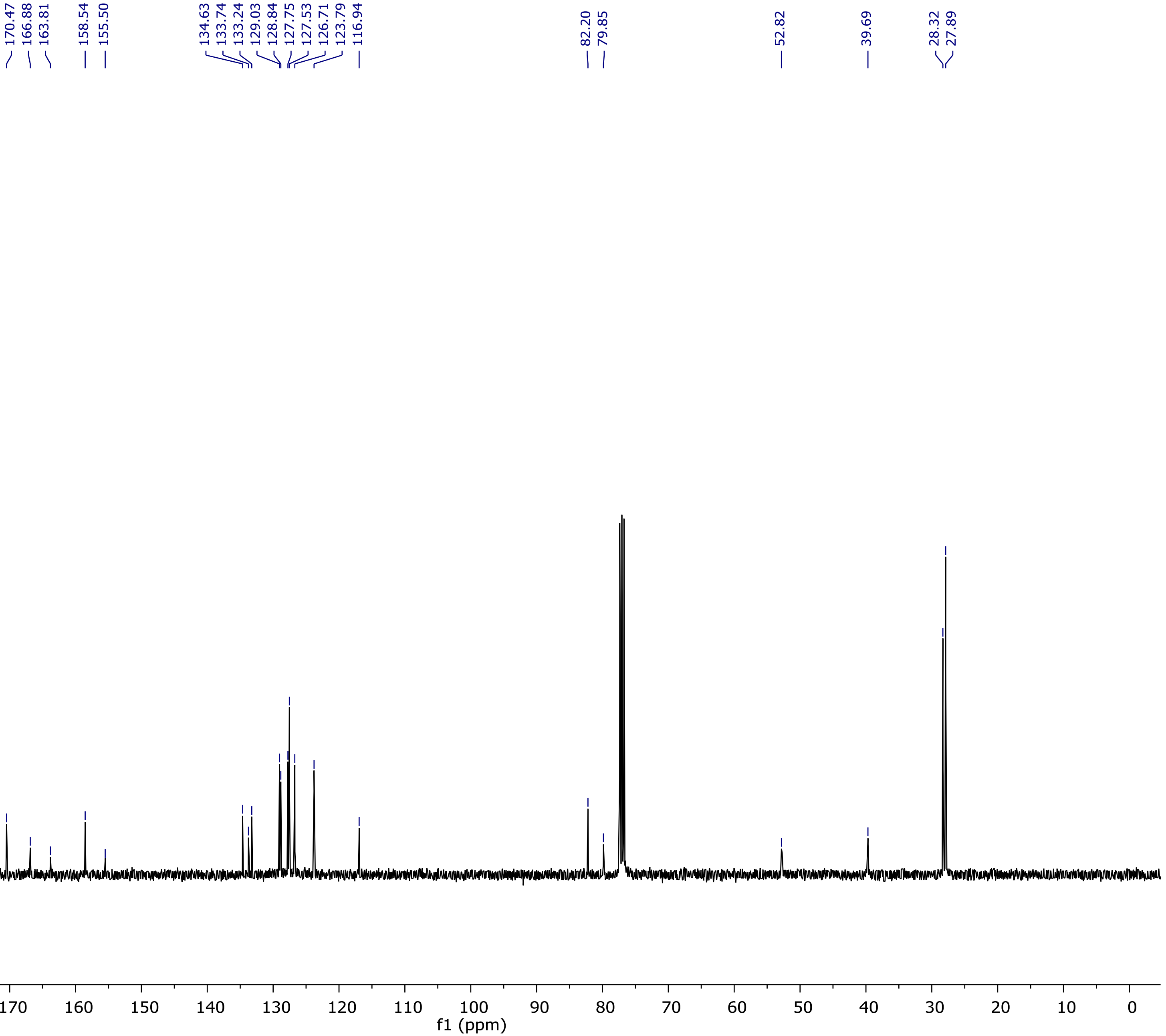

^{1}H NMR (400 MHz, $CDCl_3$)

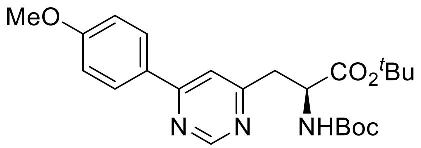

11k

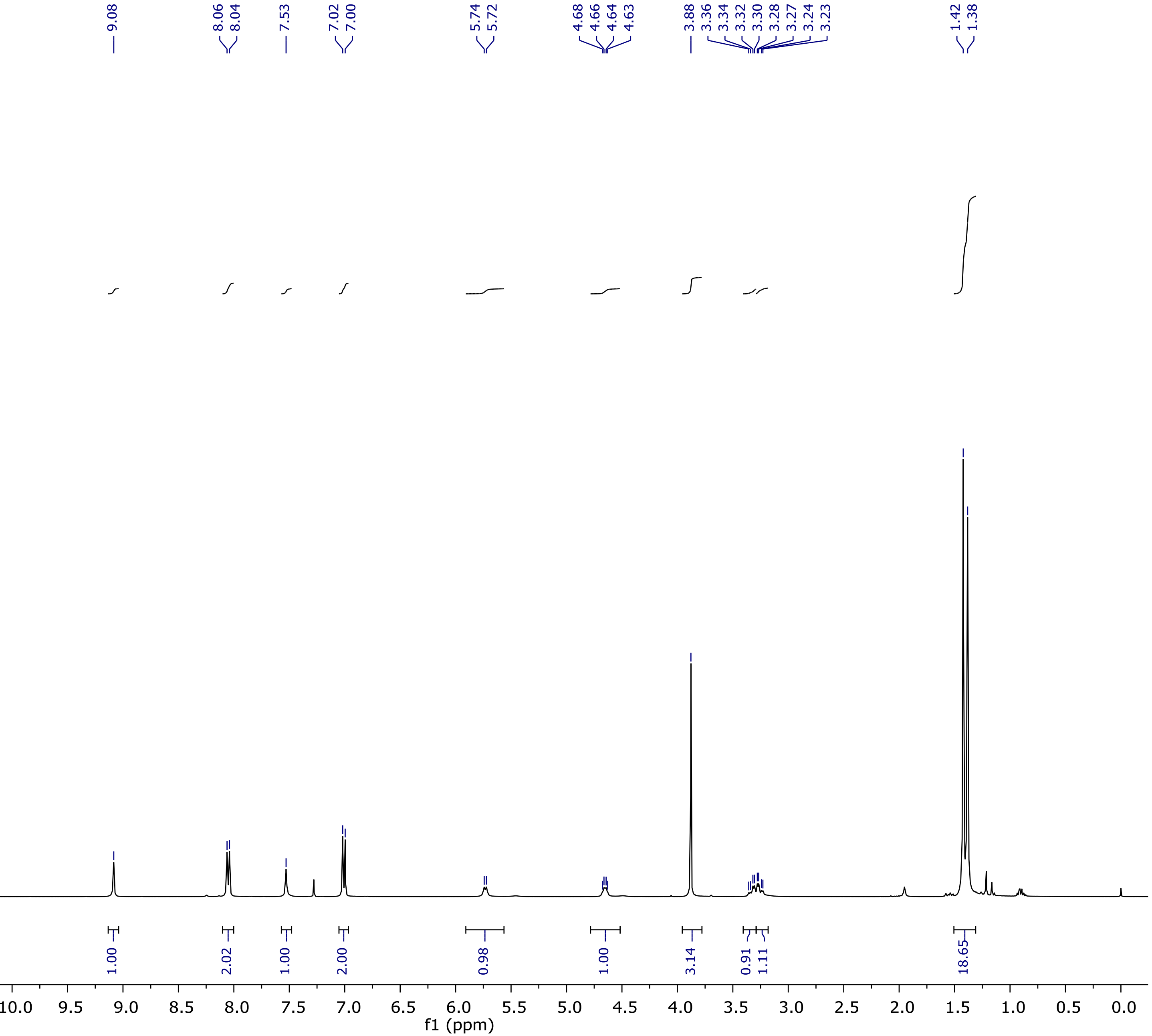

$^{13}C\{^1H\}$ NMR (101 MHz, CDCl$_3$)

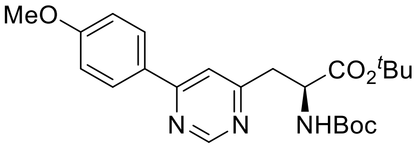

11k

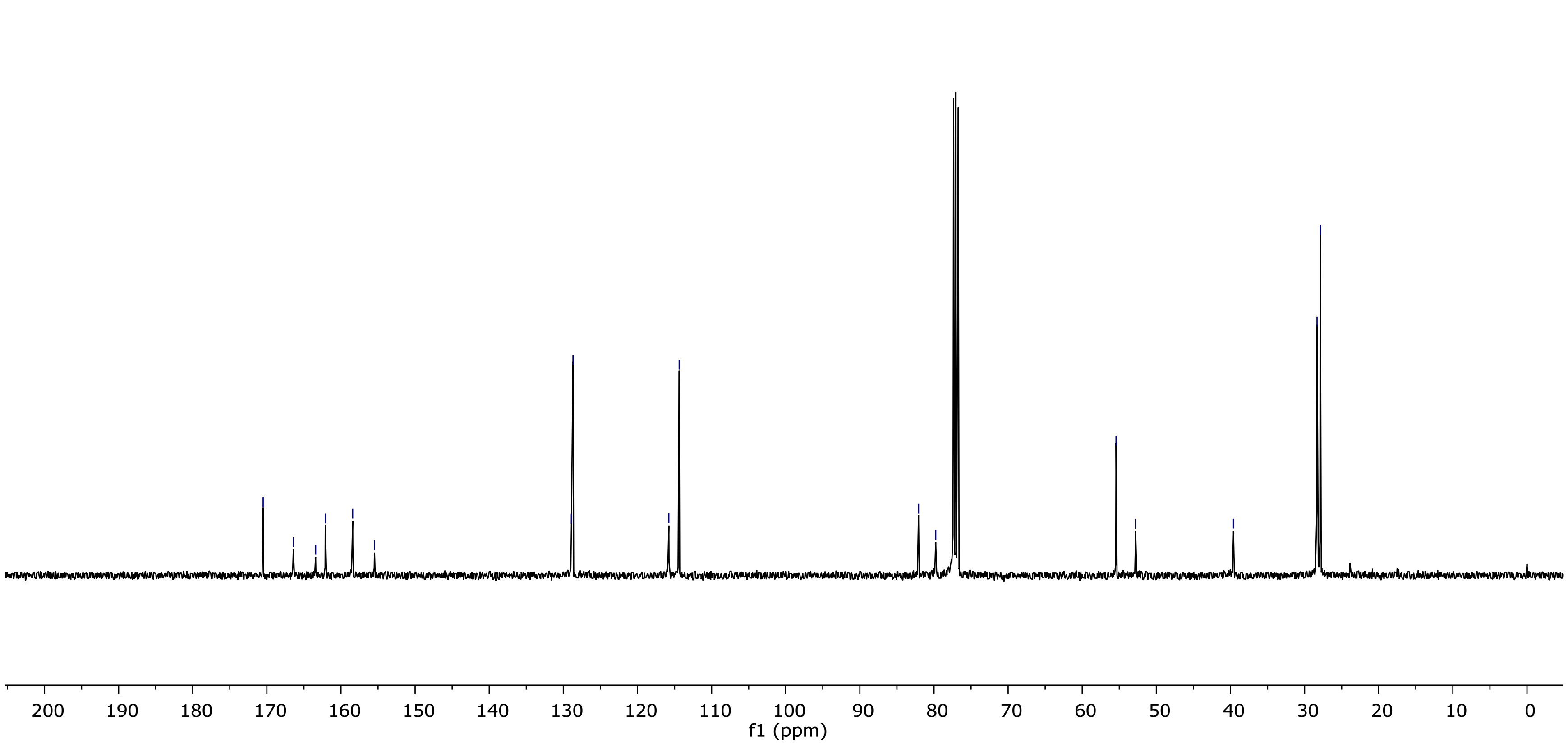

^{1}H NMR (400 MHz, $CDCl_3$)

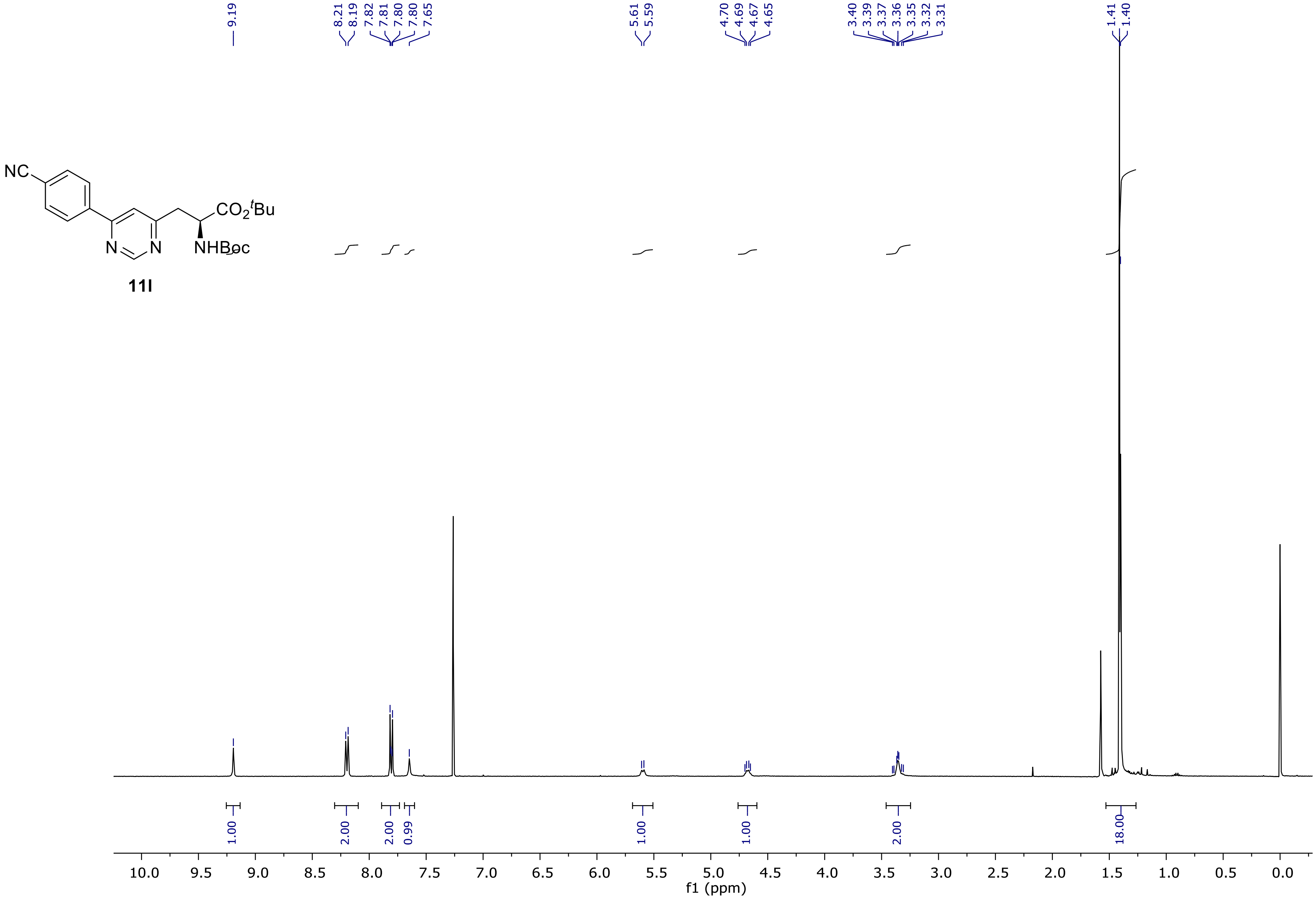

11l

$^{13}C\{^1H\}$ NMR (101 MHz, $CDCl_3$)

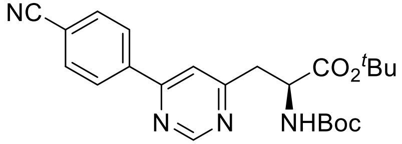

11l

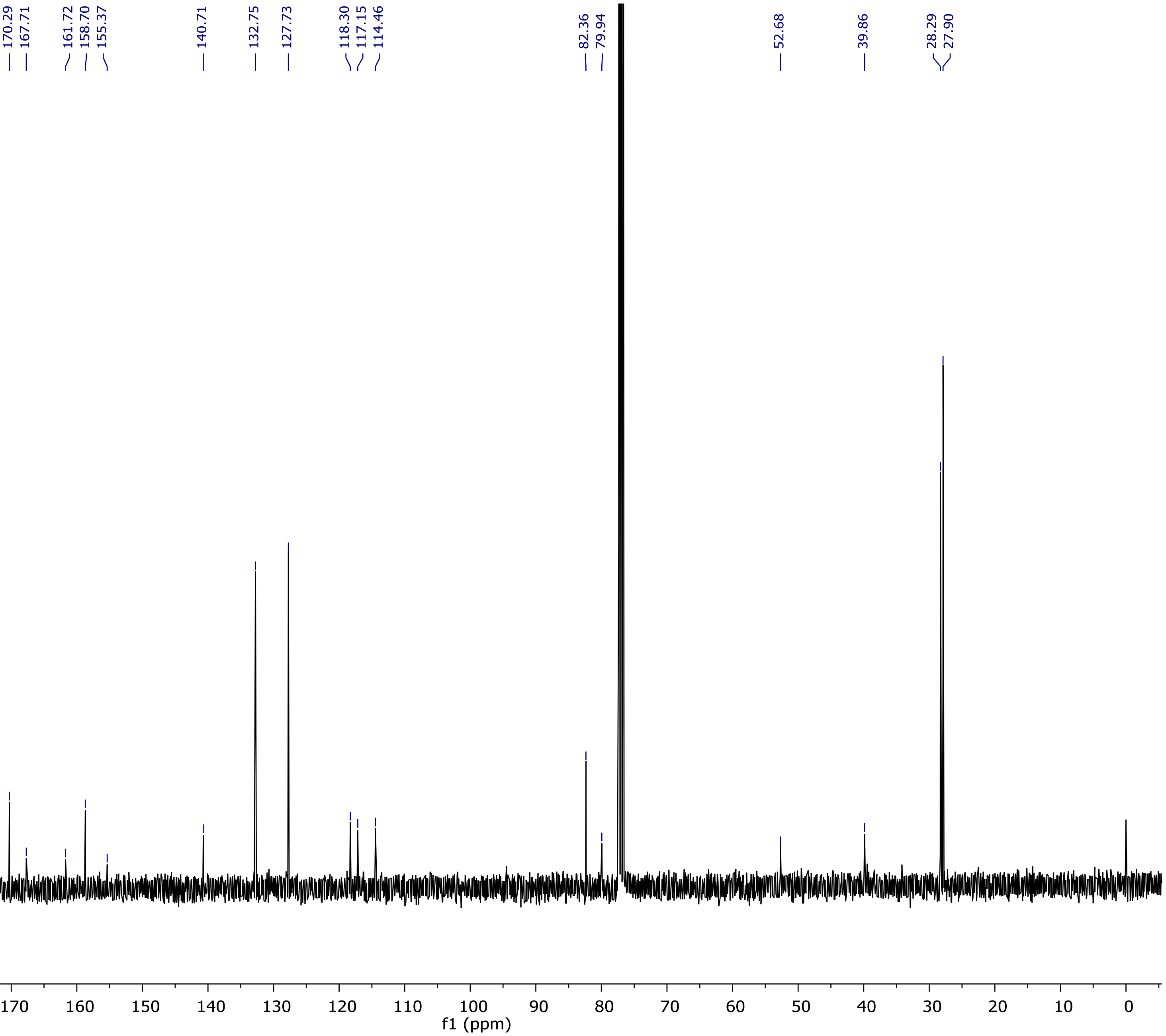

^{1}H NMR (400 MHz, CD_3OD)

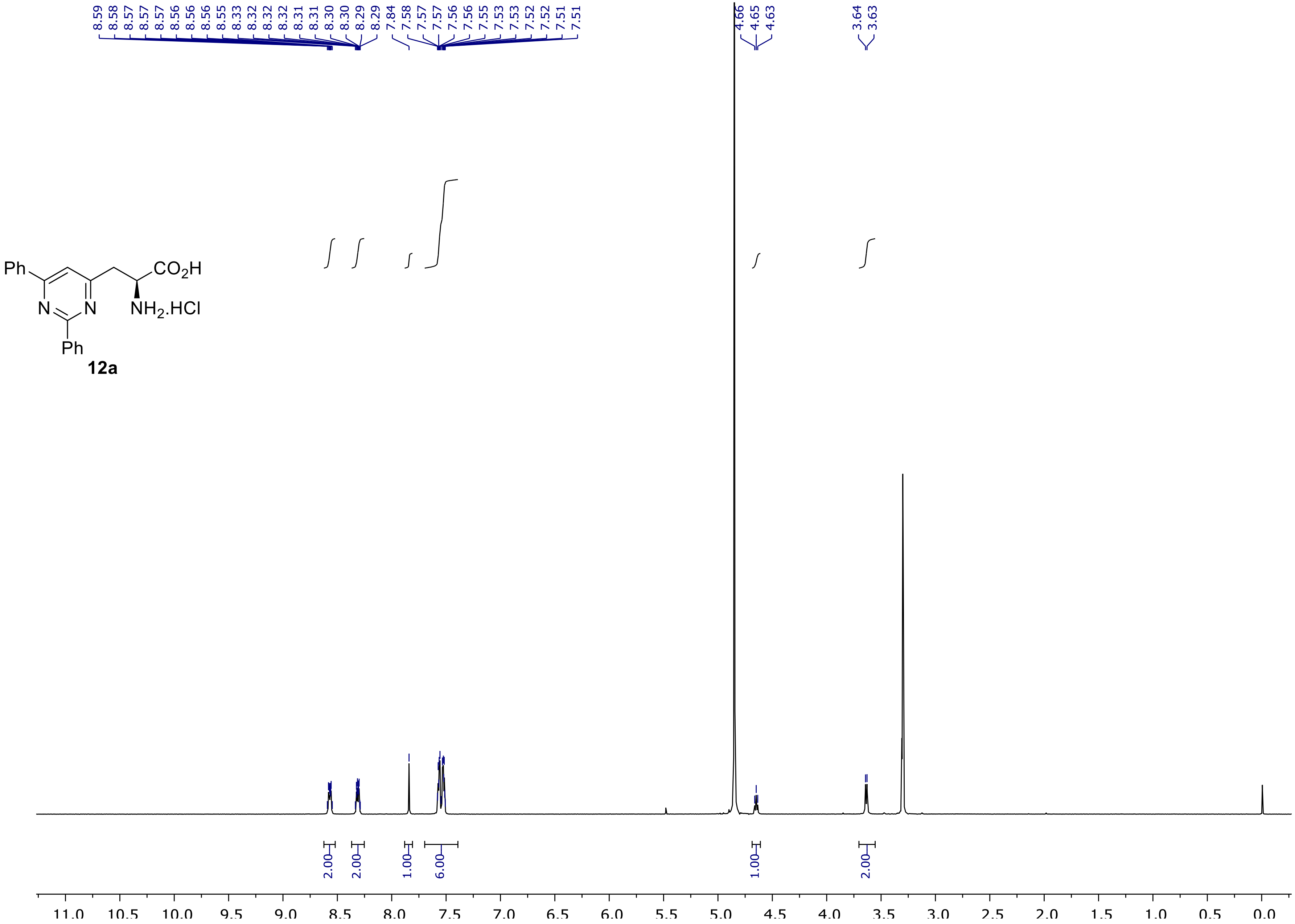

$^{13}C\{^{1}H\}$ NMR (101 MHz, CD_3OD)

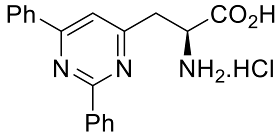

12a

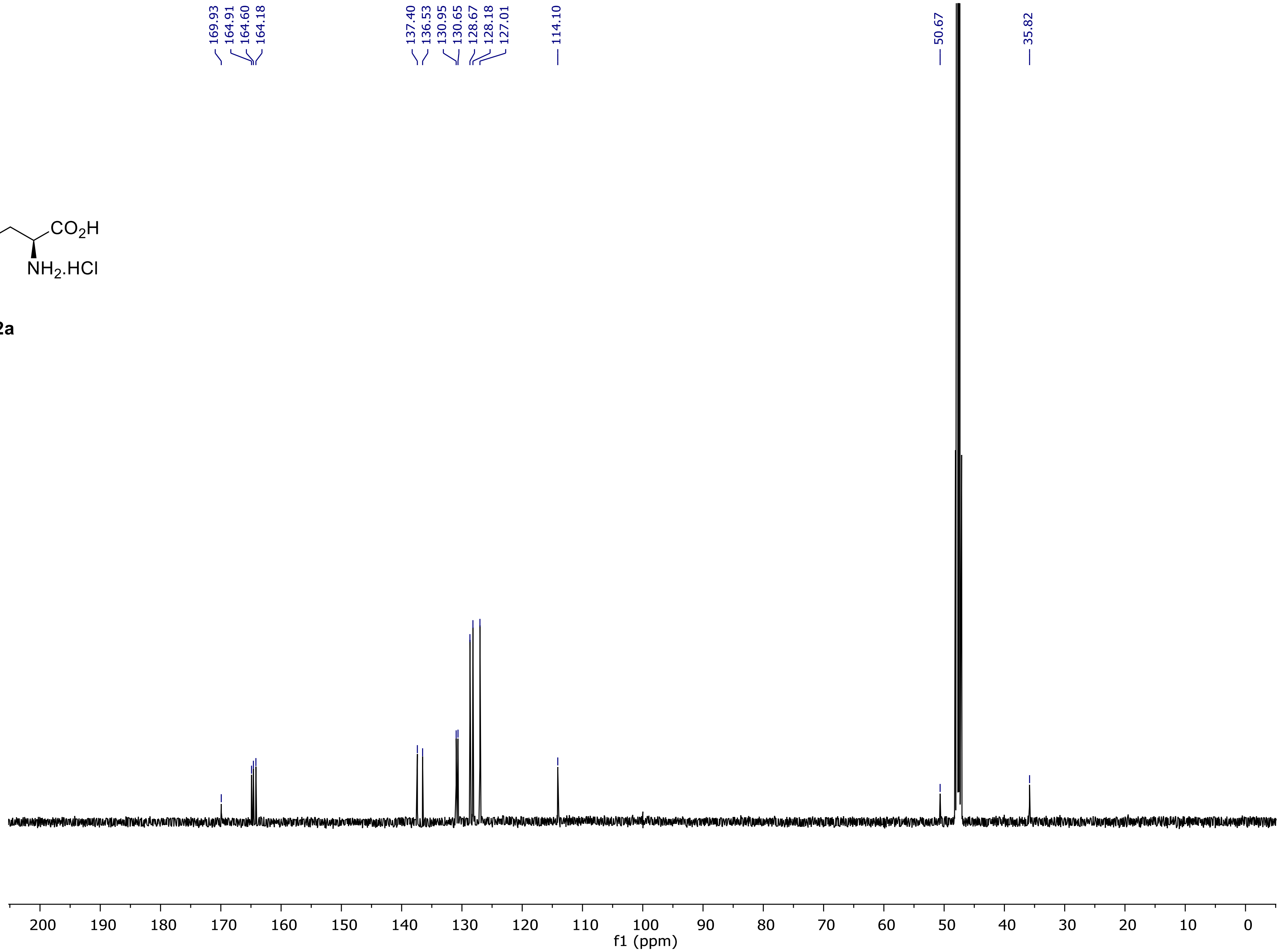

^{1}H NMR (400 MHz, CD_3OD)

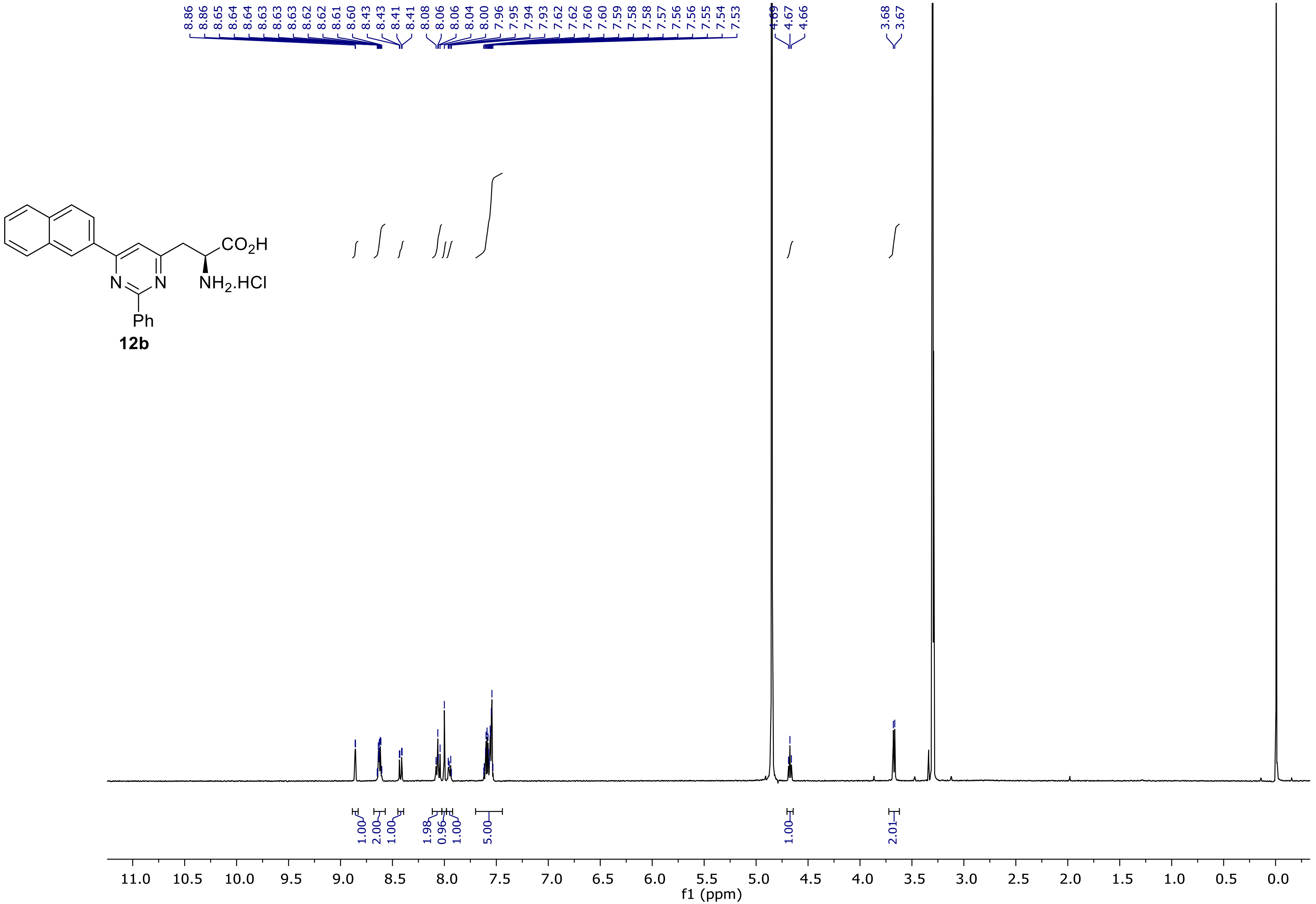

$^{13}C\{^{1}H\}$ NMR (101 MHz, CD_3OD)

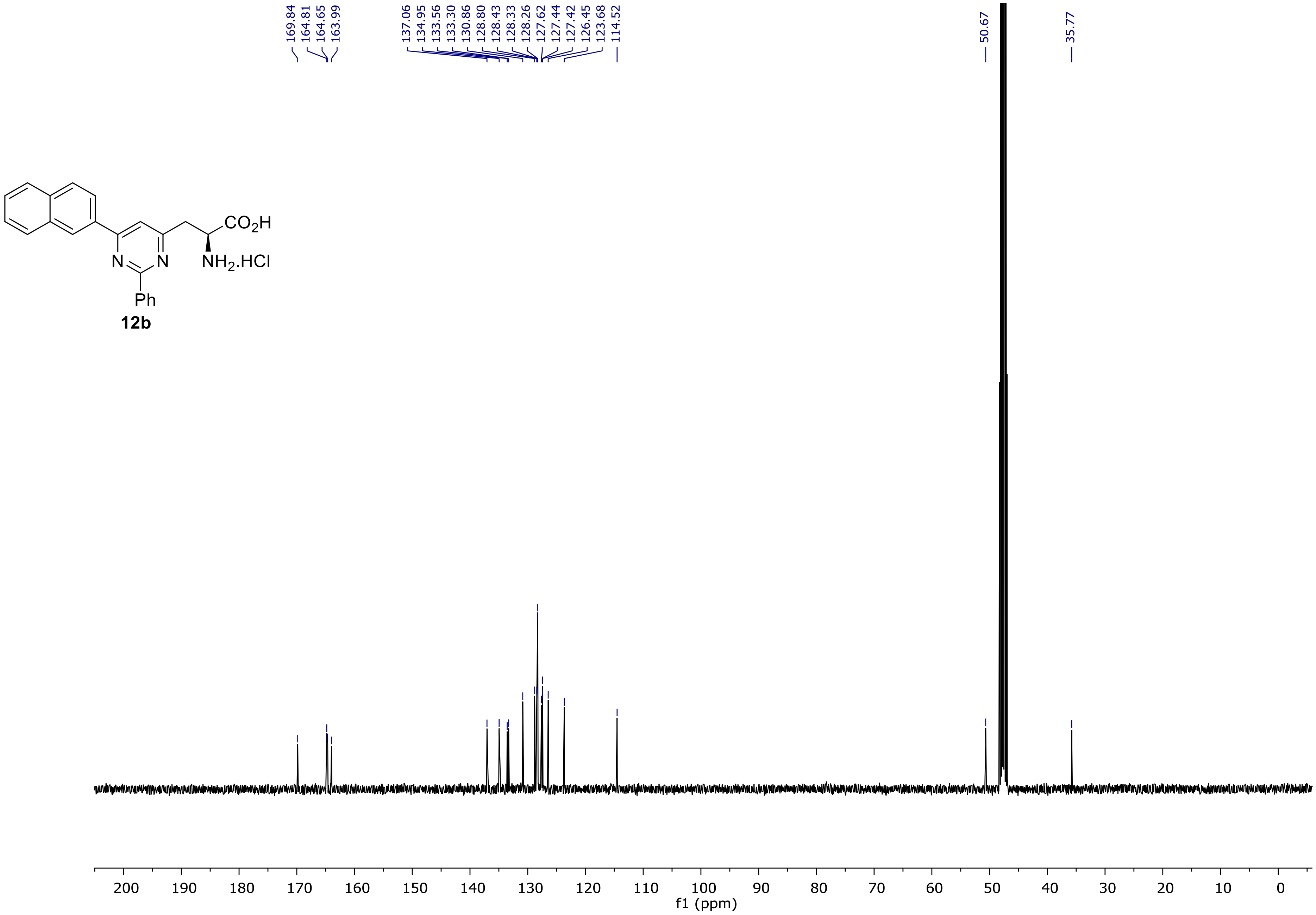

12b

^{1}H NMR (400 MHz, CD_3OD)

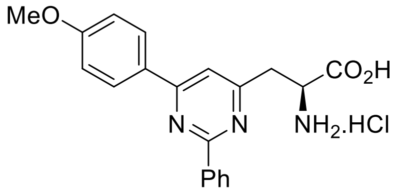

12c

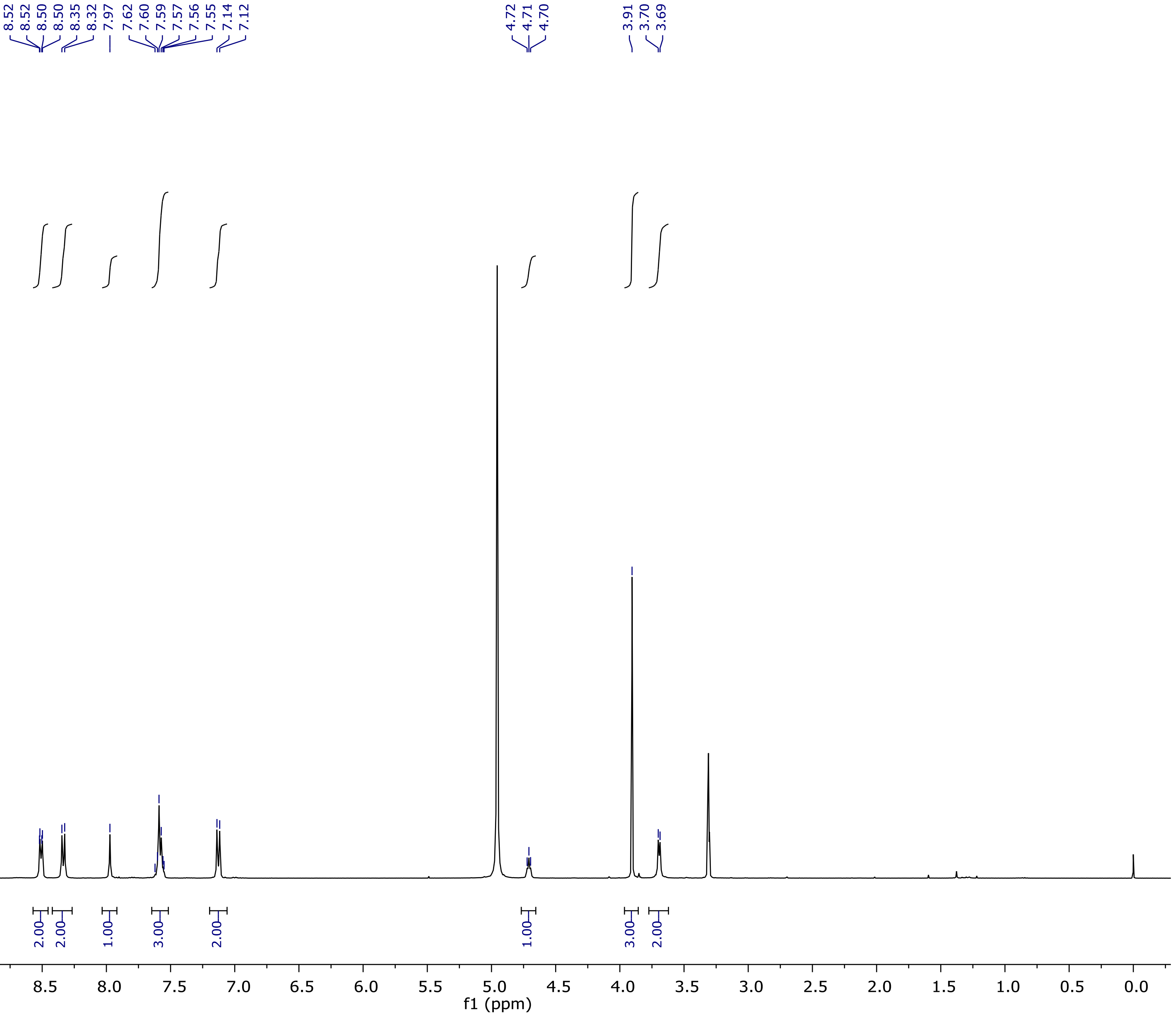

$^{13}C\{^{1}H\}$ NMR (101 MHz, CD_3OD)

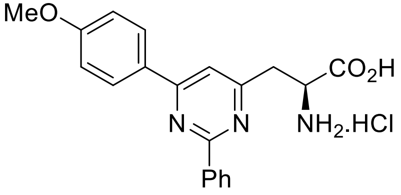

12c

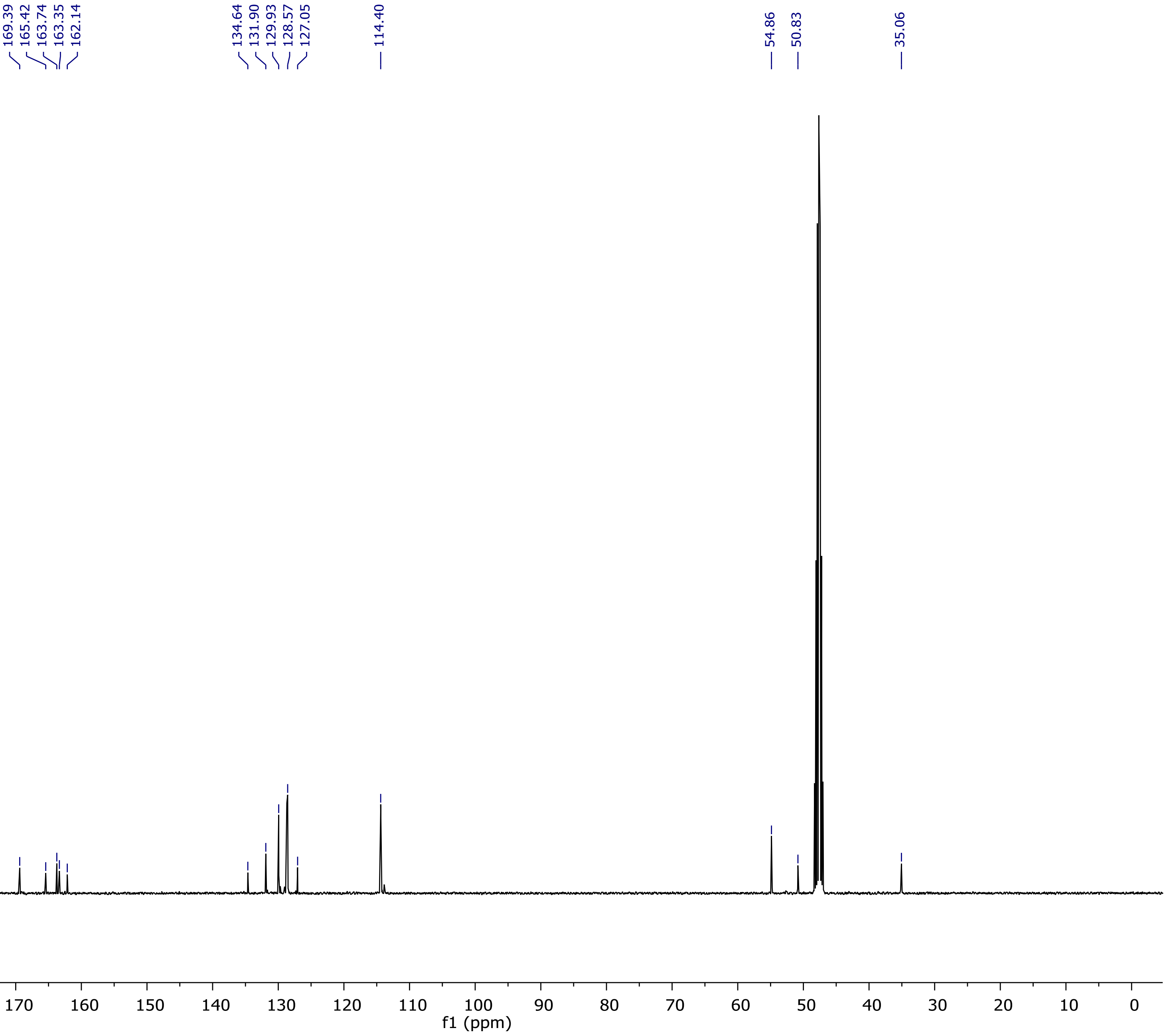

^{1}H NMR (400 MHz, CD_3OD)

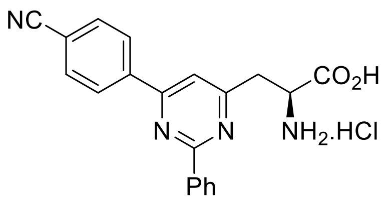

12d

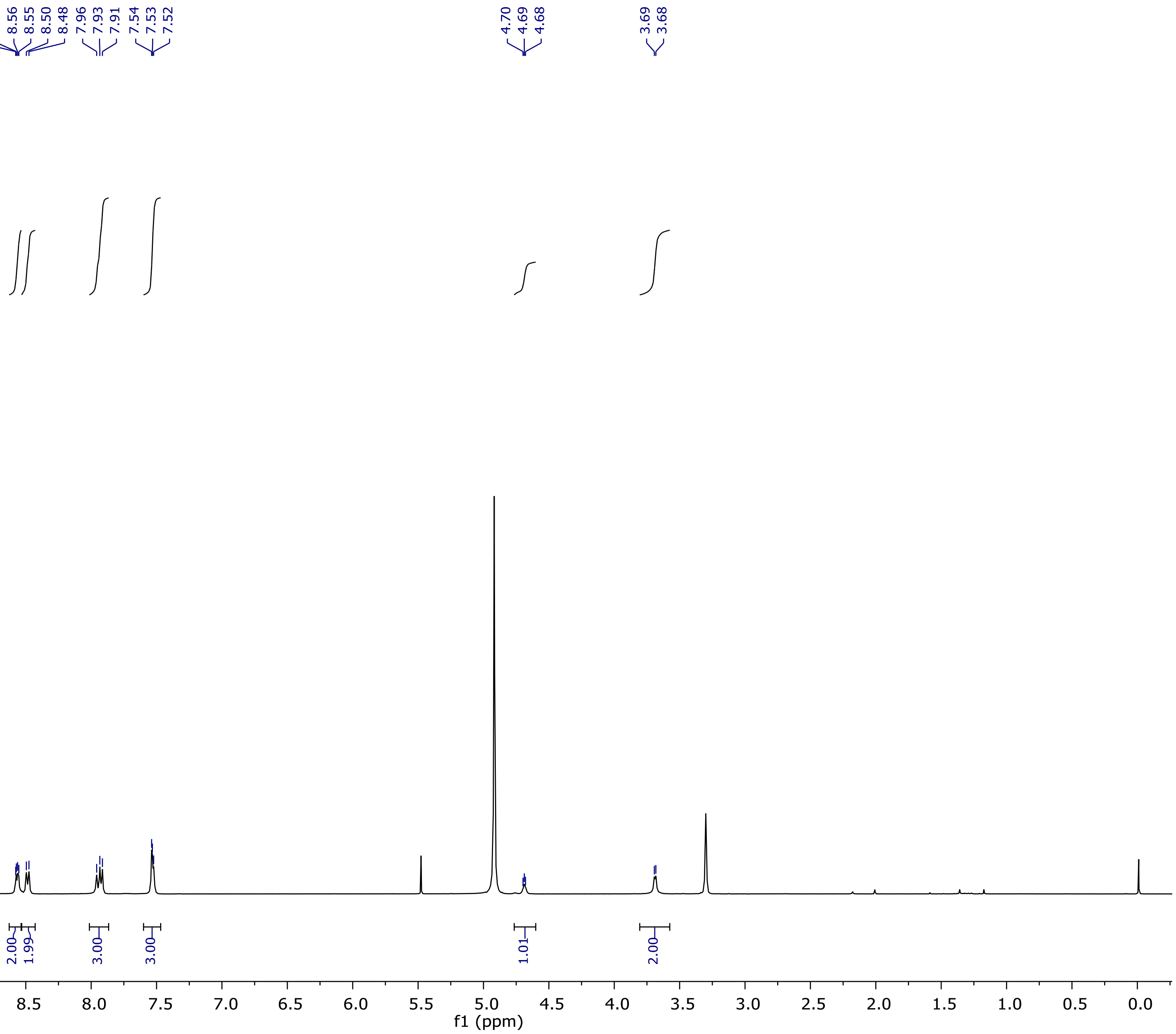

$^{13}C\{^{1}H\}$ NMR (101 MHz, CD_3OD)

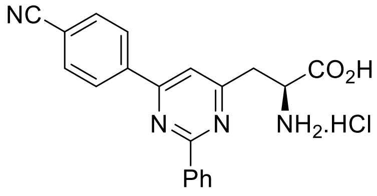

12d

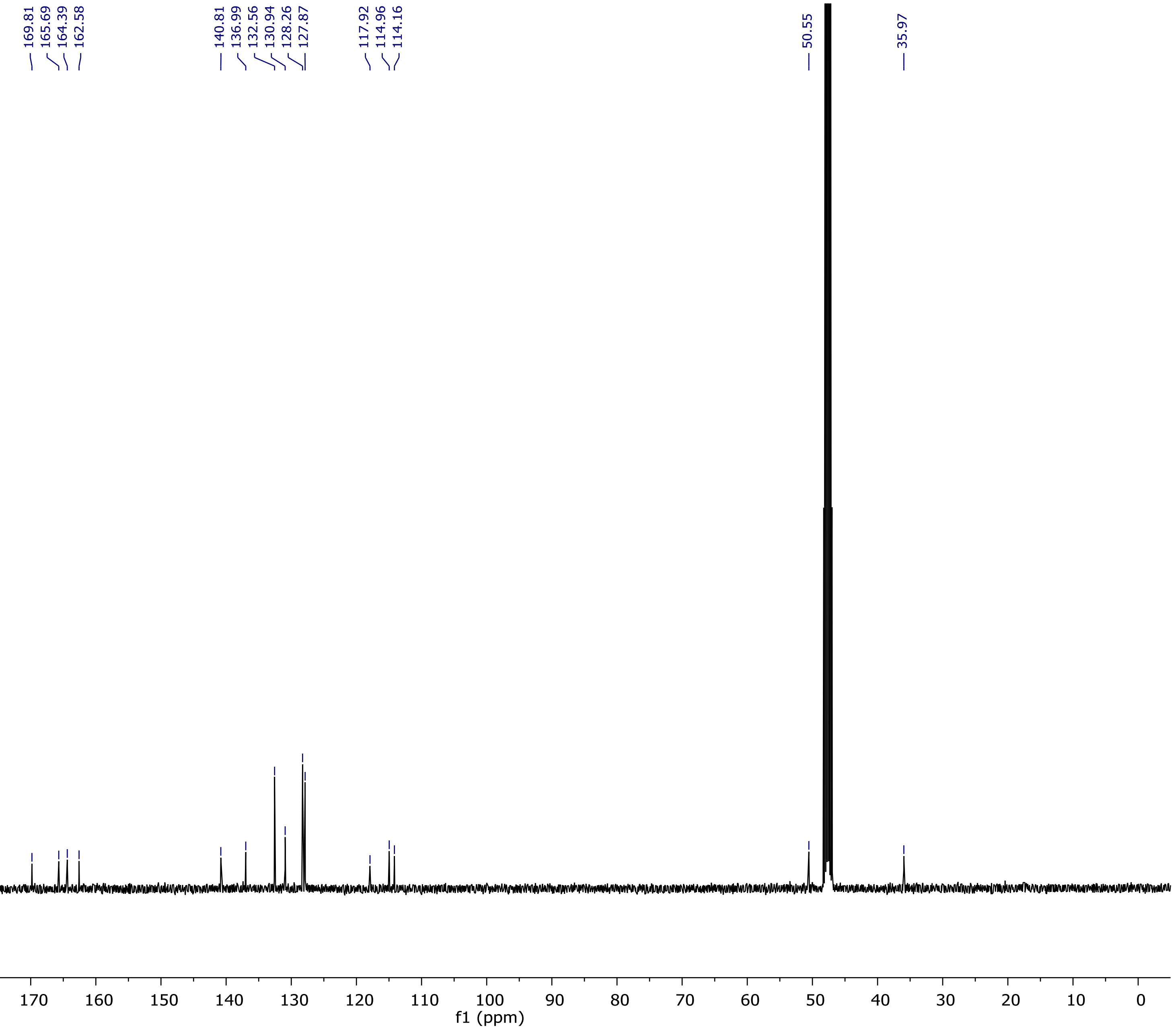

^{1}H NMR (400 MHz, CD_3OD)

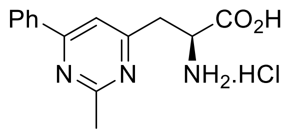

12e

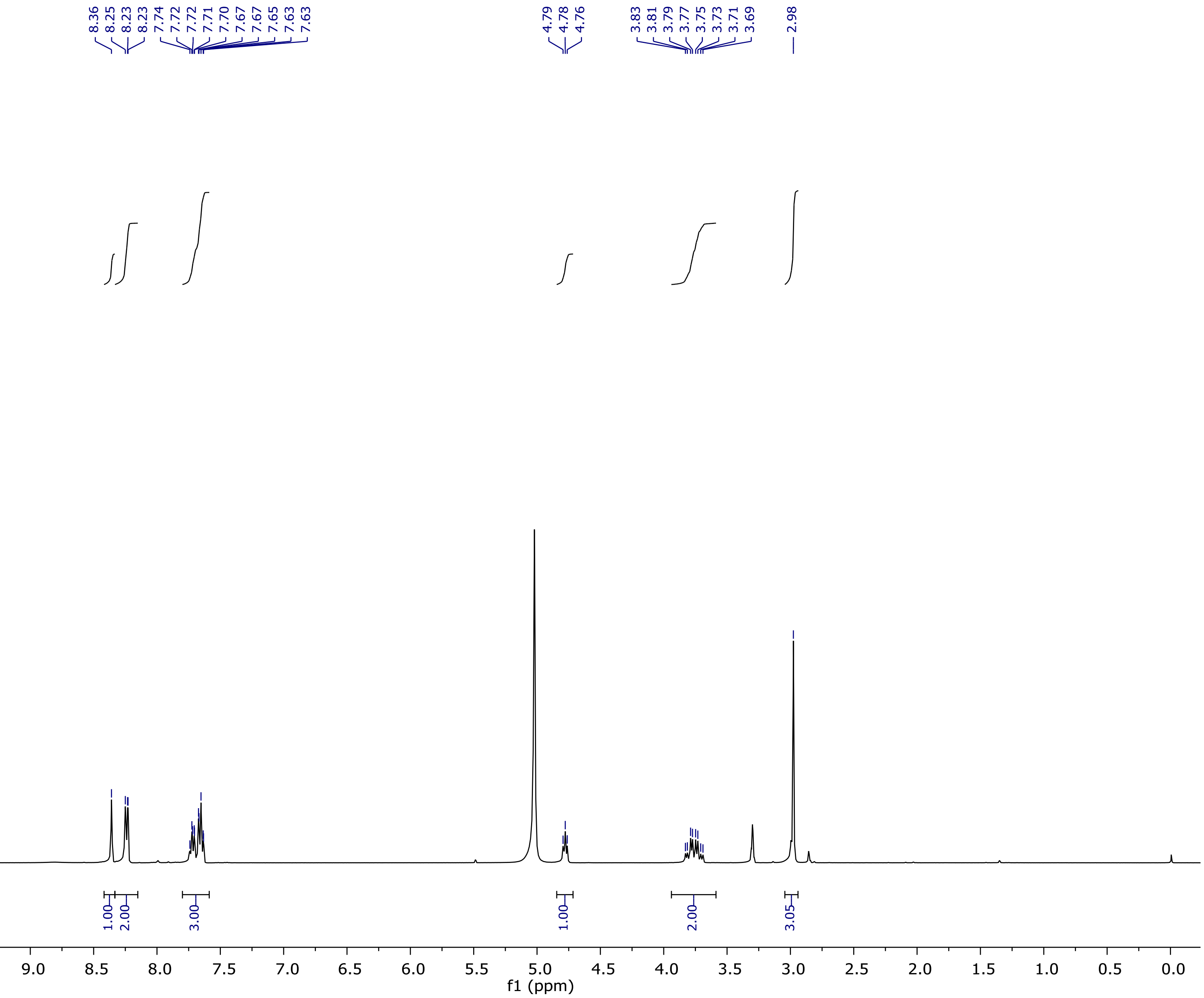

$^{13}C\{^{1}H\}$ NMR (101 MHz, CD_3OD)

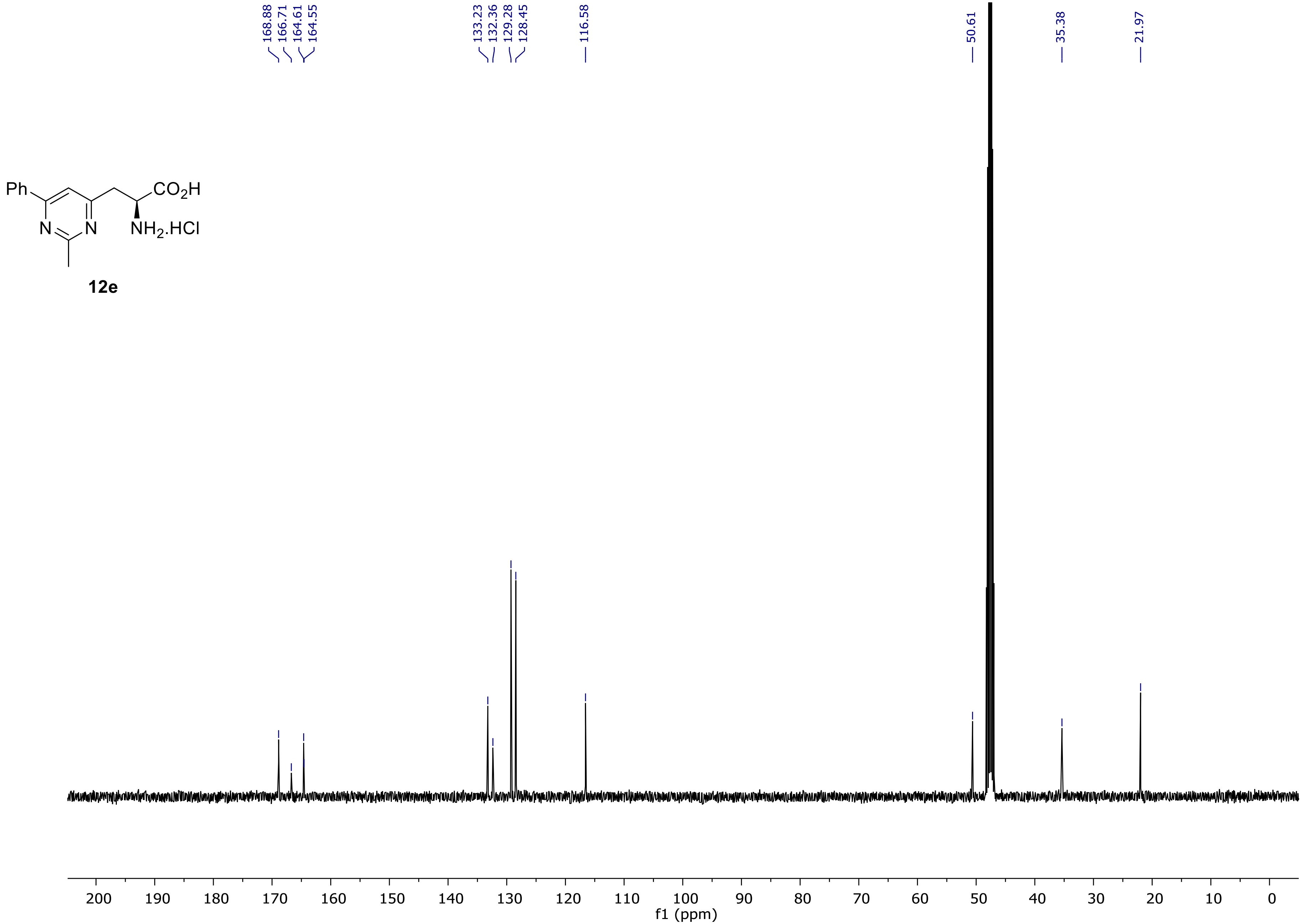

^{1}H NMR (400 MHz, CD_3OD)

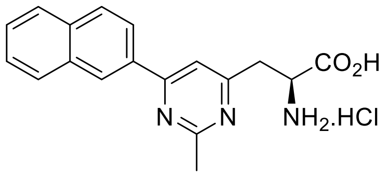

12f

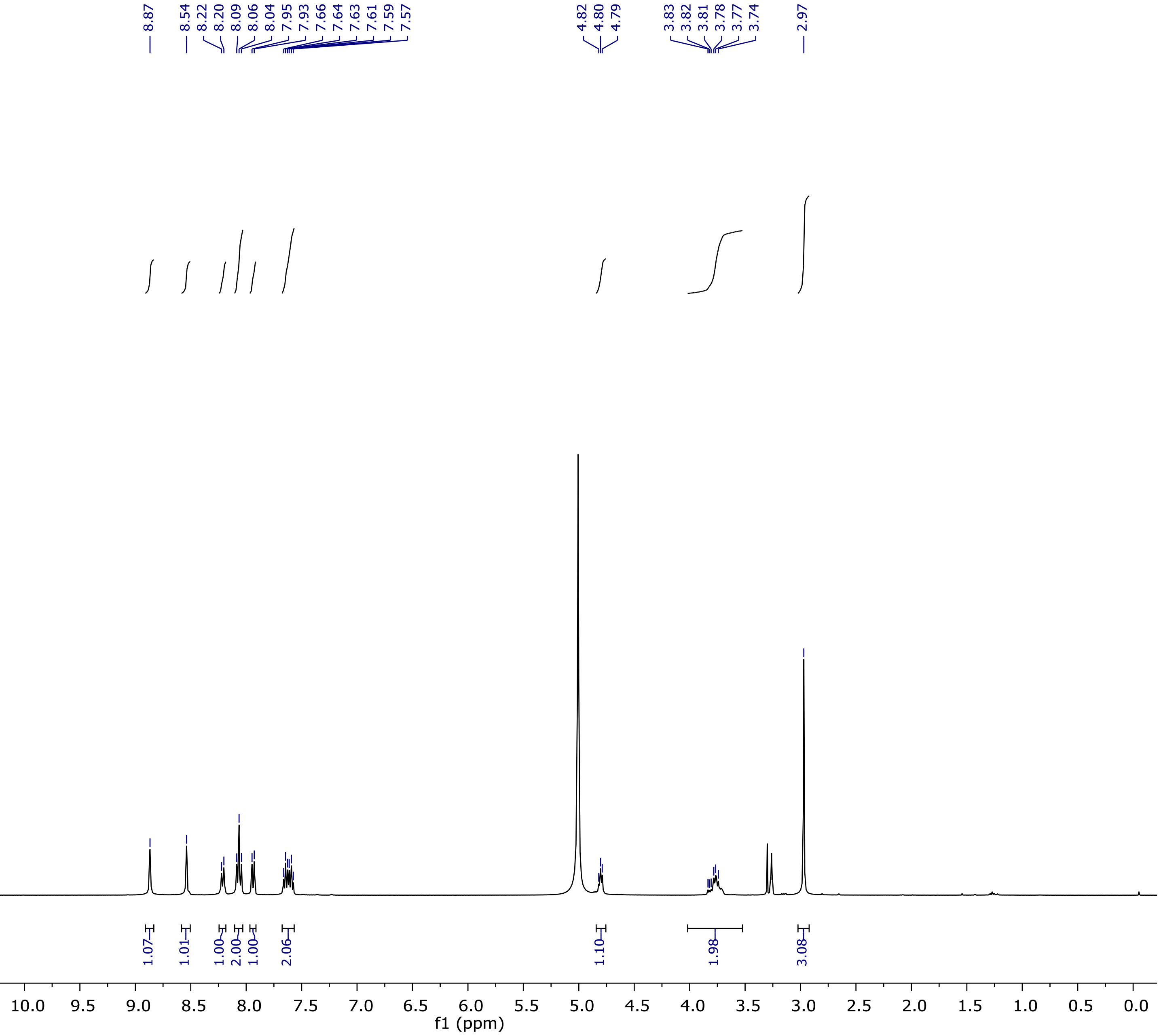

$^{13}C\{^{1}H\}$ NMR (101 MHz, CD_3OD)

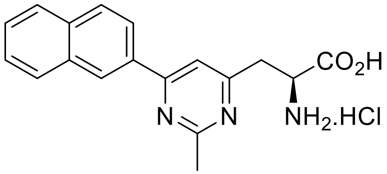

12f

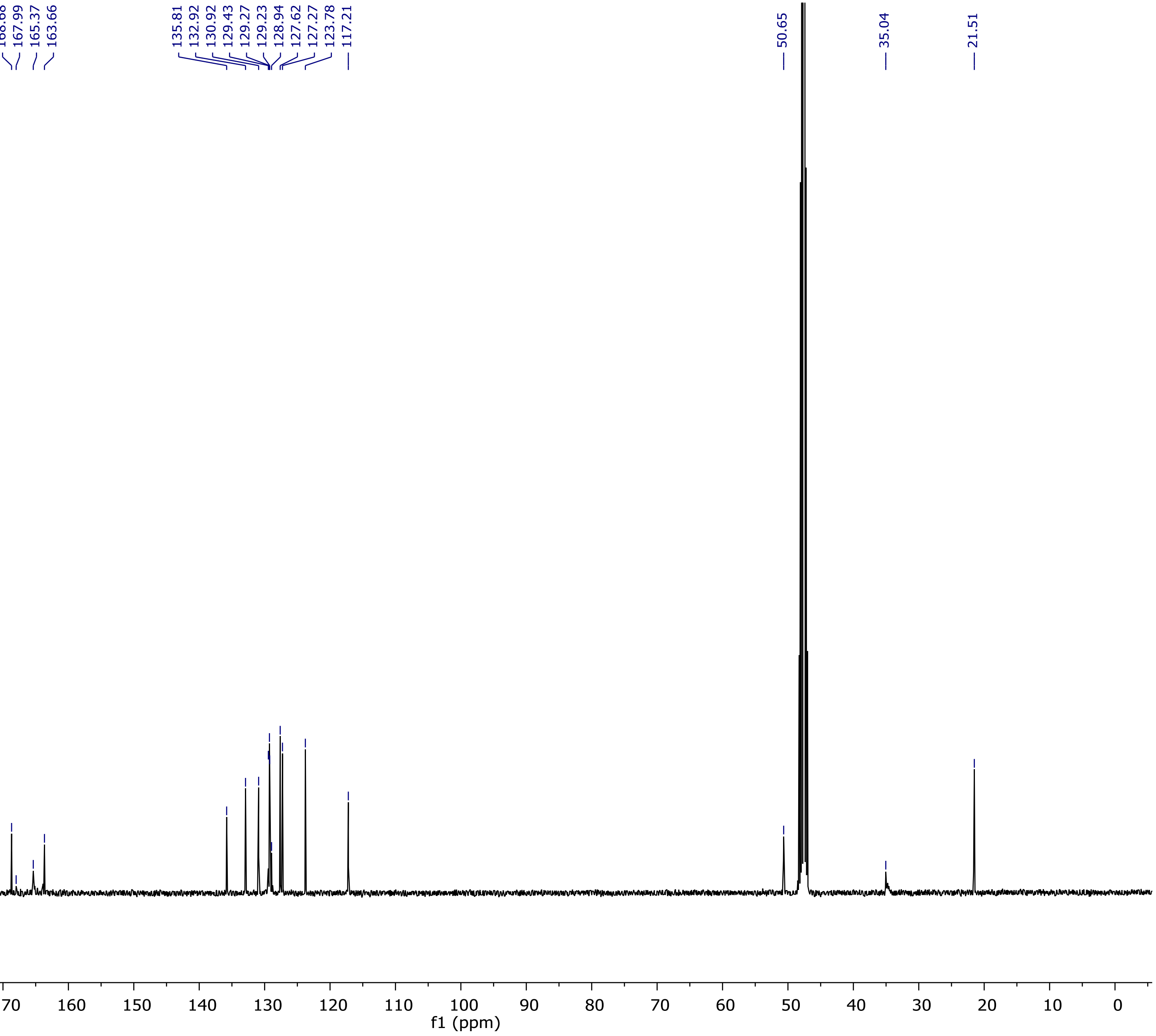

^{1}H NMR (400 MHz, CD_3OD)

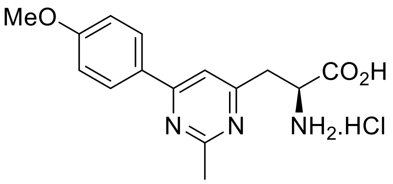

12g

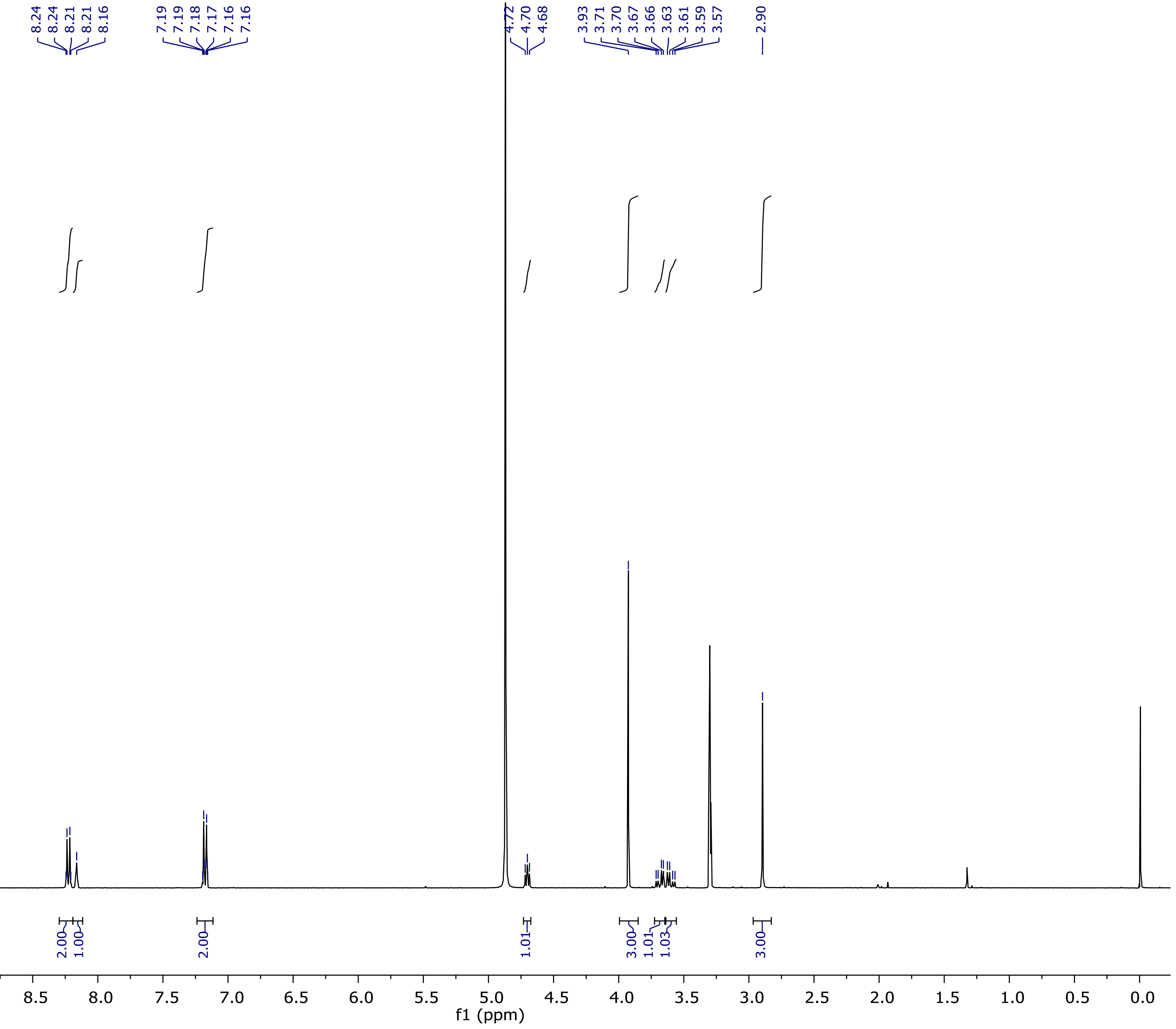

$^{13}C\{^1H\}$ NMR (101 MHz, CD_3OD)

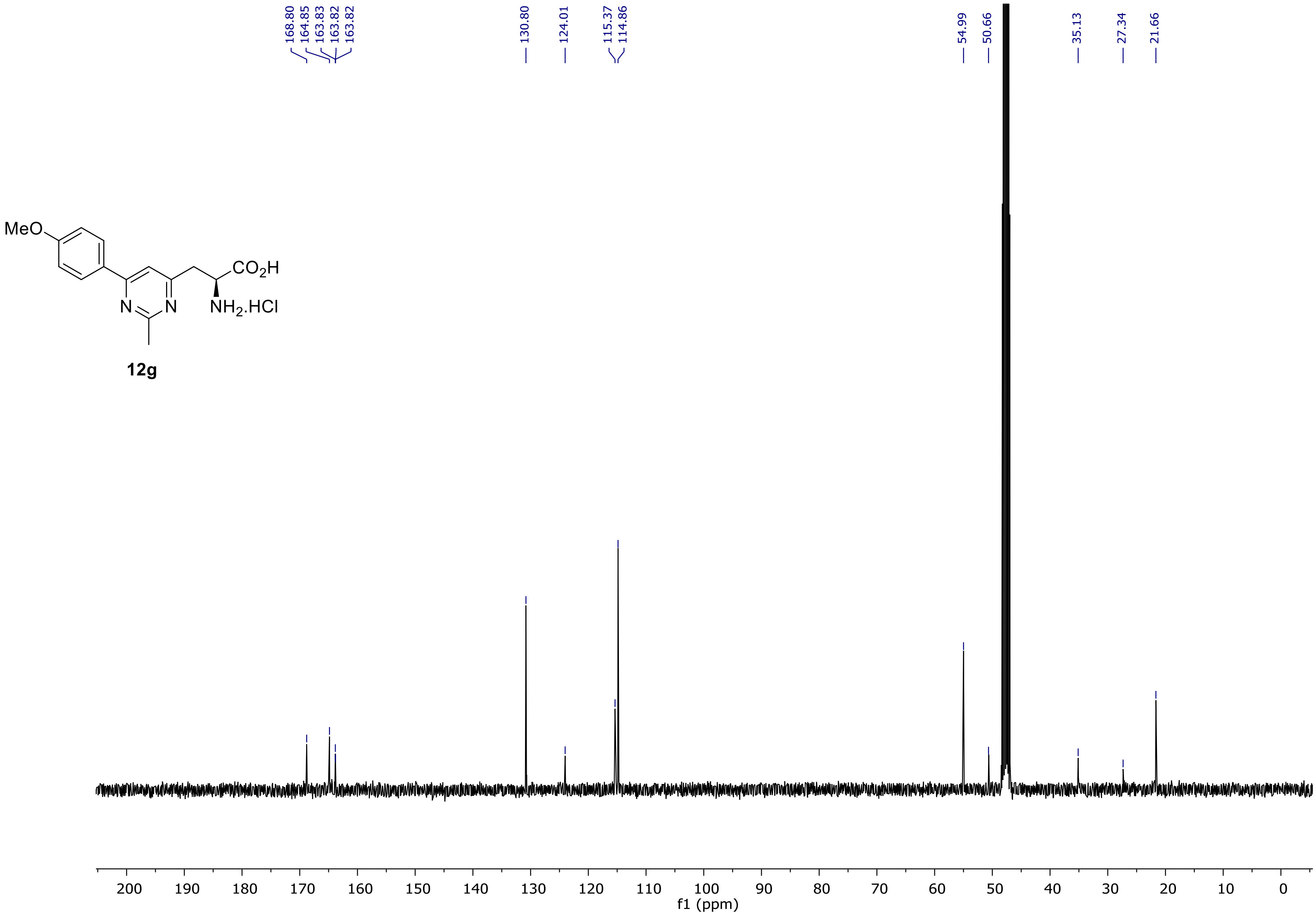

^{1}H NMR (400 MHz, CD_3OD)

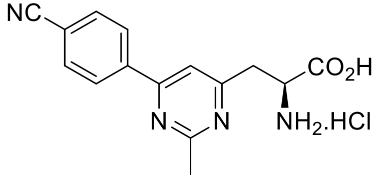

12h

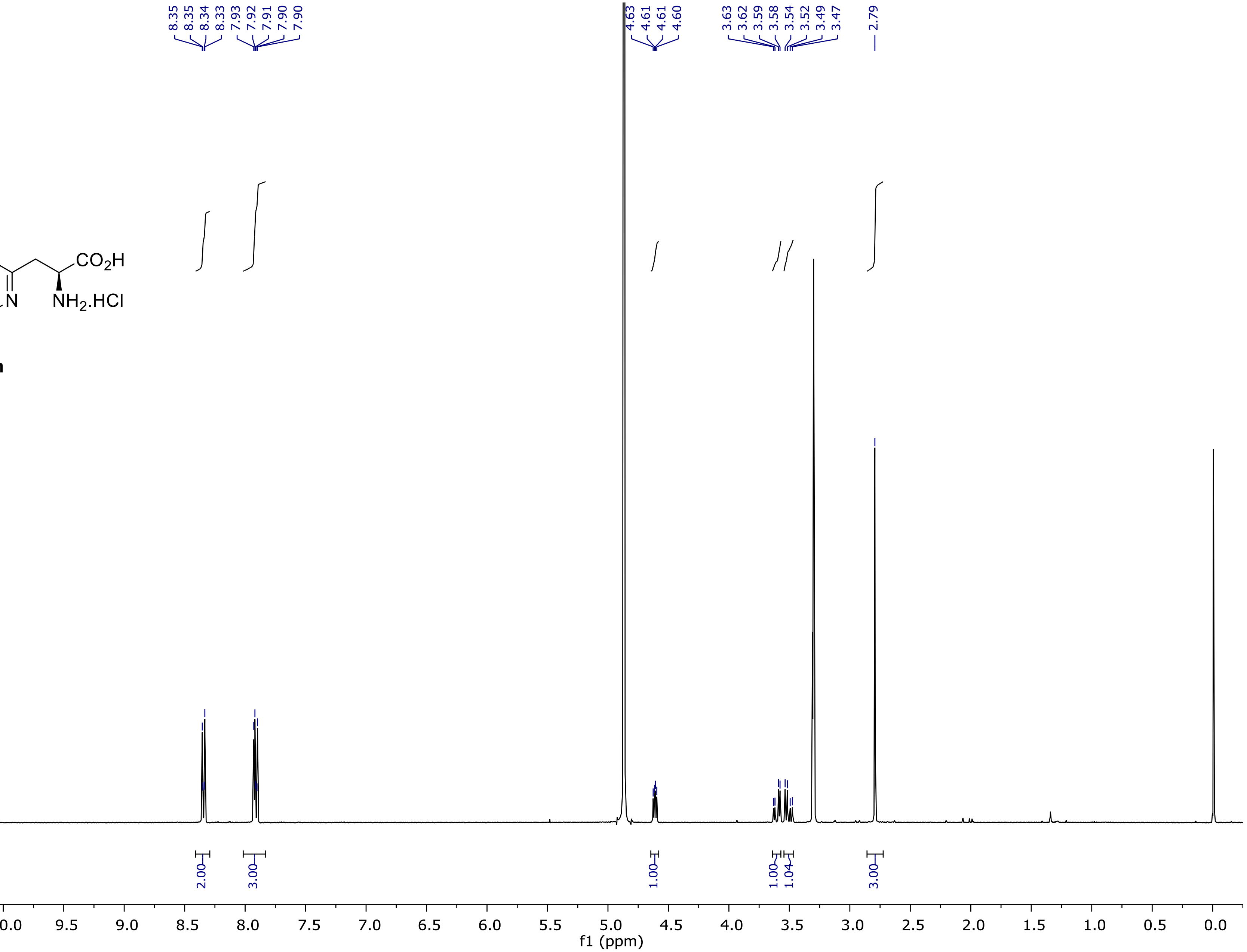

$^{13}C\{^{1}H\}$ NMR (101 MHz, CD_3OD)

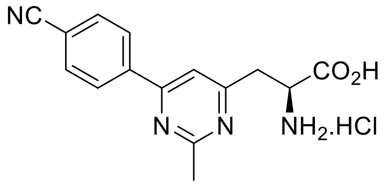

12h

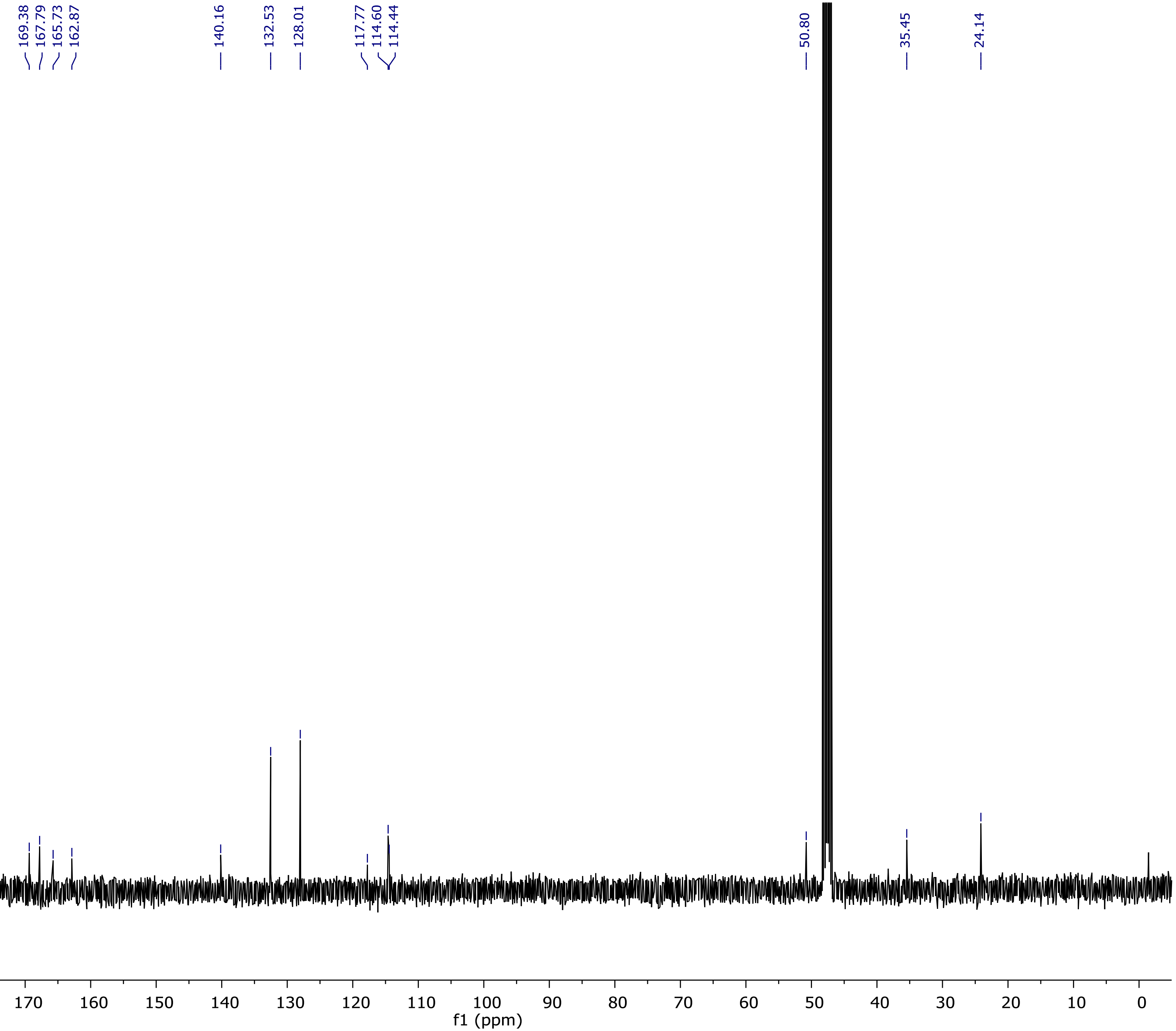

^{1}H NMR (400 MHz, CD_3OD)

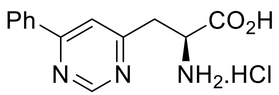

12i

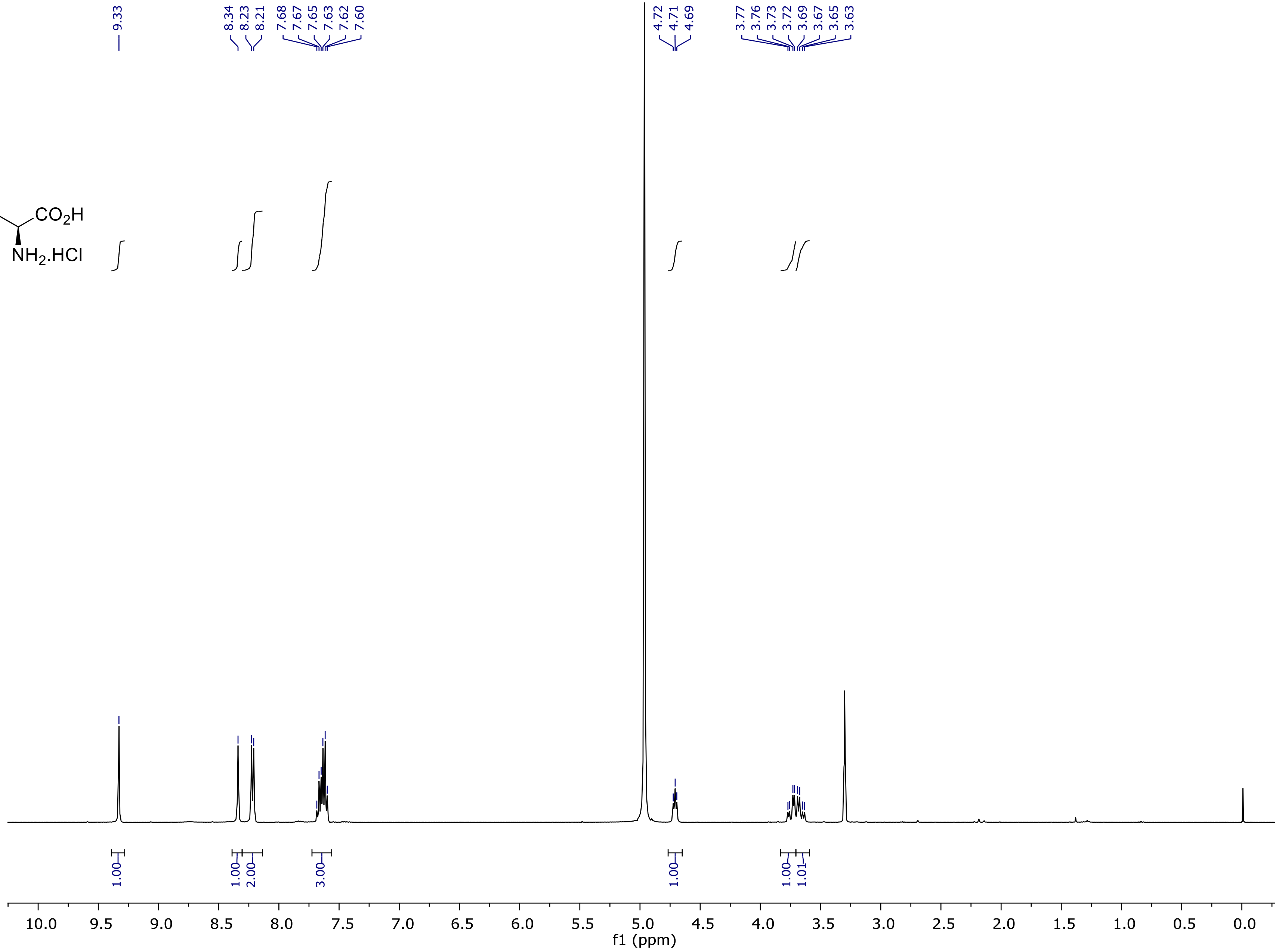

$^{13}C\{^{1}H\}$ NMR (101 MHz, CD_3OD)

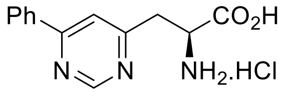

12i

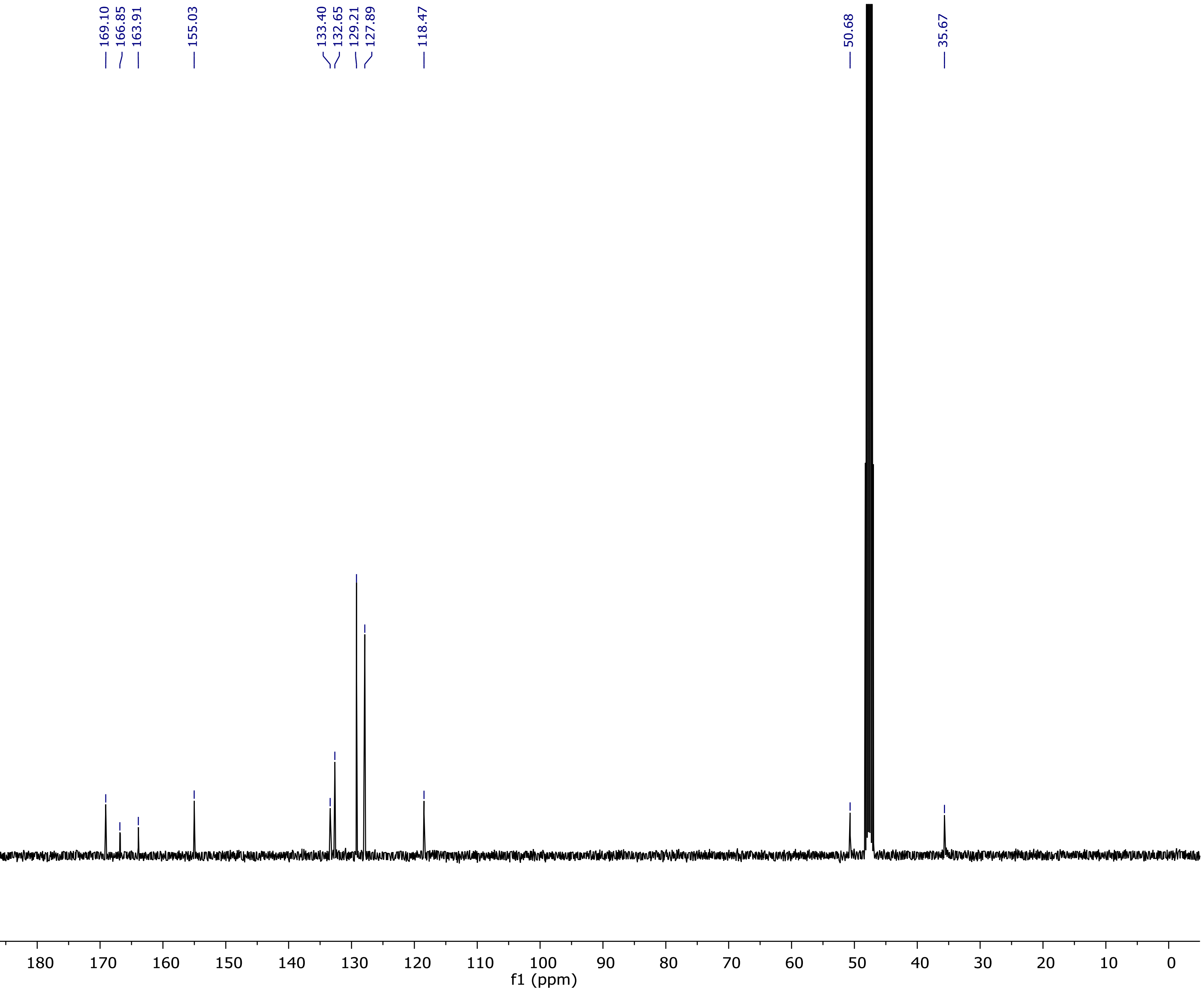

^{1}H NMR (400 MHz, CD_3OD)

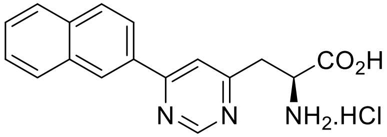

12j

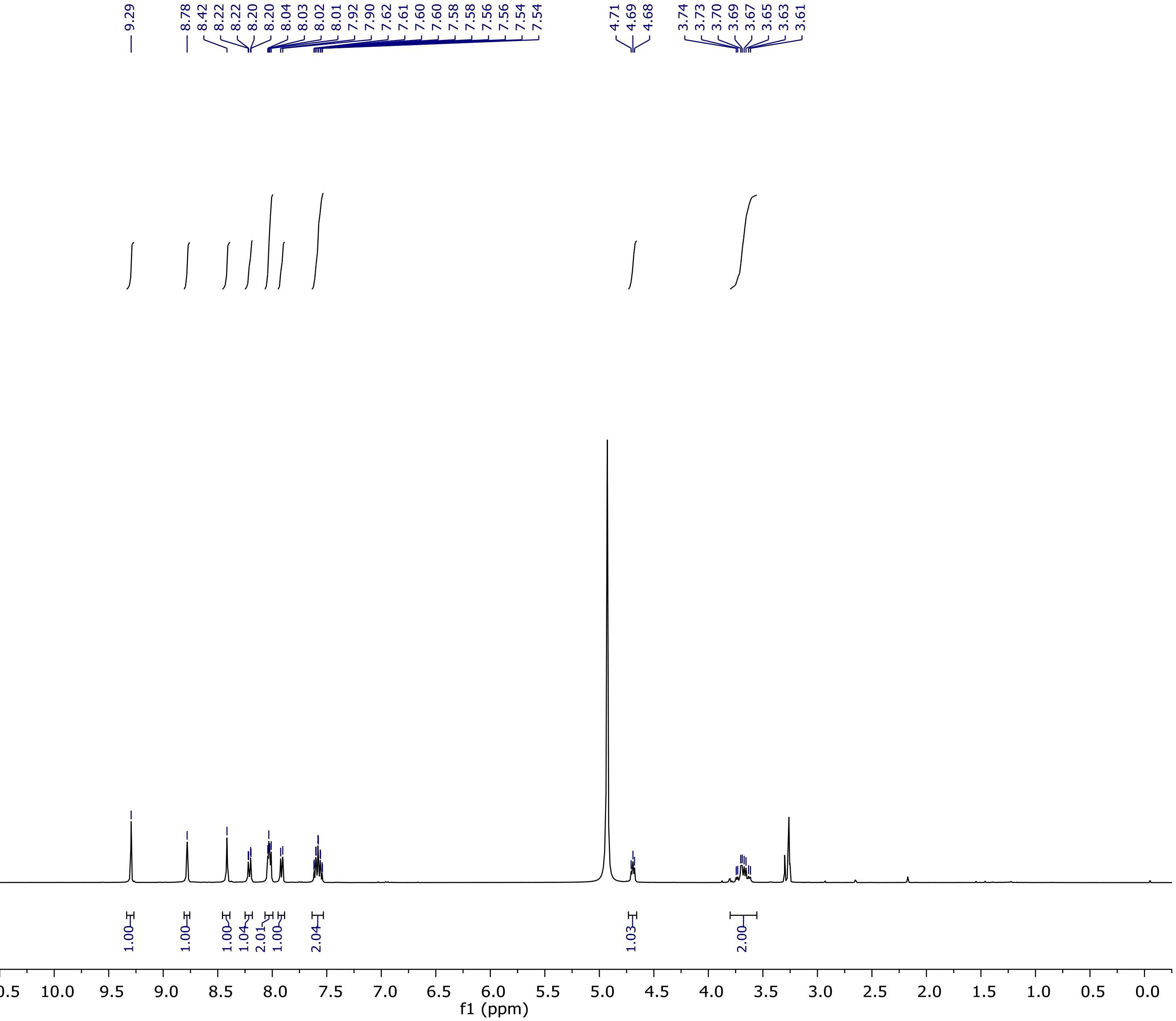

$^{13}C\{^{1}H\}$ NMR (101 MHz, CD_3OD)

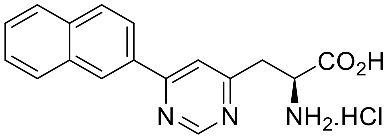

12j

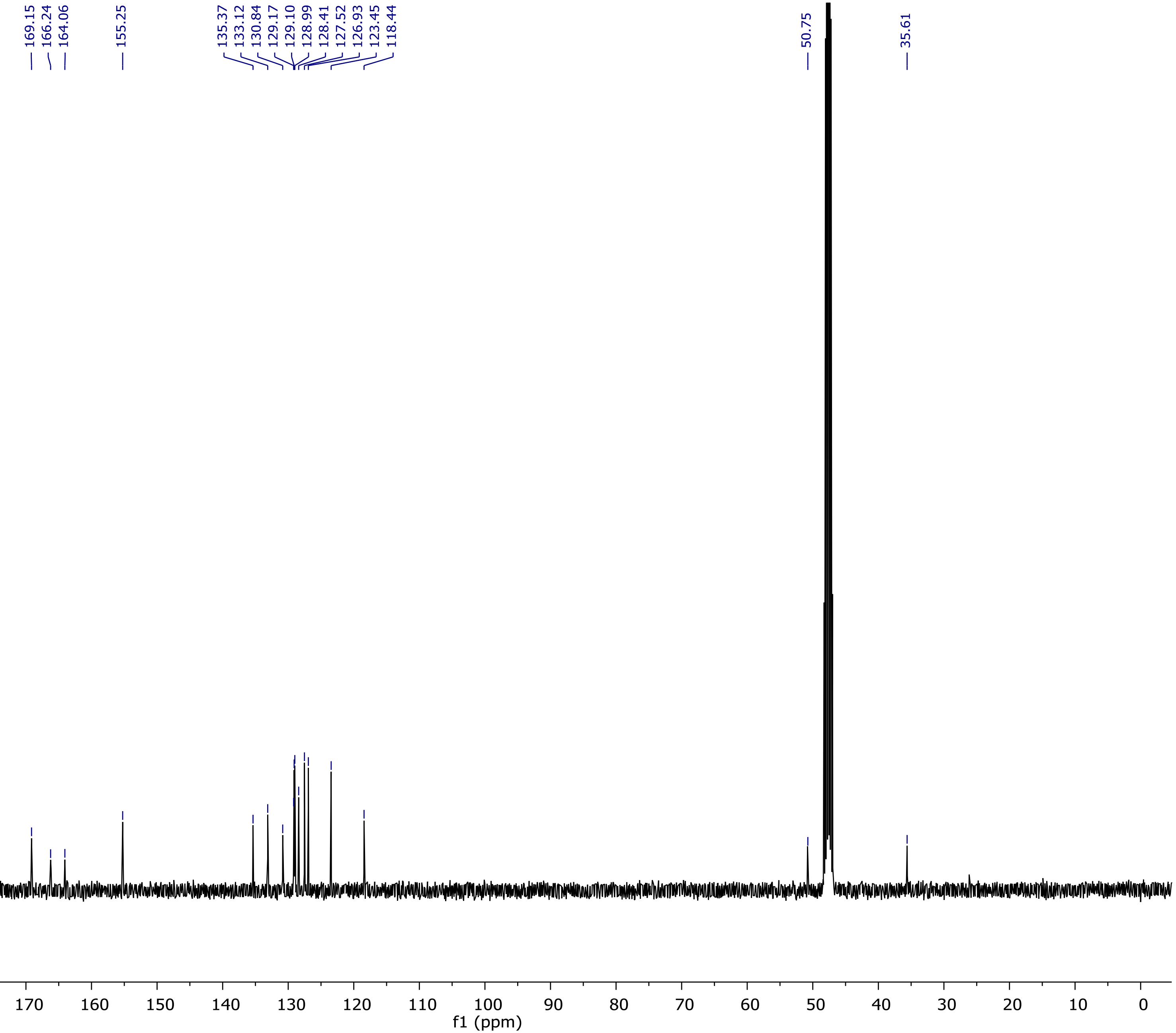

^{1}H NMR (400 MHz, CD_3OD)

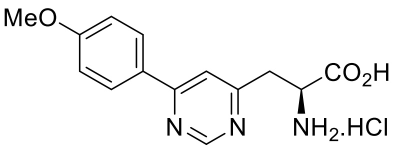

12k

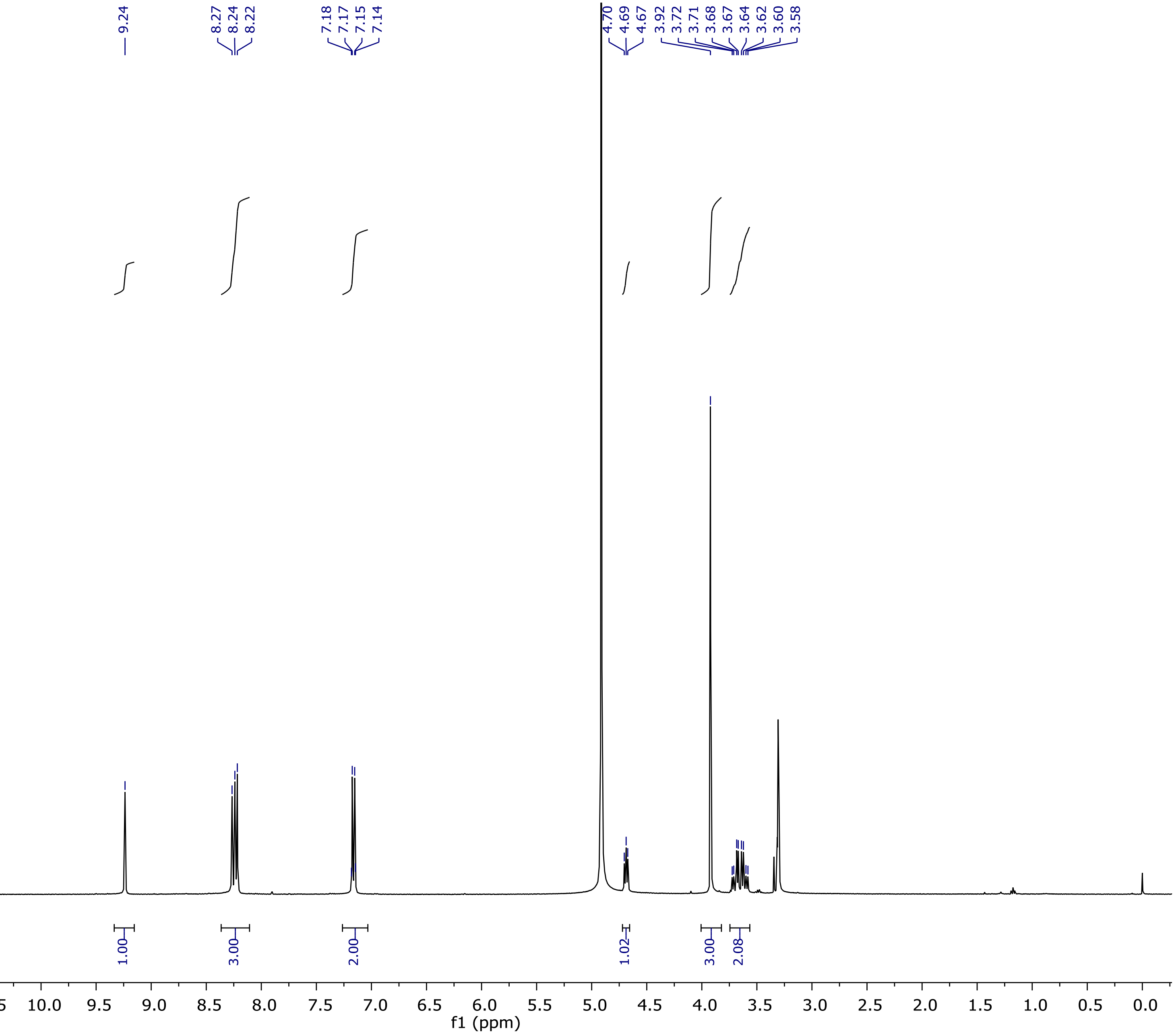

$^{13}C\{^1H\}$ NMR (101 MHz, CD_3OD)

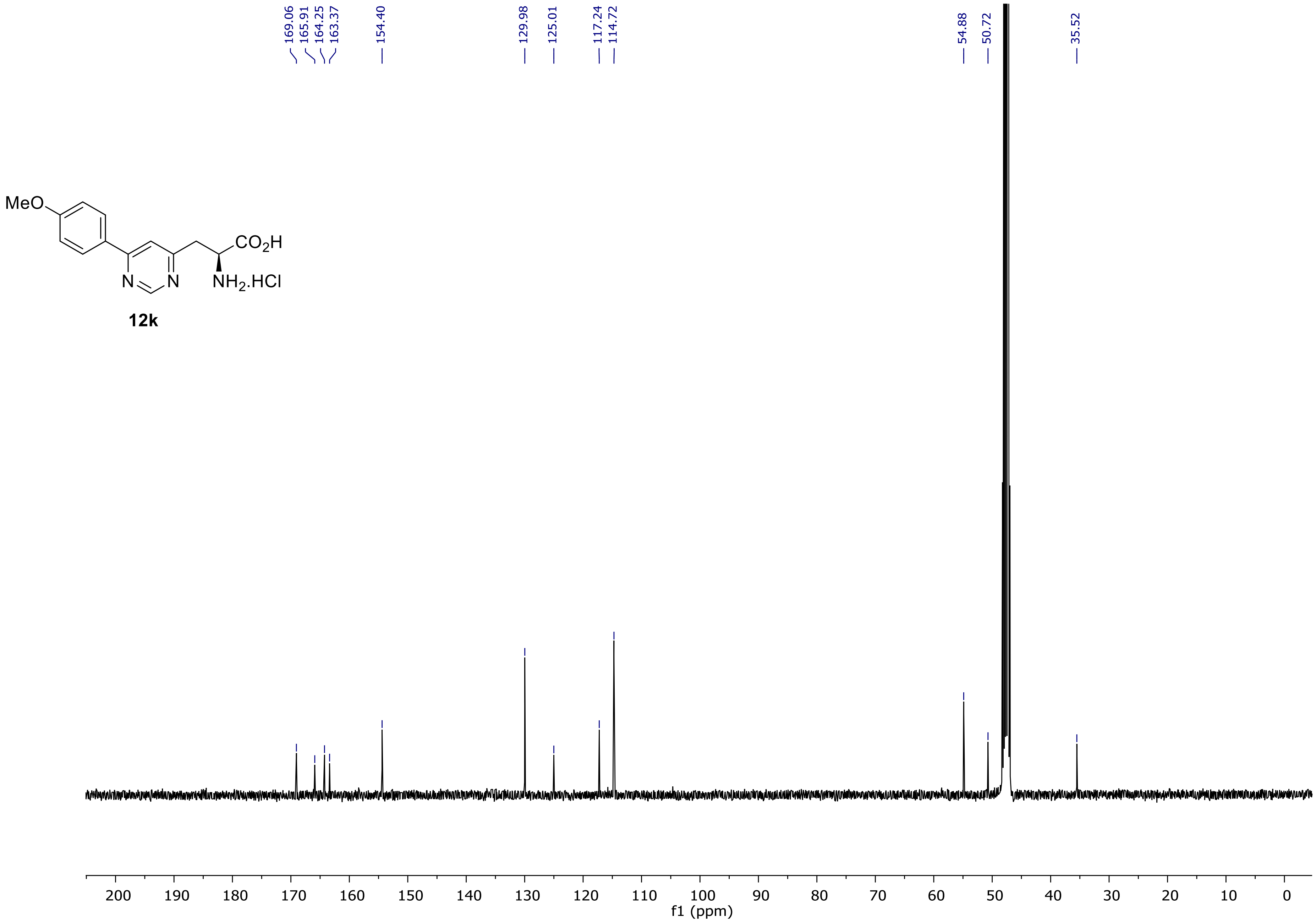

^{1}H NMR (400 MHz, CD_3OD)

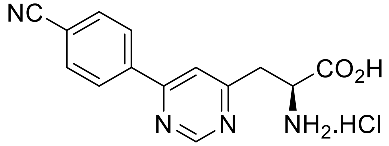

12l

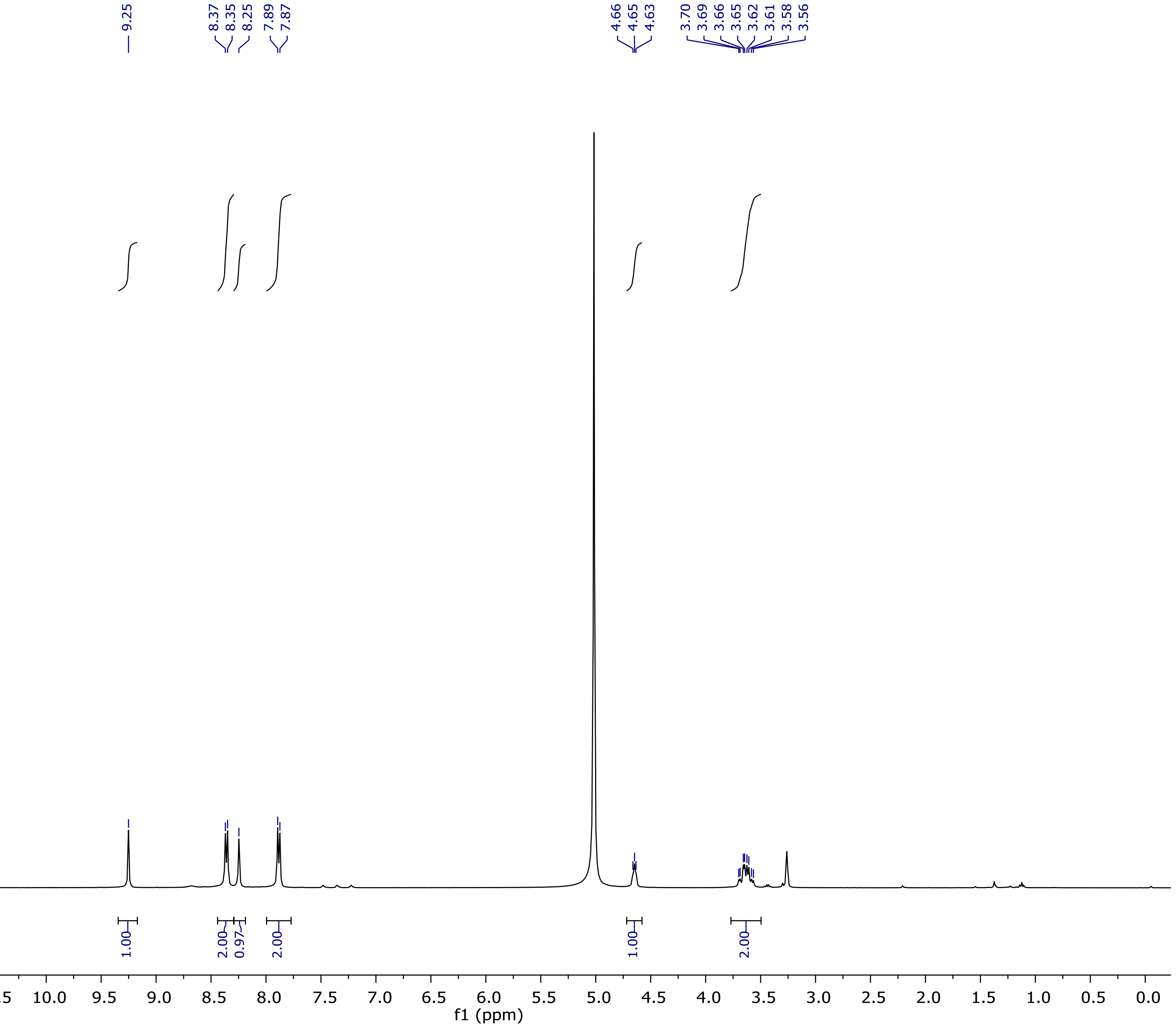

$^{13}C\{^{1}H\}$ NMR (101 MHz, CD_3OD)

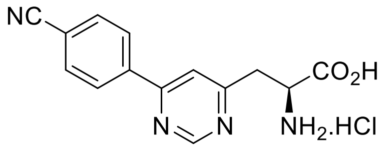

12l

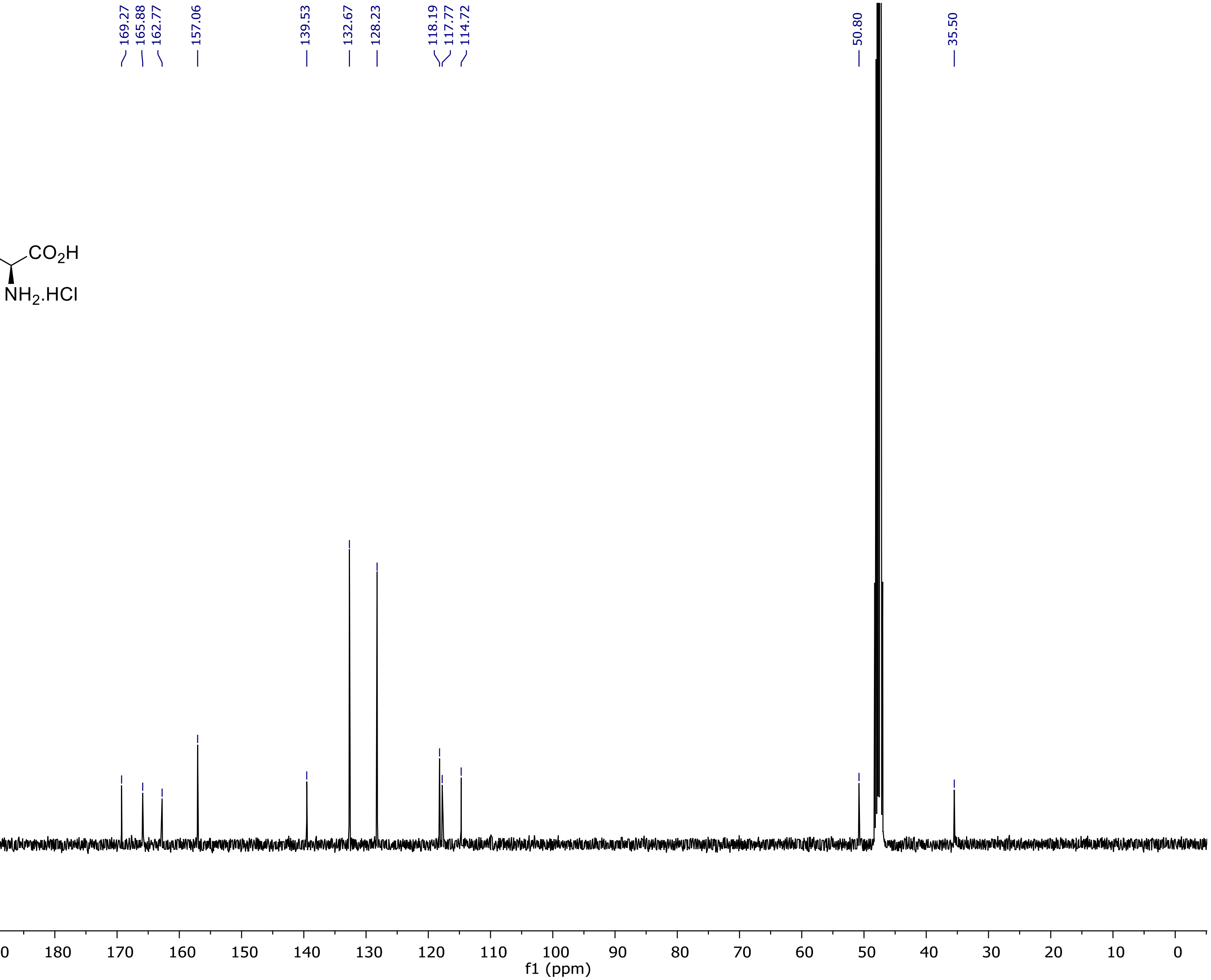

Supplement: Supplementary file 1 — jo3c01437_si_001.pdf [file jo3c01437_si_001.pdf]
